# Nuclear AURKA acquires kinase-independent transactivating function to enhance breast cancer stem cell phenotype

**DOI:** 10.1038/ncomms10180

**Published:** 2016-01-19

**Authors:** Feimeng Zheng, Caifeng Yue, Guohui Li, Bin He, Wei Cheng, Xi Wang, Min Yan, Zijie Long, Wanshou Qiu, Zhongyu Yuan, Jie Xu, Bing Liu, Qian Shi, Eric W.-F. Lam, Mien-Chie Hung, Quentin Liu

**Affiliations:** 1 Sun Yat-Sen University Cancer Center, State Key Laboratory of Oncology in South China, Collaborative Innovation Center for Cancer Medicine, Guangzhou; Institute of Cancer Stem Cell, Dalian Medical University, 9 West Section, Lvshun South Road, Dalian 116044, China; 2 Department of Laboratory Medicine, The First Affiliated Hospital, Sun Yat-Sen University, 58 Zhongshan Road II, Guangzhou 510080, China; 3 State Key Laboratory of Molecular Reaction Dynamics, Dalian Institute of Chemical Physics, Chinese Academy of Sciences, 457 Zhongshan Road, Dalian 116023, China; 4 The Third Affiliated Hospital, Sun Yat-Sen University, 600 Tianhe Road, Guangzhou 510630, China; 5 Cancer Hospital/Cancer Institute, College of Life Sciences and Institutes of Biomedical Sciences, Fudan University, 220 Handan Road, Shanghai 200433, China; 6 Department of Surgery and Cancer, Imperial College London, Du Cane Road, London W12 0NN, UK; 7 Department of Molecular and Cellular Oncology, The University of Texas MD Anderson Cancer Center, 1515 Holcombe Boulevard, Houston, Texas 77030, USA; 8 Center for Molecular Medicine and Graduate Institute of Cancer Biology, China Medical University, 6 HSUEH-SHIH Road, Taichung 40402, Taiwan

## Abstract

Centrosome-localized mitotic Aurora kinase A (AURKA) facilitates G2/M events. Here we show that AURKA translocates to the nucleus and causes distinct oncogenic properties in malignant cells by enhancing breast cancer stem cell (BCSC) phenotype. Unexpectedly, this function is independent of its kinase activity. Instead, AURKA preferentially interacts with heterogeneous nuclear ribonucleoprotein K (hnRNP K) in the nucleus and acts as a transcription factor in a complex that induces a shift in *MYC* promoter usage and activates the *MYC* promoter. Blocking AURKA nuclear localization inhibits this newly discovered transactivating function of AURKA, sensitizing resistant BCSC to kinase inhibition. These findings identify a previously unknown oncogenic property of the spatially deregulated AURKA in tumorigenesis and provide a potential therapeutic opportunity to overcome kinase inhibitor resistance.

## Abstract (web summary)

Aurora kinase A is a mitotic kinase that facilitates G2/M events. Here the authors show that in breast cancer cells Aurora kinase A has a kinase-independent function in the nucleus by activating the MYC promoter in cooperation with hnRNP K, enhancing the breast cancer stem cell phenotype.

## Graphical abstract

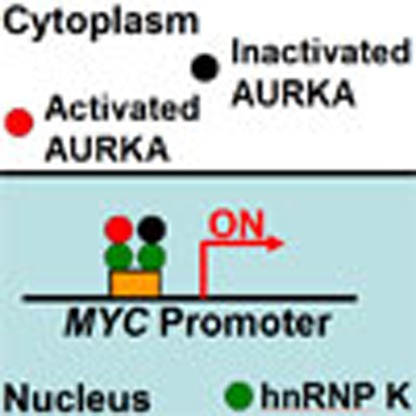

The oncogenic activation of kinases through mutation^1^ and amplification^2^ often leads to constitutively active kinase activity and cancer development^3^. This characteristic renders them addicted to constitutive kinase activation and thereby susceptible to kinase inhibition by small targeted molecules^4^. Despite the success of molecular targeted therapies that inhibit kinase activity in multiple cancers such as chronic myelogenous leukemia^5^ and lung cancer^6^, the development of resistance to kinase inhibition is inevitable, leading to cancer recurrence^7^. Missense mutations in the catalytic core of kinases account for the majority of clinically observed drug resistance cases^8^. Although new small molecule inhibitors can tolerate diverse mutations at the catalytic core, the blockage of kinase activation alone is often not sufficient to attain maximum therapeutic efficacy^9^.

Emerging evidence indicates that current therapeutic inhibitors do not effectively eliminate cancer stem cells (CSCs), thereby leading to drug resistance^10^. Several mechanisms of drug resistance have been proposed in CSCs, including tumour microenvironment nursing CSCs^11^, metabolic pathway alterations^12^ and epigenetic alterations^13^. However, the mechanisms that underlie therapeutic kinase inhibitor resistance remain elusive and require further elucidation.

Activation of Aurora kinase A (AURKA) plays an essential role in the control of mitosis progression, centrosome maturation/separation and mitotic spindle function^14^. AURKA has attracted a great deal of interest as a potential therapeutic target due to its overexpression in cancers^14^. Inhibitors of Aurora kinases, such as MLN8237 and PHA-739358, have been developed^15^, but were found to be moderately effective in preclinical and clinical studies^15^,^16^. These data suggest that a kinase-independent mechanism contributes to inhibitor insensitivity. There is emerging evidence to suggest that AURKA also promotes cancer development through mechanisms independently of its kinase activity^17^. Moreover, AURKA localizes to structures other than the mitotic apparatus during interphase to regulate neurite elongation and ciliary resorption, suggesting that AURKA possesses functions beyond its kinase activity^18^, and that inhibition of Aurora kinase alone may not be sufficient to repress AURKA oncogenic functions.

Previous study shows that the tumour tissues display nuclear AURKA staining^19^, which predicts a poorer clinical outcome in ovarian cancer^20^. Conversely, cytoplasmic localized AURKA consistently fails to enhance the H-Ras-induced transformation in BALB/c 3T3 A31-1-1 cells^21^. These studies suggest an oncogenic role of nuclear AURKA that might be independent of its kinase activity. Here we demonstrate that AURKA displays a kinase-independent function in the nucleus to activate the *MYC* promoter in cooperation with hnRNP K, enhancing the breast CSC phenotype.

## Results

### Nuclear AURKA enhances breast CSC phenotype

**Figure 1:**
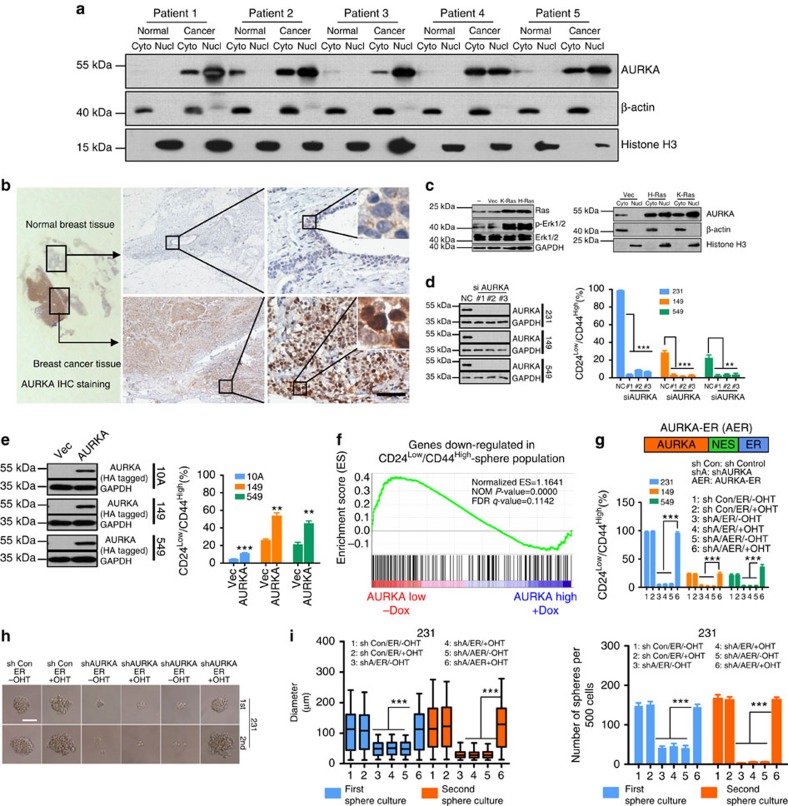
Nuclear AURKA enhances breast cancer stem cell phenotype. (**a**) Primary cells were extracted from breast cancer tissues and adjacent normal breast tissues. The cytoplasmic and nuclear protein lysates representing an equal number of cells were subjected to immunoblot (IB) analysis. (**b**) Representative IHC staining showing AURKA expression. Images were magnified with a × 4 or × 40 objective. Scale bar, 50 μm. (**c**) Mouse embryonic fibroblasts (MEFs) overexpressing K-Ras, H-Ras and the vector control (Vec) were analysed by IB for indicated antibodies (left panel). Cytoplasmic and nuclear lysates of WT (−)-, vector control (Vec)-, K-Ras- and H-Ras-overexpressed MEFs were subjected to IB analysis (right panel). (**d**) MDA-MB-231, SUM149 or BT549 cells were treated with AURKA siRNAs for 96 h. Adherent cells were collected for IB analysis (left panel), CD24/CD44 staining and CD24^low^/CD44^high^ population analysis via flow cytometry (right panel). (**e**) HA-tagged AURKA was overexpressed in MCF-10A, SUM149 and BT549 cells through lentivirus-mediated gene transfer. Puromycin (1 μg ml^−1^) selected cells were collected for IB analysis (left panel), CD24/CD44 staining and CD24^low^/CD44^high^ population analysis by using flow cytometry (right panel). (**f**) Gene expression data acquired from a public database (GEO ID: GSE23541, the group of AURKA+Dox and AURKA-Dox) were subjected to GSEA using the gene expression signature that was downregulated in the CD24^low^/CD44^high^-sphere population. (**g**) MDA-MB-231 cells were co-transfected with shRNA vector (sh Control or shAURKA) and the overexpression vector (ER or AER) via lentivirus-mediated gene transfer. Puromycin (1 μg ml^−1^) and blasticidin (2 μg ml^−1^) double selected cells were subjected to CD24/CD44 staining and analysis via flow cytometry. (**h**) Cells derived from **g** were subjected to mammosphere culture assay (6 days, upper panel) and the secondary passaging (additional 6 days, lower panel). Scale bar, 100 μm. (**i**) Data from three independent experiments derived from **g** were used for quantitative analysis of mammosphere size (diameter, Φ) and mammosphere number. Left panel shows distribution pattern of mammosphere size from MDA-MB-231 (Kruskal–Wallis test followed by Dunn's multiple comparison test, ***P<0.001). Right panel shows the number of mammospheres (Φ>60 μm). Bars represent the means±s.e.m. of three independent experiments (analysis of variance (ANOVA) followed by least significant difference (LSD) test; *P<0.05, **P<0.01, ***P<0.001).

We first examined cytoplasmic and nuclear expression of AURKA in breast cancer and adjacent normal tissues (Fig. 1a). AURKA was detected in the cytoplasm in normal breast tissue. In contrast, AURKA was also highly expressed in the nuclear fraction of breast cancer tissue. Consistent with this, a similar expression pattern of nuclear AURKA was observed with immunohistochemistry (IHC) staining (Fig. 1b) and in the nuclear fraction of all cancer cells lines tested (Supplementary Fig. 1a). The cytoplasmic AURKA level was lower in breast cancer cells compared with the non-transformed MCF-10A cells. Immunofluorescence staining (Supplementary Fig. 1b–f) of AURKA showed results similar to those of both immunoblotting (Supplementary Fig. 1a) and IHC (Fig. 1b). These data indicated that the nuclear localization of AURKA would be important during cancer development. We found that oncogenic transformation of primary mouse embryonic fibroblasts by overexpressing K-Ras (G12V mutant) or H-Ras (G12V mutant; Fig. 1c left panel) increased both cytoplasmic and nuclear AURKA expression (Fig. 1c right panel). Importantly, the ratio of nuclear/cytoplasmic AURKA was significantly increased in Ras-transformed cells compared with the wild-type (WT) counterpart (Fig. 1c right panel).

Previous studies have established that overexpression of AURKA causes drug resistance^22^,^23^,^24^, promotes metastases^25^,^26^ and predicts poor prognosis for breast cancer^27^,^28^. We next investigated whether nuclear AURKA is involved in the regulation of breast CSC (BCSC) phenotype. On AURKA depletion (Fig. 1d left panel), the putative BCSC CD24^low^/CD44^high^ cell population was significantly reduced in all breast cancer cell lines (Fig. 1d right panel). Conversely, CD24^low^/CD44^high^ population was significantly increased following overexpression of AURKA (Fig. 1e). To further confirm this, we analysed BCSC by evaluating their aldehyde dehydrogenase activity^29^. Similarly, the ratio of ALDEFLUOR (+) population was positively correlated with the expression of AURKA (Supplementary Fig. 1g,h). We also performed gene set enrichment analysis (GSEA) and found that cells expressing low levels of AURKA showed a significant enrichment of genes that are downregulated in the CD24^low^/CD44^high^-sphere population (Fig. 1f). We next investigated the role of nuclear localized AURKA in enhancing BCSC phenotype. AURKA was fused with the hormone-binding domain of oestrogen receptor (ER) via nuclear export sequence (NES)^21^ and was abbreviated as AER (Fig. 1g upper panel). Fusion with ER did not significantly affect the kinase activity of AURKA, as indicated by expression of p-p53 (S315) and p-histone H3 (S10)^30^,^31^ (Supplementary Fig. 1i). On 4-hydroxytamoxifen (OHT) stimulation, AER re-localized to the nuclear fraction (Supplementary Fig. 1j,k). The CD24^low^/CD44^high^ population was markedly suppressed following the depletion of endogenous AURKA by small interfering RNA (siRNA) and the empty ER vector could not rescue this cell population in either the presence or the absence of OHT (Supplementary Fig. 1l and Fig. 1g lower panel). Interestingly, expression of cytoplasmic AURKA (without OHT) also failed to rescue the loss of the CD24^low^/CD44^high^ population, whereas the OHT-induced nuclear-localized AURKA effectively restored the CD24^low^/CD44^high^ population (Fig. 1g lower panel). Similar phenomena were observed in mammosphere culture (Supplementary Fig. 1m,n and Fig. 1h,i). We also found that sphere formation in the secondary passage was restored only if AER was expressed in the nucleus (Supplementary Fig. 1m,n and Fig. 1h,i). In addition, the cells used for mammosphere culture and secondary passage proliferated normally in adherent culture (Supplementary Fig. 1o,p) regardless of the cellular localization of AER; these results suggested that the observed effects were likely to be linked to self-renewal.

### AURKA displays transcriptional activity

**Figure 2:**
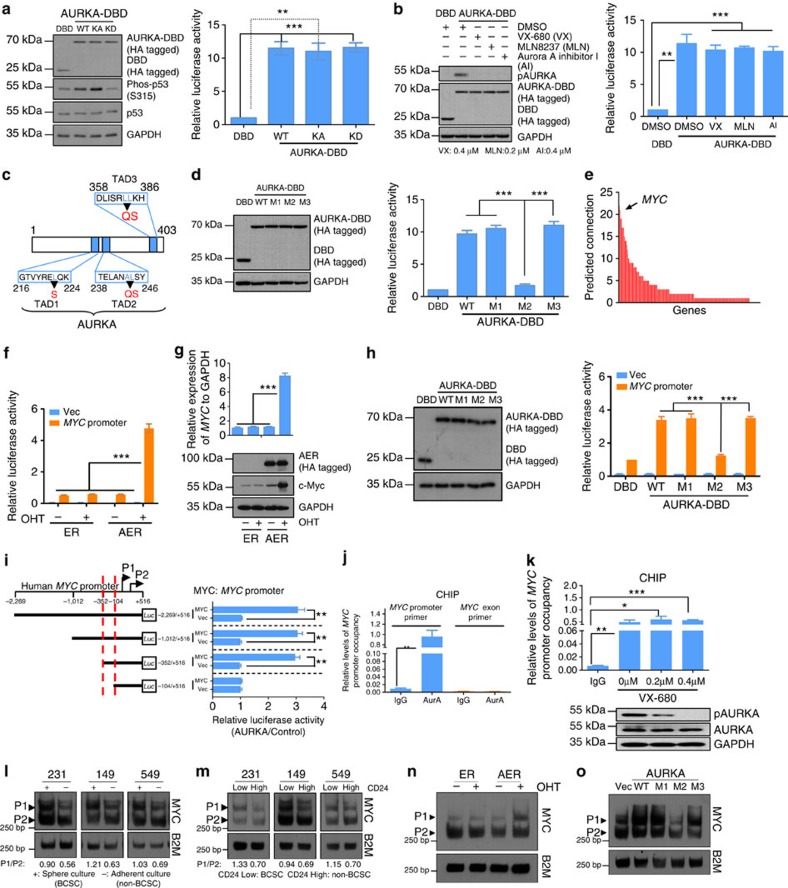
Nuclear AURKA activates *MYC* transcription and induces a shift in *MYC* promoter usage. (**a**) DBD/AURKA-DBD, DBD-reporter and pRL-TK were co-transfected into 293T for 24 h. Immunoblotting (IB; left panel) and dual-luciferase reporter (right panel) analysis were performed. (**b**) DBD/AURKA-DBD, DBD-reporter and pRL-TK were co-transfected into 293T cells. After 12 h, cells were treated with VX-680, MLN8237 or Aurora-A inhibitor I for 12 h. IB (left panel) and dual-luciferase reporter assay (right panel) were performed. (**c**) Diagram showing potential TADs in AURKA. (**d**) DBD/AURKA-DBD, DBD-reporter and pRL-TK were co-transfected into 293T cells for 24 h. IB (left panel) and dual-luciferase reporter assay (right panel) were performed. (**e**) STRING analysis showing connections among AURKA-regulated genes. (**f**) ER/AER, *MYC* promoter/basic reporter and pRL-TK were co-transfected into MDA-MB-231 cells. After 6 h, AURKA nuclear translocation was induced by treating cells with 200 nM OHT for 18 h. Dual-luciferase reporter assay was performed. (**g**) Treatment was the same as in **f**. Expression of indicated genes was determined via real-time PCR and IB analysis. (**h**) Treatment was similar to **d**, except for using *MYC* promoter reporter instead of DBD reporter. IB (left panel) and a dual-luciferase reporter (right panel) analysis were performed. (**i**) AURKA or empty vector was co-transfected into MDA-MB-231 cells with truncated *MYC* promoter (MYC) or basic reporter (Vec) along with pRL-TK. After 24 h, dual-luciferase reporter assays were performed. (**j**) MDA-MB-231 cells were subjected for ChIP analysis of *MYC* promoter occupancy. Results were normalized with the input. (**k**) VX-680-treated MDA-MB-231 cells (48 h) were subjected for ChIP analysis of *MYC* promoter occupancy. (**l**) Cells were cultured in suspended or adherent condition with same medium for 7 days. S1 nuclease protection assay (SNPA) was performed to examine *MYC* P1 and P2 transcripts. Control gene, *β-2-microglobulin (B2M)*, mRNA in parallel identical samples were also determined. (**m**) BCSC and non-BCSC populations were isolated according to CD24 expression. SNPA was performed. (**n**) ER/AER plasmids were transfected into 293T cells. After 6 h, AURKA nuclear translocation was induced by treating cells with 200 nM OHT for 18 h. SNPA was performed. (**o**) DBD/AURKA-DBD were transfected into 293T cells for 24 h. SNPA was performed. Bars represent the means±s.e.m. of three independent experiments (analysis of variance (ANOVA) followed by least significant difference (LSD) test; *P<0.05, **P<0.01, ***P<0.001).

To determine the transcriptional activity of AURKA, we fused GAL4 DNA-binding domain (DBD) to AURKA and expressed it in 293T cells with a luciferase reporter containing GAL4 DNA-binding sites upstream of a TATA box-containing minimal promoter. Expression of AURKA (WT)-DBD protein increased reporter activities by nearly tenfold compared with the vector control (DBD only; Fig. 2a), suggesting that AURKA transactivates gene expression. Interestingly, the expression of the KA (kinase active) or KD (kinase dead) mutants of the AURKA-DBD protein also increased the reporter activity to levels comparable to that induced by AURKA (WT)-DBD (Fig. 2a). We further tested the transactivation activity of AURKA (WT)-DBD in the presence of three different Aurora kinase inhibitors (Fig. 2b). In agreement, inhibition of AURKA kinase activity did not abolish its transactivating function. Together, these results suggest that AURKA possesses transactivation activity in a kinase-independent manner.

We next determined whether AURKA contains transactivating domains. We identified three putative nine amino acid transactivation domains (9aaTAD) in AURKA^32^ (Fig. 2c) and established their respective hydrophobicity profiles (Supplementary Fig. 2a blue line). To determine the transactivating activity of each putative 9aaTAD, the hydrophobic residues at position 6 and/or 7 were substituted with hydrophilic amino acids (Supplementary Fig. 2a red line)^33^. Mutation of the second (M2, A234Q/L244S) but not the first (M1, L222S) or the third (M3, L363A/L364S) 9aaTAD reduced the transactivating activity of AURKA (Fig. 2d), indicating that the second 9aaTAD is responsible for the transactivating activity of AURKA.

### Nuclear AURKA activates *MYC* transcription

To identify the downstream transcriptional targets of AURKA, RNA-sequencing analysis was performed on paired messenger RNA samples isolated from AURKA-overexpressed MCF-10A cells and control (Supplementary Table 1). Expression of a fraction of all identified genes was validated in MCF-10A and MDA-MB-231 cells (Supplementary Fig. 2b,c). These differentially expressed genes were then subjected to STRING analysis^34^. STRING analysis indicated that the oncogene *MYC* connected with a portion of AURKA-modulated genes with high confidence (>0.7; Fig. 2e and Supplementary Table 2) and might function as a hub molecule. Owing to the role of c-Myc in regulating diverse cellular functions^35^, we speculated that *MYC* might be a critical downstream target of AURKA that mediates the BCSC-related function.

We next examined whether AURKA regulates *MYC* gene expression at the transcriptional level. *MYC* mRNA was downregulated on AURKA depletion by siRNA and enhanced by the overexpression of AURKA in MDA-MB-231 cells (Supplementary Fig. 2d,e). Interestingly, WT AURKA, as well as KA and KD mutants, upregulated *MYC* mRNA expression at comparable levels (Supplementary Fig. 2e). Similar observation was made in BT549 and SUM149 cells (Supplementary Fig. 2f–i). Although AURKA depletion decreased the basal level of c-Myc, the half-life of c-Myc was not affected (Supplementary Fig. 2j). Furthermore, we found that AURKA expression induced the activity of the *MYC* promoter (from −2,269 to +516; Supplementary Fig. 2k,l), which was more likely to be dependent on the nuclear localization of AURKA (Fig. 2f,g) than its kinase activity (Supplementary Fig. 2l).

We further compared the luciferase reporter activity of the three mutants and found that the AURKA M2 mutant failed to increase *MYC* transcription as compared with M1 and M3 mutants (Fig. 2h and Supplementary Fig. 2m). We then determined the region of *MYC* promoter that is required for AURKA*-*activated *MYC* transcription. AURKA failed to activate transcription at *MYC* promoter when −352 to −104 region of *MYC* promoter was deleted (Fig. 2i). Results from chromatin immunoprecipitation (ChIP) assays further indicated that AURKA interacted with −352 to −104 region of *MYC* promoter (Fig. 2j). Consistent with these results, the interaction between AURKA and the *MYC* promoter was unaffected by the inhibition of AURKA kinase activity (Fig. 2k).

### Nuclear AURKA induces a shift in *MYC* promoter usage

The human *MYC* gene mainly transcripts from its P1 and P2 promoters^36^. Owing to the role of AURKA in BCSC, we first examined the relative utilization of P1 and P2 in BCSC and non-BCSC. S1 nuclease protection assay showed that the P1/P2 ratio was higher in the sphere, which is enriched in BCSC, compared with cells from adherent culture (Fig. 2l). Furthermore, CD24^Low^ or side population cells also displayed higher P1/P2 ratio compared with CD24^High^ or non-side population cells (Fig. 2m and Supplementary Fig. 2n). We next examined whether nuclear AURKA regulates the differential utilization of P1 and P2 promoters. As shown in Fig. 2n, nuclear, but not cytoplasmic, AURKA increased the P1/P2 ratio, inducing a shift in *MYC* promoter usage. Consistently, mutation of the second (M2, A234Q/L244S) AURKA transactivation domain (TAD) abolished the AURKA-induced shift in *MYC* promoter usage (Fig. 2o).

### AURKA and hnRNP K efficiently interact in the nucleus

**Figure 3:**
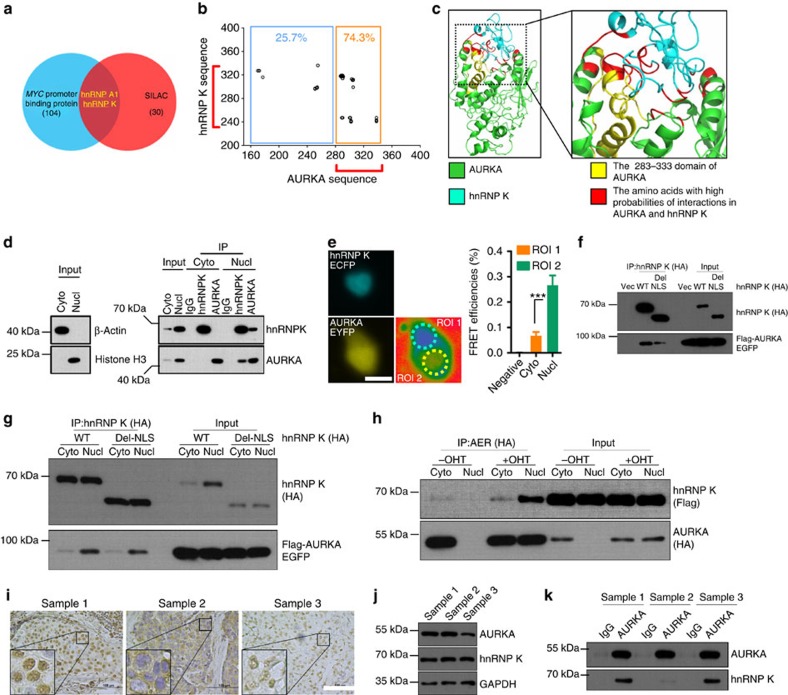
AURKA and hnRNP K efficiently interacts in the nucleus. (**a**) AURKA-interacting proteins were identified using SILAC assay (red area). *MYC* promoter-regulating proteins were previously reported (blue area). Proteins presented in both categories were selected for further analysis. (**b**) Thirty-five combinations of the amino acid derived from AURKA and hnRNP K with high probabilities of interactions were used to compile a dot plot. The amino acids in orange box were located in the nucleotide 283–333 region of the AURKA sequence. (**c**) The simulated interaction diagram of AURKA and hnRNP K. KI domain was shown. (**d**) Nuclear/cytoplasmic protein fractions of MDA-MB-231 cells were subjected to IP and immunoblotting (IB) using antibodies as indicated. (**e**) hnRNP K-ECFP- and AURKA-EYFP-co-transfected 293T cells were subjected to FRET efficiency analysis. ROI1 and ROI2 were selected for the analysis in the cytoplasmic and nuclear regions, respectively. Enhanced cyan fluorescent protein (ECFP)- and enhanced yellow fluorescent protein (EYFP)-co-transfected cells were used as negative controls. Scale bar, 50 μm. (**f**) Twenty micrograms of WT or NLS deletion mutant hnRNP K were co-transfected with Flag-AURKA–enhanced green fluorescent protein (EGFP) into 293T cells for 24 h. Cells were then subjected to IP and IB using antibodies as indicated. (**g**) Twenty micrograms of WT or NLS deletion mutant hnRNP K were co-transfected with Flag-AURKA–EGFP in 293T for 24 h. Cytoplasmic and nuclear proteins were separated and subjected to IP and IB using antibodies as indicated. (**h**) Twenty micrograms of AER was co-transfected with Flag-tagged hnRNP K into 293T cells. After 6 h, AURKA nuclear translocation was induced by treatment with 200 nM OHT for 18 h. Cytoplasmic and nuclear proteins were then separated and subjected to IP and IB using antibodies as indicated. (**i**) Samples 1–3 were subjected to IHC staining of AURKA. Scale bar, 100 μm. (**j**) The lysates of samples 1–3 were subjected to IB using antibodies as indicated. (**k**) The lysates of samples 1–3 were subjected to IP and IB using antibodies as indicated. Bars represent the means±s.e.m. of three independent experiments (analysis of variance (ANOVA) followed by least significant difference (LSD) test; *P<0.05, **P<0.01, ***P<0.001).

We next identified the proteins that were critical for AURKA-promoted activation of *MYC* promoter. We determined the proteins interacting with AURKA by SILAC^37^. We searched for proteins that interacted with AURKA and are also promoter regulatory proteins of MYC (Supplementary Table 3)^36^. We identified two heterogeneous nuclear ribonucleoproteins, hnRNP A1 and hnRNP K (Fig. 3a). Co-expression of AURKA and hnRNP K significantly increased *MYC* promoter activities compared with either alone, whereas co-expression of AURKA and hnRNP A1 did not show similar result (Supplementary Fig. 3a–d). As expected, there was no significant difference in the activation of the *MYC* promoter between KA and KD forms of AURKA (Supplementary Fig. 3e–g). We thus chose hnRNP K for further studies.

The interaction between AURKA and hnRNP K was validated via co-immunoprecipitation (co-IP) of ectopically expressed (Supplementary Fig. 3h,i) and endogenous (Supplementary Fig. 3j) proteins. This binding was not affected by mutation of S379 site in hnRNP K^38^ (Supplementary Fig. 3k). We further performed glutathione *S*-transferase (GST) pull-down assays with truncated forms of both proteins (Supplementary Fig. 3l,m). Results showed that hnRNP K KI domain interacts with AURKA 283–333 domain (Supplementary Fig. 3n,o). Moreover, molecular dynamics simulation revealed 35 combinations shown high interaction possibilities between AURKA and hnRNP K. Notably, the amino acids within AURKA 283–333 region accounted for 74.3% of these combinations (Fig. 3b). The amino acids responsible for the AURKA and hnRNP K interaction (red colour) displayed spatial proximity (Fig. 3c).

We next examined the location where AURKA and hnRNP K interact with each other. Although AURKA and hnRNP K were expressed in both the cytoplasm and the nucleus, their interaction was observed predominantly in the nucleus (Fig. 3d). The interaction between AURKA and hnRNP K in the nucleus was further supported by fluorescence resonance energy transfer (FRET) assay (Fig. 3e). hnRNP K contains two nuclear localization signals (NLSs), amino acids 21–37 and amino acids 323–361 (ref. 39). We next examined whether deletion of these two NLS might affect its interaction with AURKA. Deletion of two NLS (labelled as Del-NLS) partially relocated hnRNP K to the cytoplasm (Supplementary Fig. 3p), indicating that other way might allow hnRNP K to enter the nucleus in addition to these specific NLSs. Co-IP showed that deletion of two NLSs weakened the interaction between AURKA and hnRNP K (Fig. 3f). We also extracted the cytoplasmic and nuclear lysates from cells overexpressing the WT hnRNP K or Del-NLS mutant for co-IP studies. Both the WT hnRNP K and Del-NLS mutant interacted efficiently with AURKA in the nucleus (Fig. 3g), whereas their interactions with AURKA were very weak in the cytoplasm. We also examined the cytoplasmic and nuclear proteins extracted from AER-overexpressing cells, which were treated or untreated with OHT. Similarly, AURKA and hnRNP K showed higher interaction efficiencies in the nuclear fraction compared with that in the cytoplasmic fraction (Fig. 3h). Furthermore, we performed co-IP using breast cancer tissues, which displayed different levels of nuclear/cytoplasmic AURKA expression. Sample 1 displayed strong nuclear AURKA staining, sample 2 showed strong cytoplasmic AURKA staining and sample 3 showed low nuclear AURKA staining (Fig. 3i,j). The interaction between AURKA and hnRNP K is strong when AURKA is predominantly expressed in the nucleus (Fig. 3k lower panel, compare lane 2 with 6), but the interaction is weak when AURKA is predominantly expressed in the cytoplasm (Fig. 3k lower panel, compare lanes 2 and 6 with lane 4). These data suggest that AURKA and hnRNP K preferentially interact in the nucleus.

### hnRNP K is required for AURKA to activate *MYC* transcription

**Figure 4:**
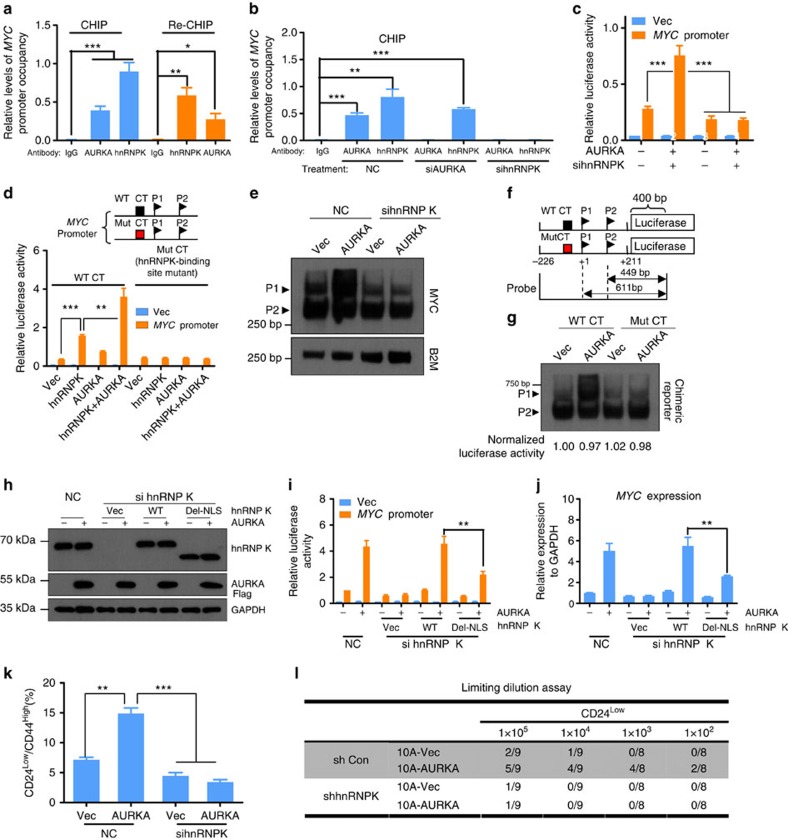
hnRNP K is required for AURKA to activate *MYC* transcription and enhance BCSC phenotype. (**a**) Chromatin from MDA-MB-231 cells was extracted for ChIP and re-ChIP analysis. Results were normalized by input. (**b**) MDA-MB-231 cells were transfected with siRNA against negative control (NC), AURKA or hnRNP K for 48 h. ChIP assays were performed. (**c**) MDA-MB-231 cells overexpressing HA-tagged AURKA were transfected with hnRNP K or NC siRNA. After 24 h, cells were transfected with *MYC* promoter reporter (MYC) or basic reporter (Vec) along with pRL-TK for another 24 h. Dual-luciferase reporter assay was performed. (**d**) Mutated reporter along with pRL-TK were co-transfected with AURKA or/and hnRNP K plasmids for 24 h. Dual-luciferase reporter assay was performed. (**e**) 293T cells overexpressing HA-AURKA were transfected with hnRNP K or NC siRNA for 48 h. S1 nuclease protection assay (SNPA) was performed to monitor *MYC* P1 and P2 transcripts. (**f**) Structure of chimeric gene consists of firefly luciferase and *MYC* promoter (−226/+211) (upper panel). Lower panel shows probes used for SNPA. ‘CT' represented CT element. The mutant chimeric gene was mutated at hnRNP K-binding site. (**g**) Chimeric gene or its mutant were co-transfected with AURKA and pRL-TK plasmids into 293T cells for 24 h. SNPA was performed using the probes shown in **f**. In parallel, a fraction of these cells was evaluated for *Renilla* luciferase activity, which reflects the transfection efficiency. (**h**,**i**,**j**) 293T cells were transfected with hnRNP K siRNA for 24 h. Cells were then co-transfected with WT or NLS-deleted hnRNP K, AURKA and *MYC* promoter or basic reporter along with pRL-TK for another 24 h. Transfected cells were harvested for immunoblotting (IB) (**h**), dual-luciferase reporter (**i**) and real-time PCR (**j**) analysis. (**k**) MCF-10A cells overexpressing HA-AURKA were transfected with hnRNP K or NC siRNA. After 48 h, cells were collected to analyse CD24/CD44. (**l**) hnRNP K was knocked down in AURKA-overexpressing or control 10A-K-Ras (G12V) cells. Cells were sorted according to CD24 expression. CD24^Low^ population was used to perform limiting dilution assays. Bar represented the means±s.e.m. of three independent experiments (analysis of variance (ANOVA) followed by least significant difference (LSD) test; *P<0.05, **P<0.01, ***P<0.001).

We used re-ChIP assays to determine whether AURKA and hnRNP K co-occupy the same region of the *MYC* promoter. We found that AURKA formed a complex with hnRNP K on the *MYC* promoter (Fig. 4a). Further, ChIP assays were performed following depletion of AURKA or hnRNP K by siRNA. AURKA depletion failed to abolish the binding of hnRNP K to the *MYC* promoter (Supplementary Fig. 4a and Fig. 4b), whereas hnRNP K silencing abolished AURKA binding. These results suggest that the binding of AURKA to the *MYC* promoter depends on hnRNP K. In addition, depletion of hnRNP K by siRNA also abolished the ability of AURKA to upregulate the expression of *MYC* (Fig. 4c and Supplementary Fig. 4b,c) in MDA-MB-231 cells. We further mutated the hnRNP K-binding sites in the *MYC* promoter (Supplementary Fig. 4d) and found that the mutant promoter was no longer activated in response to AURKA and/or hnRNP K overexpression (Fig. 4d and Supplementary Fig. 4e). Consistently, downregulation of hnRNP K or mutation of the hnRNP K-binding sites in the *MYC* promoter abolished the AURKA promoted utilization of *MYC* P1 (Fig. 4e–g). We also examined whether NLS-deleted hnRNP K rescues the *MYC* expression. NLS-deleted hnRNP K was less effective in rescuing the *MYC* expression than the WT hnRNP K (Fig. 4h–j). Moreover, phosphorylation-mimicking (S379D) and non-phosphorylatable (S379A) mutants of hnRNP K activated the *MYC* promoter at similar levels (Supplementary Fig. 4f), suggesting that phosphorylation of hnRNP K at Ser379 by AURKA is not required for activating *MYC* promoter.

### hnRNP K is required for AURKA to enhance BCSC phenotype

We next evaluated whether nuclear AURKA-enhanced BCSC phenotype was mediated by hnRNP K. Depletion of hnRNP K by siRNA suppressed the CD24^low^/CD44^high^ population to the same extent observed in MCF-10A and SUM149 cells in the presence or the absence of AURKA overexpression (Fig. 4k and Supplementary Fig. 4g). We therefore propose that the ability of AURKA to promote BCSC population depends on hnRNP K. To validate this, we expressed c-Myc at physiological levels from an exogenous WT or mutated *MYC* promoter (Mut CT) and then monitored mammosphere formation in these cells. A slightly increased expression of c-Myc (Supplementary Fig. 4h, comparing lanes 4 and 7 with lane 1) indicated that exogenous AURKA and hnRNP K were expressed at physiological levels. MDA-MB-231 cells harbouring exogenous WT, but not mutant, *MYC* promoter expressed higher levels of c-Myc when AURKA or/and hnRNP K were also expressed (Supplementary Fig. 4h, comparing lane 5 with 4, lane 8 with 7 and lane 11 with 10). The size and the number of spheres also indicated that only cells harbouring WT *MYC* promoter showed enhanced sphere formation in response to the expression of exogenous AURKA or/and hnRNP K (Supplementary Fig. 4i). Cells used in mammosphere formation assays were then subjected to adherent culture. These cells displayed similar proliferation kinetics (Supplementary Fig. 4j), indicating that the above-mentioned effects were probably linked to self-renewal.

Limiting dilution assays were performed in nonobese diabetic/severe combined immunodeficient mice, to analyse the potential role of hnRNP K in regulating nuclear AURKA-promoted BCSC property. The tumorigenicity of cells overexpressing AURKA was examined in the context of hnRNP K downregulation. The CD24^High^ population failed to generate tumours over a 3-month period with injection of <1 × 10^5^ cells (Supplementary Fig. 4k). In contrast, tumour growth was observed in the CD24^Low^ population when cell number was <1 × 10^5^ and became more apparent when AURKA was overexpressed (Fig. 4l), suggesting that AURKA plays a role in tumour initiation. The effect of AURKA on promoting tumour initiation was abolished by knocking down hnRNP K (Fig. 4l), further supporting the role of hnRNP K in AURKA-mediated BCSC property. The CD24^Low^ cells were isolated and subjected to adherent culture, to evaluate their proliferation potential. Overexpression of AURKA marginally increased proliferation rate relative to vector control, whereas downregulation of hnRNP K abolished the growth-promoting effects of AURKA and slightly decreased the proliferation relative to the vector control (Supplementary Fig. 4l). These results indicate that the dramatic reduction in tumorigenicity induced by knocking down hnRNP K was not caused by the mild proliferation attenuation that we observed in the adherent culture.

### AURKA NLS is required to enhance BCSC phenotype

**Figure 5:**
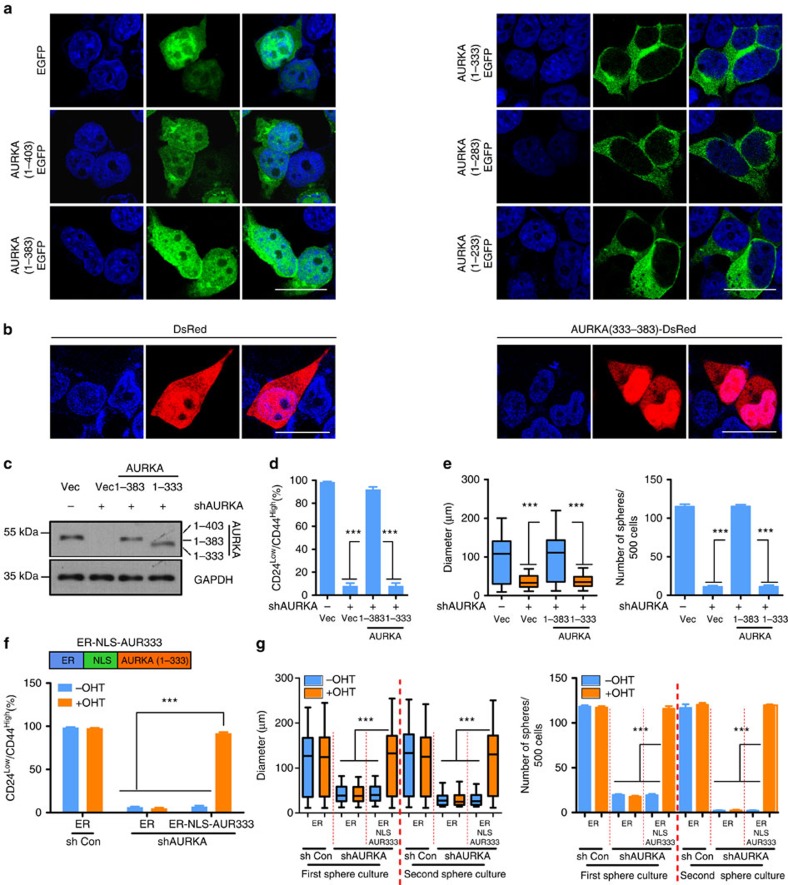
AURKA nuclear localization sequence is required to enhance breast cancer stem cell phenotype. (**a**) Enhanced green fluorescent protein (EGFP)-fused truncated forms of AURKA were transfected into 293T cells for 24 h. The localization of EGFP-fused truncated forms of AURKA were analysed via confocal microscopy. Scale bar, 25 μm. (**b**) DsRed-fused 333–383 region of AURKA was transfected into 293T cells for 24 h. The localization of DsRed-fused 333–383 region of AURKA was analysed by confocal microscopy. Scale bar, 25 μm. (**c**,**d**) MDA-MB-231 cells were co-infected with lentivirus expressing AURKA shRNA (shAURKA/sh Control) and the truncated protein (1–383 or 1–333). Puromycin (1 μg ml^−1^) and blasticidin (5 μg ml^−1^) selected cells were subjected to immunoblotting (IB) analysis (**c**) or CD24^low^/CD44^high^ population analysis via flow cytometry (**d**). (**e**) The cells in **c** were subjected to mammospheres culture assay for 6 days. The left panel shows the distribution pattern according to mammosphere size (Kruskal–Wallis test followed by Dunn's multiple comparison test, ***P<0.001). The right panel showed the number of Φ>60 μm mammospheres. (**f**) AURKA (1–333) was fused with ER via a nuclear localization sequence to generate ER-NLS-AUR333 fusion protein (upper panel). MDA-MB-231 cells were co-infected with lentivirus expressing AURKA shRNA (shAURKA/sh Control) and the fusion protein (ER-NLS-AUR333). The cells were then cultured in fresh medium for 48 h in the presence or absence of 200 nM OHT. Puromycin (1 μg ml^−1^) and blasticidin (5 μg ml^−1^) selected cells were harvested for CD24/CD44 staining and analysis via flow cytometry (lower panel). (**g**) The cells in **f** were cultured in mammosphere medium with or without 200 nM OHT. The cells were then photographed and quantified at the end of the first-round mammosphere culture (6 days) and the secondary passaging (additional 6 days). The left panel shows the distribution pattern of mammosphere size from MDA-MB-231 (Kruskal–Wallis test followed by Dunn's multiple comparison test, ***P<0.001). The right panel shows the number of mammosphere (Φ>60 μm). Data were presented as the means±s.e.m. of three independent experiments (analysis of variance (ANOVA) followed by least significant difference (LSD) test; *P<0.05; **P<0.01; ***P<0.001).

We also asked whether AURKA contains an NLS. We found that deletion of the amino acids 333–383 in AURKA blocked its nuclear localization (Fig. 5a and Supplementary Fig. 5a), indicating that this region contains NLS. These data also suggest that the NES of AURKA may reside in amino acids 1–333. We fused the putative NLS (amino acids 333–383 amino acid) to DsRed. Cells expressing DsRed alone demonstrated a nearly homogeneous distribution in 293T cells (Fig. 5b left panel), whereas DsRed-333–383 fusion protein was partially induced to localize to the nucleus (Fig. 5b right panel), indicating that this region of AURKA possesses nuclear localizing function. hnRNP K overexpression, inhibition of AURKA kinase activity or deletion of NLS of hnRNP K did not significantly affect the subcellular distribution of AURKA (Supplementary Fig. 5b–d).

Truncated forms of AURKA (1–383 and 1–333) were used to perform rescue assay, to examine the role of nuclear AURKA in regulating BCSC phenotype. Although both the 1–383 and 1–333 regions rescued the proliferation in adherent culture (Supplementary Fig. 5e), only 1–383 regions of AURKA rescued BCSC phenotype (Fig. 5c–e). To confirm this, amino acids 1–333 of AURKA were fused with ER via the SV40 T-antigen NLS, to generate fusion protein labelled as ER-NLS-AUR333 (Fig. 5f upper panel). In the absence of OHT, ER-NLS-AUR333 predominantly localized to the cytoplasm. However, following incubation with OHT, a fraction of ER-NLS-AUR333 proteins translocated to the nucleus (Supplementary Fig. 5f). We then investigated whether ER-NLS-AUR333 would reconstitute BCSC expansion following endogenous AURKA depletion by short hairpin RNA (shRNA; Supplementary Fig. 5g). Only nuclear-localized ER-NLS-AUR333 rescued the CD24^low^/CD44^high^ population (Fig. 5f lower panel and Supplementary Fig. 5h) and the ability to form spheres following depletion of endogenous AURKA (Fig. 5g). Furthermore, we found that the ability to form spheroids in secondary passage was retained only when the ER-NLS-AUR333 was expressed in the nucleus (Fig. 5g). The proliferation of the cells used to perform mammosphere culture could be rescued by ER-NLS-AUR333 in adherent culture regardless of its cellular localization, indicating that the above-mentioned effects were more probably linked to self-renewal (Supplementary Fig. 5i).

### Reducing nuclear AURKA sensitizes AKI-resistant BCSC

**Figure 6:**
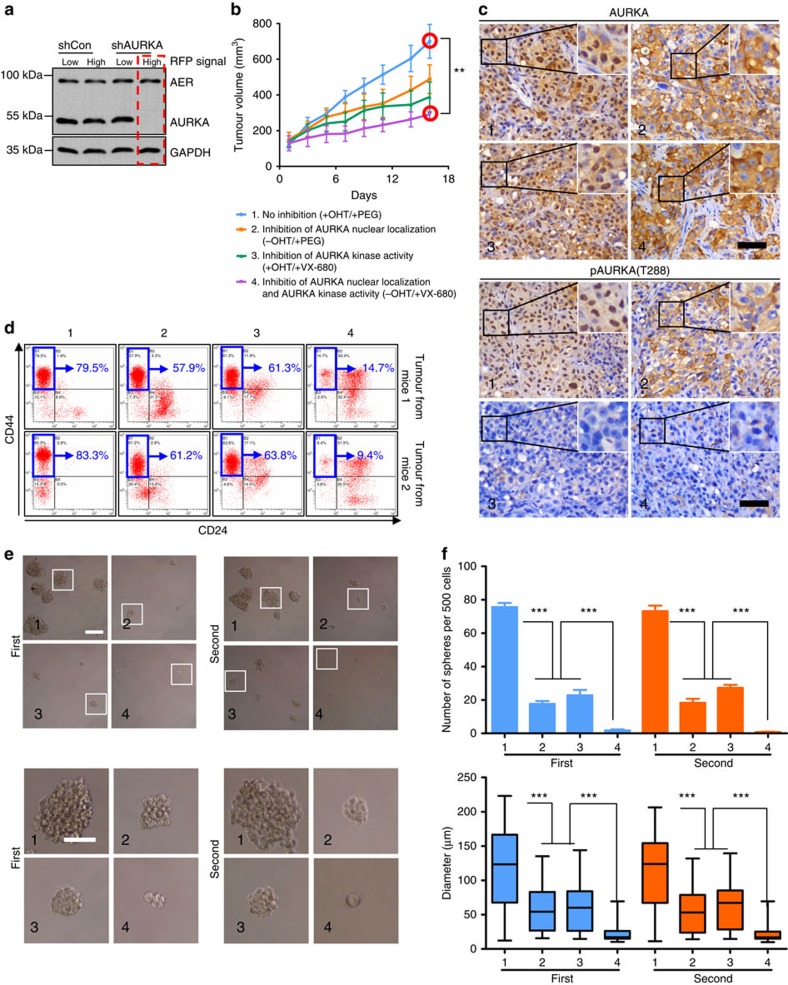
Blocking AURKA nuclear translocation enhances the anticancer effects of Aurora kinase inhibitor. (**a**) MDA-MB-231 cells expressing AURKA-ER were infected with lentiviral expressing AURKA shRNA or control. The cells were sorted for RFP (+) and the lysates were examined via immunoblotting (IB). (**b**) MDA-MB-231 cells sorted from **a** (red rectangle) were inoculated subcutaneously in nude mice. Mice treated with OHT were then administered the indicated treatments via intraperitoneal injection. Data were presented as the means±s.e.m.; *n*=5 per group. The statistical significance between tumour volumes was calculated using one-way analysis of variance (ANOVA) with Dunnett's multiple comparison tests (group1 versus group4). See also Supplementary Methods. (**c**) IHC staining was performed on tumours harvested at the end of the animal experiment (**b**). Scale bar, 100 μm. (**d**) Flow cytometry for the expression of CD44 and CD24 on single cells from tumour xenografts. (**e**) Mammosphere formation by single-cell suspensions derived from tumour xenografts (left panel). The mammosphere were used to perform a secondary passage (right panel). (**f**) Quantification of the diameters of the spheres from **e**. The upper panel shows the number of Φ>60 μm mammospheres. Statistical comparison was performed (lower panel) using the Kruskal–Wallis test followed by Dunn's multiple comparison test (***P<0.001). Data are presented as the means±s.e.m. of three independent experiments (analysis of variance (ANOVA) followed by least significant difference (LSD) test; *P<0.05; **P<0.01; ***P<0.001).

Endogenous AURKA was downregulated by shRNA (expressing red fluorescent protein (RFP)) in AER-expressing MDA-MB-231 cells. The resulting RFP-positive cells separated by fluorescence-activated cell sorting (FACS) were subjected to immunoblotting analysis (Fig. 6a red rectangle) and assayed for their ability to establish tumours in nude mice. Blocking AURKA nuclear localization (in the absence of OHT, group 2) attenuated tumour growth in xenograft model (Fig. 6b). Results from these experiments also established that the combination of AURKA inhibition (VX-680) and blocking AURKA nuclear localization had an additive tumour growth suppression effect (Fig. 6b, group 4). IHC staining of subcutaneous tumour sections confirmed that OHT regulated the subcellular localization of AURKA (Fig. 6c upper panel; image 2/4 compared with image 1/3), and that VX-680 suppressed AURKA phosphorylation at T288 (Fig. 6c lower panel; image 3/4 compared with image 1/2).

Next, we analysed the BCSC phenotype of cells isolated from the xenografts. The CD24^low^/CD44^high^ population was somewhat decreased when either the kinase activity or nuclear translocation of AURKA was blocked and even more substantially when both were inhibited (Fig. 6d). Sphere-formation assays also revealed similar synergetic anti-BCSC effects (first and second) when both AURKA kinase activity and nuclear translocation were blocked (Fig. 6e,f). These cells also displayed similar proliferative potential in adherent culture (Supplementary Fig. 6a), indicating that the effects observed in mammosphere culture were more likely to be specifically linked to self-renewal.

We further evaluated the effects of Aurora kinase inhibition on the oncogenic function of nuclear or cytoplasmic AURKA. Immunofluorescent staining showed that NES- and NLS-fused AURKA localized to the cytoplasm and the nucleus, respectively (Supplementary Fig. 6b). The kinase activity of these fusion proteins could be effectively inhibited by MLN8237 (Supplementary Fig. 6c). AURKA kinase inhibitor significantly suppressed the CD24^low^/CD44^high^ population in cells expressing AURKA-NES (Supplementary Fig. 6d,e) but not in cells expressing AURKA-NLS (Supplementary Fig. 6d,e). These results suggest that the oncogenic function of cytoplasmic AURKA relies predominantly on its kinase function, and that nuclear AURKA possesses kinase-independent activity, which is not repressed by the AURKA kinase inhibitor.

### Clinical relevance of nuclear AURKA expression

**Figure 7:**
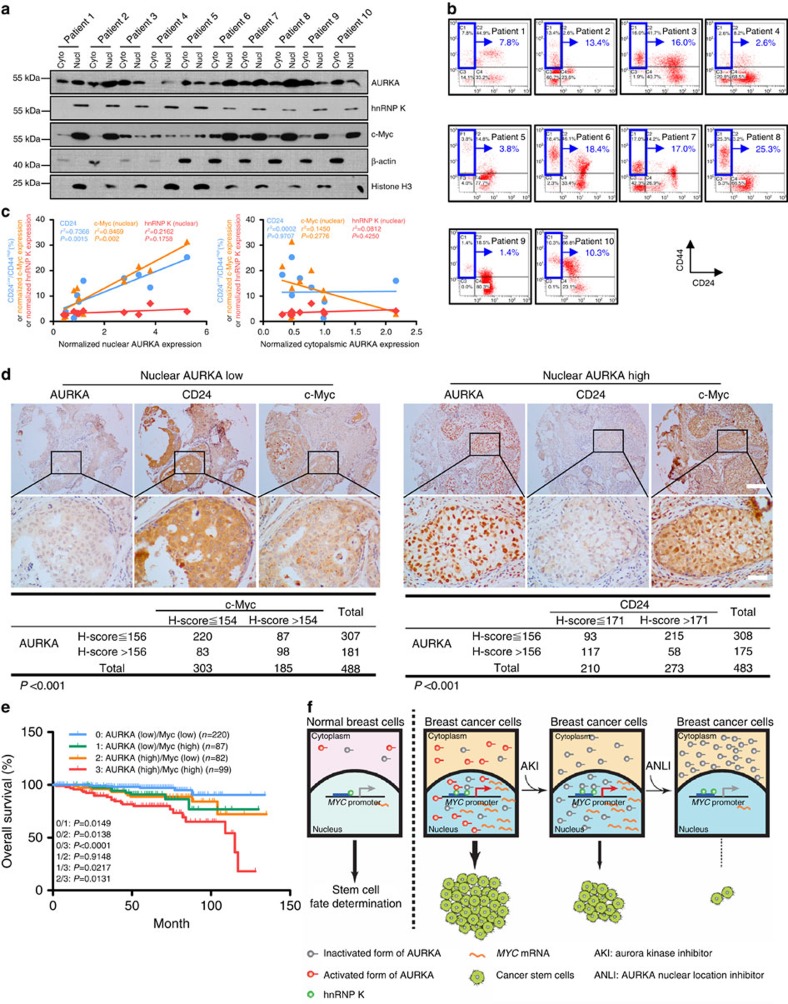
Clinical relevance of nuclear AURKA expression. (**a**,**b** and **c**) Primary breast cancer cells were isolated from breast cancer tissues derived from ten patients. Nuclear and cytoplasmic fractions were subjected to immunoblotting (IB) analysis (**a**). The expression of CD24 and CD44 were analysed using flow cytometry (**b**). Nuclear and cytoplasmic fractions of AURKA were normalized using histone H3 and β-actin, respectively. The nuclear fraction of c-Myc and hnRNP K were normalized using histone H3. The normalized AURKA, c-Myc, hnRNP K and the CD24^low^/CD44^high^ population derived from the same patient were used to compose a scatterplot and linear regression analysis performed (**c**). (**d**) Breast cancer tissues were subjected to IHC staining for indicated antibodies. Representative images were acquired with × 10 and × 40 objectives (upper panel). Scale bar, 50 μm. Pearson's *χ*^2^-test was used to analyse the correlation between nuclear AURKA and c-Myc/CD24 (lower panel). (**e**) IHC staining for AURKA and c-Myc was performed on breast cancer tissues. Kaplan–Meier analysis was performed and log-rank test used to make statistical comparisons. (**f**) During breast cancer development, AURKA is overexpressed and translocates to the nucleus, where the nuclear AURKA acts as a transactivating factor that binds and activates the *MYC* promoter through its interaction with hnRNP K. Through these mechanisms, nuclear AURKA enhances BCSC phenotype. Importantly, these processes are dependent on the nuclear localization of AURKA rather than its kinase activity. Traditional targeted therapies focus on the inhibition of kinase activity, which may be insufficient for suppressing the kinase-independent oncogenic actions. These new findings suggest that targeting the spatial deregulation of kinase could be a promising strategy for overcoming kinase inhibitor insensitivity.

To further substantiate the positive regulatory role of nuclear AURKA and hnRNP K in *MYC* expression and promotion of BCSC phenotype, we isolated primary cells from human breast cancer tissues. The purity of the enriched epithelial cells from these tissues was >85% in ten samples (Supplementary Fig. 7a). A correlation analysis was performed for the expression of nuclear/cytoplasmic AURKA, c-Myc, hnRNP K (Fig. 7a) and the ratio of the CD24^low^/CD44^high^ population (Fig. 7b). Nuclear but not cytoplasmic AURKA expression was positively correlated with the percentage of the CD24^low^/CD44^high^ population and the expression of nuclear c-Myc (Fig. 7c). We then performed IHC staining of AURKA, CD24, c-Myc and hnRNP K, to determine their correlations. The cutoff values used to distinguish between high and low expression were determined by a receiver operating characteristic plot (Supplementary Fig. 7b–d). Nuclear AURKA expression was positively correlated with c-Myc expression and negatively correlated with CD24 expression (Fig. 7d). A higher nuclear AURKA expression indicated a poorer prognosis for patients (Supplementary Fig. 7e). Nuclear AURKA^high^/c-Myc^high^ expression predicted an inferior overall survival when compared with AURKA^high^/c-Myc^low^, AURKA^low^/c-Myc^high^ or AURKA^low^/c-Myc^low^ expression (Fig. 7e). *MYC* is frequently amplified in various cancers^40^. To confirm the correlation between AURKA and *MYC*, we re-evaluated their correlations in samples with normal *MYC* copy number. We found that 53 of 59 samples showed normal *MYC* copy number. We then subjected these samples to IHC staining of AURKA and c-Myc, and studied 31 samples with both of IHC staining of AURKA and c-Myc. Nuclear AURKA expression was still positively correlated with c-Myc expression (Supplementary Fig. 7f). In summary, these clinical data support our findings that nuclear AURKA is important for the regulation of *MYC* expression and thus the oncogenic properties of BCSC.

## Discussion

AURKA is known for its function in cell cycle, where it acts in the cytoplasm during prophase of mitosis to organize the centrosomes. Here we establish a new molecular role for AURKA by demonstrating that nuclear AURKA promotes the expansion of BCSC. Mechanistically, nuclear AURKA acts as a transactivating factor to activate the expression of *MYC* from P1 promoter via its interaction with hnRNP K. The activation of *MYC* by AURKA is dependent on the nuclear localization of AURKA rather than its kinase activity. We further demonstrate that amino acids 333–383 are responsible for nuclear translocation of AURKA. Finally, we show that blocking AURKA nuclear localization sensitizes resistant BCSCs to AURKA kinase-targeted therapy.

Transcripts initiated from two tandem promoters, P1 and P2, account for the majority of *MYC* expression. In normal proliferating cells, the ratio of P1/P2 transcripts varies between 1:10 and 1:5 (ref. 36). In some cases, the relative utilization of P1 and P2 promoter is changed, inducing a higher ratio of P1 to P2 transcripts. In Burkitt's lymphoma, the *MYC* locus (8q24) is commonly juxtaposed through chromosomal translocation to one of the immunoglobulin loci IgH (14q32), Igκ (2p12) or Igλ (22q11)^41^. These translocations cause P1 to be more active than P2. In addition, the shift in *MYC* P1/P2 promoter utilization is also found during the cell cycle activation of lymphocytes^42^ and the postnatal cerebellar development^43^. Interestingly, we also found that the shift in *MYC* P1/P2 promoter utilization also occurs in BCSC (Fig. 2l and Supplementary Fig. 2n), suggesting that the relative utilization of *MYC* P1 and P2 could be a hallmark for BCSC. Our data indicate that AURKA binds to *MYC* promoter to induce a shift in *MYC* P1/P2 promoter utilization (Fig. 2j,n). The CT element locates at 100–150 bp 5′ of the P1 promoter and contains five, in part, imperfect repeats of the sequence 5′-CCCTCCCC-3′ (refs 44, 45). The integrity of CT element is essential for P1 promoter activation and is important for maintaining maximal P2 activity^45^. Various factors bind specifically to CT element, to modulate *MYC* promoter activity. For example, Sp1 binds to the duplex form^45^, hnRNP K to C-rich single strands^44^ and CNBP to G-rich single strands^46^. Our data show that mutation of CT element abolished AURKA-induced P1 promoter activation (Fig. 4d,g). Consistently, downregulation of hnRNP K suppressed the interaction between AURKA and CT element, and abolished the AURKA-induced shift in *MYC* P1/P2 promoter utilization (Fig. 4b,e).

Although we show that AURKA promotes *MYC* expression by acting as a transactivating factor in a kinase-independent manner, there are multiple routes through which AURKA could activate *MYC* expression. AURKA may interact with the WNT/β–catenin pathway. Overexpression of AURKA increases the phosphorylation of GSK-3β at Ser 9 and the nuclear β-catenin levels, which enhances β-catenin/TCF transcription activity and the transcription of its downstream target genes including *CCND1* and *MYC*^47^. p53 controls the expression of multiple cell cycle genes such as *MYC*^48^. AURKA may also regulate cell cycle activation or *MYC* expression by modulating p53 function^31^. Our previous study showed that inhibition of AURKA kinase activity by AKI603 induced the downregulation of c-Myc protein level^49^. We considered that the downregulation of c-Myc by Aurora kinase inhibitor is caused by the secondary effects of growth arrest. To exclude the impact of growth arrest, we conducted kinase activity inhibition in cells synchronized in M phase, to evaluate c-Myc expression. Our data showed that the expression of c-Myc is not reduced by Aurora kinase inhibitor VX-680 in synchronized cells, whereas the expression of c-Myc was reduced in unsynchronized cells after VX-680 treatment (Supplementary Fig. 7g).

AURKA is critical for mitosis because of its role in regulating centrosome function^50^ and microtubule dynamics^51^. This AURKA function is probably dependent on its cytoplasmic activity due to the cytoplasmic localization of its substrates in the centrosome and microtubule. Recent studies show that mitotic function of AURKA is critical for regulating stem cell function. AURKA participates in the control of cell fate determination through modulating the asymmetric distribution of cell fate determinant^52^ and the orientation of the mitotic spindle^53^. Consistent with previous study^49^, our data show that inhibition of AURKA kinase activity reduces the BCSC population (Fig. 6d). The suppression of BCSC through inhibition of AURKA kinase activity might be mainly due to impairing the function of cytoplasmic AURKA. Accordingly, we found that inhibition of AURKA kinase activity greatly reduced the CD24^low^/CD44^high^ BCSC population in cells expressing NES-fused AURKA, but not in cells expressing NLS-fused AURKA (Supplementary Fig. 6d,e). These results suggest that the oncogenic function of cytoplasmic AURKA relies predominantly on its kinase function, and that nuclear AURKA possesses kinase-independent activity, which is not repressed by the AURKA kinase inhibitor.

Although inhibiting the kinase activity of AURKA induced anti-BCSC effects, a significant proportion of BCSC is refractory to the kinase activity inhibition (Fig. 6d). Targeting the transactivating function by preventing AURKA translocation to the nucleus significantly enhanced the ability of AURKA kinase to inhibit the expansion of BCSC (Fig. 6e,f). Previous studies have identified several mutations of AURKA in cancer, such as F31I and V57I^54^. Interestingly, the low-kinase-activity AURKA mutant (F31/I57) can induce higher levels of genomic instability and increase oesophageal cancer risk compared with other kinase-dependent mutations such as (I31/V57)^54^. These findings are consistent with our results that indicated AURKA may promote cancer development via mechanisms that are independent of its kinase activity. In addition, other studies have indicated that AURKA stabilizes N-Myc independently of its kinase activity^17^, and that N-Myc can be destabilized using the Aurora kinase inhibitor, MLN8237, which disrupts the interaction between N-Myc and AURKA^55^. Although these previous studies suggest a strategy to overcome drug resistance to kinase-activity inhibition by disrupting protein–protein interactions, our new findings strongly support an alternative therapeutic avenue by blocking the nuclear localization of AURKA.

In summary, our study demonstrates that the spatial deregulation of AURKA confers a previously unknown oncogenic function to AURKA that is not sensitive to conventional kinase inhibitory strategies and contributes to drug resistance. These new findings further advance our understanding of the mechanism of drug insensitivity and provide a novel strategy to overcome drug resistance (Fig. 7f).

## Methods

### Chemicals and antibodies

VX-680, MLN8237 and Aurora A Inhibitor I were purchased from Selleck Chemicals. OHT, Nocodazole and puromycin were purchased from Sigma-Aldrich. The following primary antibodies were used: AURKA (rabbit; Millipore, 1:4,000, catalogue number 07–648), AURKA (mouse; Abcam, 1:4,000, catalogue number ab13824), phospho-AURKA (T288; Cell Signaling, 1:1,000, catalogue number 3079), haemagglutinin (HA) tag (Sigma-Aldrich, 1:4,000, catalogue number SAB4300603), FLAG tag (Sigma-Aldrich, 1:4,000, catalogue number F1804), glyceraldehyde 3-phosphate dehydrogense (Thermo Fisher Scientific, 1:4,000, catalogue number AM4300), β-actin (Cell Signaling, 1:4,000, catalogue number 4967), c-Myc (Santa Cruz, 1:2,000, catalogue number sc-764), Histone H3 (Cell Signaling, 1:2,000, catalogue number 4499), hnRNP K (Cell Signaling, 1:4,000, catalogue number 4675), enhanced green fluorescent protein (Cell Signaling, 1:4,000, catalogue number 2555), GST (Cell Signaling, 1:4,000, catalogue number 2624s), Erk1/2 (Cell Signaling, 1:2,000, catalogue number 9102), phospho-Erk1/2 (Thr202/Tyr204; Cell Signaling, 1:2,000, catalogue number 9101), Ras (Cell Signaling, 1:1,000, catalogue number 3965), phospho-p53 (Ser315; Cell Signaling, 1:1,000, catalogue number 2528), p53 (Cell Signaling, 1:1,000, catalogue number 2524s), phospho-Histone H3 (Ser10; Cell Signaling, 1:1,000, catalogue number 9701), Histone H3 (Cell Signaling, 1:1,000, catalogue number 9715).

### Cell lines

Human breast cancer cell lines (BT549, MDA-MB-231, MCF-10A and Sk-br-3), mouse embryonic fibroblasts and 293T cells were obtained from the American Type Culture Collection (ATCC). These cell lines were authenticated at ATCC before purchase by their standard short tandem repeat DNA-typing methodology. SUM149 was kindly provided by Professor Zhimin Shao (Department of Medical Oncology, Cancer Hospital of Fudan University, Shanghai Medical College, China). SUM149 was authenticated by the standard short tandem repeat DNA-typing methodology. Each cell line was cultured in its standard medium as recommended by ATCC. The SUM149 cells were cultured in F12 (Gibco) supplemented with 5% fetal bovine serum (Hyclone), 5 μg ml^−1^ insulin (Sigma-Aldrich) and 1 μg ml^−1^ hydrocortisone (Sigma-Aldrich). All cells were maintained at 37 °C in a humidified 5% CO_2_ atmosphere.

### Human breast cancer specimens and primary cell isolation

Following informed consent and in accordance with the guidelines of the Institutional Review Boards, breast cancer specimens were obtained from patients undergoing surgery at the Third Affiliated Hospital of Sun Yat-Sen University, China. Primary breast cancer isolation was performed. Ten breast lesions, following histological diagnostic assessment and sampling by pathologists, were mechanically and enzymatically dissociated to yield clumps of epithelial cells, termed organoids, by incubating at 37 °C for 2 h in a 1:1 solution of collagenase I (3 mg ml^−1^)/hyaluronidase (100 U ml^−1^) (Sigma-Aldrich). After filtration through a 40-μm pore filter and washing with PBS, the organoids from tumour tissues were dissociated into single cells using trypsin, for subsequent cytoplasmic and nuclear protein isolation and CD24/CD44 analysis.

### RNA interference

The target sequences of AURKA siRNAs (GenePharma) were as follows: 1, 5′-ATGCCCTGTCTTACTGTCA-3′; 2, 5′-GGCAACCAGTGTACCTCAT-3′; and 3, 5′-ATTCTTCCCAGCGCGTTCC-3′. The target sequence of hnRNP K siRNA (GenePharma) was 5′-AATATTAAGGCTCTCCGTACA-3′ and the negative control siRNA sequence was 5′-TTCTCCGAACGTGTCACGT-3′. The shAURKA (TRC number: TRCN0000000655) and sh control (SHC002) plasmids were purchased from Sigma-Aldrich. The shRNA sequences against hnRNP K (sense: 5′-CGCGTCCCCGTTATTGTTGGTGGTTTAATTCAAGAGATTAAACCACCAACAATAACTTTTTGGAAAT-3′ and antisense: 5′-CGATTTCCAAAAAGTTATTGTTGGTGGTTTAATCTCTTGAATTAAACCACCAACAATAACGGGGA-3′) were cloned into pLVTHM (Addgene).

### Immunoprecipitation

The cells were lysed in RIPA buffer containing 50 mM Tris pH 7.4, 150 mM NaCl, 1% NP-40, 0.5% sodium deoxycholate, 0.1% SDS, 1 mM phenylmethyl sulfonyl fluoride, complete protease and phosphatase inhibitor cocktail (Roche). Co-IP was performed by using 1 μg of antibodies and 200–500 μg of lysate for exogenous proteins or 2–5 mg of lysate for endogenous proteins. Clean-Blot IP Detection Reagents (Thermo Scientific) were used to recognize the native primary antibody.

### GST pull-down assays

293T cells overexpressing HA-tagged AURKA or hnRNP K were lysed in RIPA buffer. Equal amounts (5 μg) of the GST fusion proteins were incubated in the lysate for 1 h at 4 °C. Glutathione–agarose beads (Sigma-Aldrich) were incubated in the lysate for 1 h at 4 °C. Next, the beads were washed three times with lysis buffer and eluted by boiling in SDS sample buffer.

### Immunoblotting

Cells were washed twice with cold PBS. Next, cells were lysed in RIPA buffer containing 50 mM Tris pH 7.4, 150 mM NaCl, 1% NP-40, 0.5% sodium deoxycholate, 0.1% SDS, 1 mM phenylmethyl sulfonyl fluoride, complete protease and phosphatase inhibitor cocktail (Roche) for 5 min, and were collected by scraping. Lysates were cleared by centrifuging at 12,000 r.p.m. for 10 min. Protein was quantified by Bradford Assay. Sample proteins were separated by SDS–PAGE gel electrophoresis. Next, proteins were transferred from gel to a nitrocellulose membrane (Millipore). After that, membranes were blocked in 5% BSA TBST for 30 min at room temperature (RT) and then incubated overnight with indicated primary antibodies at 4 °C. After that, membranes were washed three times and incubated with peroxidase-conjugated secondary antibodies (1:5,000, Millipore) and detected with enhanced chemiluminescence (Millipore) on X-ray films (Kodak). Uncropped scans of all the blots are shown in Supplementary Fig. 8.

### Animal studies

Animal care was performed with approval of the Institutional Animal Care and Use Committee at Sun Yat-Sen University Cancer Center.

For limiting dilution assays (Fig. 4l), cells sorted according to CD24 expression were serially diluted from 1 × 10^5^ to 1 × 10^2^ cells per 100 μl in PBS containing 50% Matrigel (BD Biosciences), followed by subcutaneous injection into nonobese diabetic/severe combined immunodeficiency mice. Tumour formation was examined once every 4 days and the total observation period was 16 weeks.

For the animal studies presented in Fig. 6b, the AER fusion gene and the puromycin-resistance gene were expressed via lentivirus-mediated gene transfer. Endogenous AURKA was knocked down via lentivirus delivery of shRNA (shAURKA); RFP was also expressed by the same lentivirus vector. MDA-MB-231 cells expressing the AER fusion protein and AURKA shRNA were initially selected using 1 μg ml^−1^ puromycin for 48 h, followed by FACS sorting according to the RFP signal intensity. Female nude mice (4–6 weeks of age) were injected subcutaneously into the right flank with 2 × 10^6^ cells containing 30% Matrigel. At the time of cell injection, OHT (100 μg per 100 μl in peanut oil) was delivered via intraperitoneal injection every other day. When the tumours reached ∼150 mm^3^ in volume, the mice were randomly separated into four groups and treated with OHT+polyethylene glycol 300 (PEG), oil (OHT removed)+PEG, OHT+VX-680 or oil (OHT removed)+VX-680, respectively, via intraperitoneal injection. VX-680 was prepared in a vehicle of 50% PEG in PBS and was administered at a dose of 40 mg kg^−1^. The tumour volumes were determined using calipers to measure the longest (length) and shortest (diameter) every other day and were calculated according to the standard formula (length × diameter^2^ × 0.52). Next, the tumour samples were harvested from killed mice and were processed for single-cell suspension preparation and IHC analysis.

### Flow cytometric analysis

Anti-Human/Mouse CD44 FITC (eBioscience) and Anti-Human CD24 PerCP-eFluor 710 (eBioscience), Anti-Human CD24 PE (eBioscience) and Anti-Human CD326 (EpCAM) PerCP-eFluor 710 (eBioscience) were used for flow cytometric analysis. Cells were harvested by trypsinization and washed once in cold PBS. Next, cells were counted and resuspended in cold PBS at a concentration of 1 × 10^6^ cells per 100 μl. Staining was performed by incubating 100 μl cells with 5 μl antibody on ice for 30 min.

### Cell sorting

For flow cytometric cell sorting, the cells were dissociated into single-cell suspensions, followed by labelling with Anti-Human CD24 PerCP-eFluor 710 (eBioscience). Next, the labelled cells or fluorescent protein-expressed cells were physically sorted by using a FACS flow cytometer (Beckman). The enrichment was confirmed by flow cytometric analysis.

For side population sorting, cells were resuspended in 37 °C DMEM medium containing 2% fetal bovine serum at a density of 1 × 10^6^ cells per ml. Cells were incubated with 5 μg ml^−1^ Hoechst 33342 (Sigma) in the presence or absence of 50 μM verapamil (Sigma) for 1.5 h at 37 °C in darkness with intermittent shaking. Next, cells were washed twice with PBS and resuspended in PBS for flow cytometric sorting. Cell sorting was performed on a FACS flow cytometer (Beckman).

### Lentivirus preparation and transfection

Lentivirus was produced in 293T cells using the second-generation packaging system plasmids psPAX2 (Addgene) and pMD2.G (Addgene). One 10-cm culture dish containing 5 × 10^6^ 293T cells was transfected using Lipofectamine 2000 (Life) with 12 μg lentiviral vector, 9 μg psPAX2 and 3 μg pMD2.G. Supernatants were collected every 24 h between 24 and 72 h after transfection, pulled together and concentrated via ultracentrifugation, and the viral titre was determined by serial dilutions. The multiplicity of infection during transfection was 5.

### Mammosphere culture

Single-cell suspension was obtained by trypsinization and sieving through a 40-μm sieve and single cellularity was examined via microscopy. Single cells were plated at a density 1 × 10^3^ cells per ml in ultralow attachment six-well plates (Corning). The cells were cultured in medium consisting of DMEM/F12 (Gibco), supplemented with B27 (Life Technologies), 20 ng ml^−1^ epidermal growth factor (Sigma-Aldrich), 20 ng ml^−1^ basic fibroblast growth factor (BD Biosciences) and 4 μg ml^−1^ heparin (Sigma-Aldrich) for 9–12 days. For evaluation, the mammospheres were photographed under an inverted microscope (× 10objective, Olympus). The mammosphere diameters were analysed using Image pro plus 6.0 software (Media Cybernetics).

### Cytoplasmic/nuclear protein extraction

Cytoplasmic extracts were prepared by resuspending the cell pellets in Buffer I, which contained 25 mM HEPES pH 7.9, 5 mM KCl, 0.5 mM MgCl_2_ and 1 mM dithiothreitol (DTT) for 5 min. Next, an equal volume of Buffer II containing 25 mM HEPES pH 7.9, 5 mM KCl, 0.5 mM MgCl_2_, 1 mM DTT and 0.4% (v/v) NP40 supplemented with protease and phosphatase inhibitors was added and the samples were incubated with rotation at 4 °C for 15 min. The lysates were centrifuged for 5 min at 4 °C at 2,500 r.p.m. in a microfuge. Next, the supernatants were transferred to new Eppendorf tubes. The pellets were rinsed once with Buffer II and the wash transferred to cytoplasmic protein. The lysates were centrifuged again for 5 min at 4 °C at 10,000*g*, to remove the residual nuclei. Next, the supernatants were transferred to new Eppendorf tubes.

To obtain nuclear extracts, the pellets from the cytoplasmic extraction were treated with Buffer III containing 25 mM HEPES pH 7.9, 400 mM NaCl, 10% sucrose or dextrose, 0.05% NP-40 and 1 mM DTT supplemented with protease and phosphatase inhibitors. After rotating for 1 h at 4 °C, the lysates were centrifuged for 10 min at 4 °C, at 10,000*g*. Supernatants after this spin contained the nuclear protein preparation.

### Dual-luciferase reporter assays

Luciferase activity was measured using the Dual-Luciferase Reporter Assay system (Promega) according to the manufacturer's instructions. Growth media were removed and cells were washed with PBS. Passive lysis buffer (Promega) 500 μl per well was added with gentle rocking for 15 min at RT. Ten microlitres of lysate were transferred in black 96-well plate (Thermo). Firefly and *Renilla* luciferase activity were assayed sequentially to the cell lysate in each well. For each luminescence reading, there would be a 2-s pre-measurement delay after injector dispensing assay reagents into each well, followed by a 10-s measurement time. Transcriptional activity was calculated as the ratio of firefly luciferase activity (reporter) to *Renilla* luciferase activity (control).

### ChIP and re-ChIP assays

ChIP was performed using ChIP-IT Express Chromatin Immunoprecipitation Kits (Active Motif) according to the manufacturer's protocol. Briefly, 1 × 10^7^ cells, which were treated with the conditions described in the figure legends or Supplementary Figurelegends, were fixed with 1% formaldehyde for 10 min at RT. Next, the cells were washed twice with PBS at 4 °C, collected and resuspended in ice-cold lysis buffer and lysed on ice for 30 min. The cells were homogenized on ice, to aid in nuclei release. Cells were sonicated five times for 5 s at 50% of maximal power (Fisher Sonic Dismembrator). The chromatin (25 μg) was immunoprecipitated for 12 h with 2 μg of specific antibodies against AURKA (Millipore), hnRNP K (Cell Signaling) or IgG (rabbit, Santa Cruz) and Protein G magnetic beads (25 μl). Beads were then washed sequentially for 5 min with the following buffers: ChIP Buffer I for one time and ChIP Buffer II for two times. The immune complexes were eluted with 50 μl elution buffer AM2. The supernatants were reverse cross-linked by heating at 65 °C for 12 h, treated with 1 μl RNaseA at 37 for 15 min and digested with 2 μl proteinase K at 37 °C for 1 h. DNA was obtained by phenol and phenol/chloroform extractions. The human *MYC* promoter-specific primers used for PCR were 5′-GTCAAACAGTACTGCTACGG-3′ (forward) and 5′-TTCTTTTCCCCCACGCCCTC-3′ (reverse). The primer targeting a *MYC* gene exon was used as a negative control in these experiments. *MYC* exon-specific primers were 5′-GGATATCTGGAAGAAATTCGAGC-3′ (forward) and 5′-GATGAAGGTCTCGTCGTCCG-3′ (reverse). Re-ChIP assays were performed as described using the Re-ChIP-IT kit (Active Motif). Briefly, the precipitated chromatin from the first ChIP reaction was eluted by 100 μl diluted Re-ChIP-IT elution buffer at RT for 30 min. Next, the eluted precipitate was desalted by the desalting column provided in the kit. The second ChIP was performed with 25 μl Protein G magnetic beads, 90 μl desalted chromatin and 2 μg second antibody. Next, the second precipitate was washed, eluted and reverse cross-linked as in the first ChIP. DNA was obtained by phenol and phenol/chloroform extractions, and subjected to real-time PCR evaluation.

### IHC and scoring

Antigen retrieval was performed by heating the sample in EDTA buffer (pH 8.0) in a microwave oven for 15 min. The slides were stained for 25 min at RT. EnVision Detection Systems (Dako) was used to detect antigen expression.

The IHC staining was quantified as the H-score, which has been validated for breast cancer IHC staining^56^. The images were acquired using a Nuance EX multispectral imaging system (PerkinElmer) under identical conditions; two fields of each sample in the tissue microarray were acquired using a × 20 objective. Scoring was performed using inForm software (PerkinElmer). Typical images corresponding to negative (four images, scored as ‘0'), weak (four images, scored as ‘1'), intermediate (four images, scored as ‘2') and strong (four images, scored as ‘3') brown staining were selected by two independent experienced pathologist for software training. During training, an algorithm, which helps the software to distinguish between the cancer and non-cancer tissues, and distinguish between the nuclear and cytoplasmic regions, and sets the thresholds for 0–3 staining, was optimized. Next, the optimized algorithm was used to perform scoring of the other samples. The H-score (between 0 and 300) for each sample was calculated in the following way: (% of cells stained at intensity 1 × 1)+(% of cells stained at intensity 2 × 2)+(% of cells stained at intensity 3 × 3). The receiver operating characteristic curve analysis was used to select the cutoff point for each variable.

### Semi-quantitative and quantitative PCR

Total RNA was extracted using TRIzol reagent (Life Technologies) according to the manufacturer's protocol. Complementary DNA was synthesized by using M-MLV Reverse Transcriptase (Life Technologies) according to the manufacturer's instructions. Semi-quantitative PCR was performed by using Premix Taq (Takara) according to the manufacturer's instructions. The primers used in semi-quantitative PCR were as follows: AURKA, sense-5′-GGAGAGCTTAAAATTGCAGATTTTG-3′, antisense-5′-GCTCCAGAGATCCACCTTCTCAT-3′; MYC, sense-5′-TCAAGAGGCGAACACACAAC-3′, antisense-5′-GGCCTTTTCATTGTTTTCCA; glyceraldehyde 3-phosphate dehydrogense, sense-5′-TGCCAAATATGATGACATCAAGAA-3′, antisense-5′-GGAGTGGGTGTCGCTGTTG-3′. The primers used in quantitative PCR were shown in Supplementary Table 4.

### Fluorescence resonance energy transfer

293T cells were transiently transfected with AURKA in the pEYFP-C1 vector (AURKA-EYFP) and hnRNP K in the pECFP-C1 vector (hnRNP K-ECFP) using Lipofectamine 2000 (Life) according to the manufacturer's protocol. Fluorescence imaging was performed at RT 48 h after transfection. Before fluorescence recording, the culture media was replaced with a solution containing 130 mM NaCl, 5 mM MgCl_2_, 2 mM CaCl_2_, 5 mM HEPES and 1 mM EGTA (pH 7.4).

The FRET signals were determined as sensitized emission using a Leica Advanced Widefield System AF7000 equipped with three FRET sets and a × 63 glycerol immersion objective (numerical aperture 1.30). The FRET sets included two external filter wheels, which facilitate high-speed FRET acquisition for each fluorophore excitation and emission wavelength, and one matching filter cube equipped with a dichroic mirror (440:520). The excitation light was generated using a 100-mW mercury lamp. The light intensity was controlled using a series of neutral density filters installed in the external filter wheels. Fluorescence emission was detected using a Hamamatsu electron multiplying charge-coupled device. For FRET acquisition, three channels were defined as follows: channel 1 for donor only (donor excitation with donor emission); channel 2 for FRET (donor excitation with acceptor emission); and channel 3 for acceptor only (acceptor excitation with acceptor emission for yellow fluorescent protein (YFP) cell imaging). Straightforward correction was performed using both a donor-only reference and an acceptor-only reference. The background value was determined from a region in the recorded image that did not contain any cells. The FRET efficiency was calculated as the enhanced YFP emission due to energy transfer (*E*=acceptor^sensitized emission^/acceptor^direct emission^).

### Molecular dynamics simulation

To obtain the protein structure of AURKA, we predicted the structures of AURKA (1–125) and AURKA (390–403) using an online I-TASSER server. Next, the predicted structure was assembled onto the existing crystal structure of residues 126–389 (PDB:2J4F)^57^ to build the complete structure. Similarly, we obtained the structure of the hnRNP K KI domain (residues 240–337). The conformation of the complexes formed between AURKA and hnRNPK were predicted using Rossetta.

All simulations were carried out with Gromacs4.05 programme^58^. The Gromos96(53a)^59^ force field was used for the proteins and the TIP3P6 model for the waters. In preparation for molecular dynamics simulation, the protein was solvated using water pre-equilibrated at the appropriate temperature such that the water box extended 10 Å from the protein. Twenty-seven Cl^−^ anions were added to neutralize the system. The staring structure was first subjected to 1,000 steps of steepest descent minimization and then heated to the 300 K with initial velocities assigned form a Maxwellian distribution within 10 ns. In the following simulation, bonds involving hydrogens were constrained with Lincs method^60^, thus permiting an integration time step of 2 fs. The non-bonded interaction was cut off at 14 Å, whereas the electrostatic interaction beyond that was treated with particle mesh Ewald method^61^. The charge-based neighbour list was updated every ten steps. The system was maintained at 300 K and 1 bar using Berendsen coupling method^62^ with a coupling frequency of every 0.5 and 0.1 ps, respectively. To ensure that the system visits all possible minima, a production simulation of 2 μs was performed and the trajectory was output every 100 ps for further analysis.

We obtained 2,000 conformations from the final 1.0 μs after the equilibration and performed analysis based on these snapshots. The interaction residues between these two proteins for all the 2,000 conformations were determined by the following criterion. If the shortest distance between any heavy atoms from two residues belonging to two proteins was shorter than 4 Å, then these two residues from two proteins were considered to be the interaction residue pair. By counting the pair and its corresponding interaction number, we saved the residue pairs if the interaction occurs more than 1,000 times. These saved residue pairs were defined as the interface residues between these two proteins.

### Bioinformatics analysis

For analysis using the Gene Expression Omnibus data (accession code: GSE23541)^63^, the probes with detection *P*-values <0.01 in all samples were excluded from the data set before analyses. If multiple probes corresponded to the same gene, the expression values of these probes were averaged. GSEAs^64^ were performed using GSEA v2.0.13 software (http://www.broad.mit.edu/gsea) with 1,000 data permutations. The gene expression signature were acquired from public research^65^,^66^,^67^ and GSEA website. Statistical significance was evaluated by means of false discovery rate (≤0.25) and *P*-value calculations (*P*<0.05).

For STRING (http://string-db.org/) analysis, differential expression genes were subjected for analysing with default analysing parameters, except confidence≥0.7. The raw data for STRING analysis were uploaded to NCBI (accession code: GSE57931).

### SILAC for identifying the interacting candidates of AURKA

Cells stably expressing AURKA-Flag were cultured in SILAC labelling medium containing 107 mg l^−1^ D3-leucine (heavy). The control cells (expressing Flag) were cultured in SILAC labelling medium containing 105 mg l^−1^ D0-leucine (light). After eight passages, cells were lysed in RIPA. A 1:1 ratio of proteins from heavy and light labelled cells was combined and immunoprecipitated by using anti-Flag M2 agarose beads. The beads were washed in RIPA and eluted with buffer containing 200 mM Tris-HCl pH 8.0, 0.2 mg ml^−1^ Flag peptide (Sigma). The eluted proteins were separated via SDS–PAGE and in-gel trypsin digestion was performed. Liquid chromatography tandem mass spectrometry was performed on a LTQ-Orbitrap instrument (Thermo Electron Corporation). The data were analysed using the IPI databases for human version 3.35 with BioWorks 3.2 software (Thermo Electron Corporation). The peptide/protein ratios were calculated using the SILAC function module of the BioWorks software package with the following settings: target residue at leucine, mass at 3.0188, mass tolerance at 0.01 Da, minimum threshold at 10, no smoothing and run the calculation based on area.

### S1 nuclease protection assay

The DNA probes used to detect *MYC* P1 and P2 transcripts were prepared using PCR DIG Probe Synthesis Kit (Roche). The probe for *MYC* −340∼+510 (the site of P1 initiation designated +1) was used in Fig. 2l–o, Fig. 4e and Supplementary Fig. 2n. The probe corresponding to exon2 of *B2M* and its upstream 362 bp intron was used as control and was also used in Fig. 2l–o, Fig. 4e and Supplementary Fig. 2n. The probe consisted of *MYC* (−226∼+211) and firefly luciferase 1–400 bp was used in Fig. 4f. The hybridization mixture contained 25 ng ml^−1^ of probe, 15 μg of total cellular RNA, 80% formamide, 400 mM NaCl, 40 mM PIPES (pH 6.4), 1 mM EDTA. The hybridization temperatures for the *MYC* probe and *B2M* were 58 °C and 52 °C, respectively. The hybridization temperature for the probe of chimeric gene was 58 °C. The hybridzation was terminated by adding 750 U S1 nuclease (Life) and digestion carried out at 37 °C for 1 h.

### Fluorescence *in situ* hybridization

Formalin-fixed paraffin-embedded breast cancer tissue microarrays were pretreated (Dewax/Proteolysis) by the ZytoLight FISH-Tissue Implementation Kit (Zytovision) according to the manufacturer's protocol. The copy number of *MYC* were detected by ZytoLight SPEC CMYC/CEN 8 Dual Color Probe (Zytovision). Specifically, denaturation was performed at 75 °C for 10 min followed by hybridization overnight at 37 °C. 4,6-Diamidino-2-phenylindole /Antifade Solution was applied after hybridization according to the protocol. Image acquisition and analysis of slides were performed in the confocal microscope (OLYMPUS FV1000). The copy number of CMYC/CEN 8 was determined according to the manufacturer's instructions by green/red spots, respectively.

### Plasmids

pLVX-HA was constructed by replacing *DsRed* gene in pLVX-DsRed-N1-Monomer (Clontech) with HA sequence. WT AURKA was amplified from MDA-MB-231 cells and insert into pLVX-HA to construct pLVX-AurA(WT)-HA. pLVX-AurA(KA)-HA and pLVX-AurA(KD)-HA were generated by site-directed mutagenesis using pLVX-AurA(WT)-HA. NLS or NES were fused to the carboxy and amino terminus of AURKA by PCR, then were inserted into pLVX-HA to construct pLVX-NLS-AURKA and pLVX-NES-AURKA. AURKA (1–383 or 1–333) were inserted into pLVX-HA to construct pLVX-1–383-HA or pLVX-1–333-HA.

HA-tagged ER was amplified from pBabe-puro-myc-ER (Addgene) and used to displace *DsRed* gene in pLVX-DsRed-N1-Monomer to construct pLVX-HA-ER. C terminus NES was added to WT, KA and KD AURKA by PCR and AURKA-NES was inserted into pLVX-HA-ER to produce AURKA-ER (AER). NLS-fused 1–333 of AURKA were inserted into pLVX-HA-ER to construct pLVX-HA-ER-NLS-1–333.

GAL4DBD were cloned from pM (Clontech) and inserted into pLVX-HA to construct pLVX-GAL4DBD-HA. WT, KA and KD AURKA were inserted into pLVX-GAL4-DBD-HA to construct pLVX-Gal4DBD-HA-AurA(WT), pLVX-Gal4DBD-HA-AurA(KA) and pLVX-Gal4DBD-HA-AurA(KD).

Promoters of *MYC*(−2,269/+516), *MYC*(−1,012/+516), *MYC*(−352/+516) and *MYC*(−104/+516) were amplified from MDA-MB-231 genomic DNA and inserted into pGL3-Basic (Clontech) to construct pGL3-Myc, pGL3-Myc(−1,012/+516), pGL3-Myc(−352/+516) and pGL3-Myc(−104/+516). The CT element was mutated by site-directed mutagenesis. *MYC*(−226/+211) was inserted in pGL3-Basic to construct chimeric gene. CT element was mutated by site-directed mutagenesis. *MYC*(−2,269/+516) was inserted into this plasmid to replace the cytomegalovirus (CMV) promoter of pLVX-DsRed-N1-Monomer. pRL-TK was purchased from Clontech. The GAL4DBD reporter, L8G5-Luc, was kindly provided by Professor Eric Lam (Imperial College London).

hnRNP K was amplified from MDA-MB-231 cells and inserted into pCMV-HA (Genechem) or pCMV-c-Flag (Beyotime) to construct pCMV-HA-hnRNPK or pCMV-hnRNPK-c-Flag, respectively. pCMV-hnRNPK(SA)-c-Flag and pCMV-hnRNPK(SD)-c-Flag were generated by site-directed mutagenesis. hnRNP A1 was amplified from MDA-MB-231 cells and inserted into pCMV-c-Flag to construct pCMV-hnRNPA1-c-Flag.

GST was amplified from pGEX-6P-3 (GE Healthcare) and replaced *DsRed* gene in pLVX-DsRed-N1-Monomer to generate pLVX-GST. Truncated form of AURKA and hnRNP K were inserted into pLVX-GST. Enhanced green fluorescent protein-fused full-length and truncated AURKA were purchased from GenePharma. NLS deletion mutant of hnRNP K was purchased from GenePharma.

### Statistical analysis

The statistical analysis was performed using SPSS version 16.0 (SPSS Inc.). The Kaplan–Meier method was used to predict survival. The log-rank test was used to make statistical comparisons. Pearson's correlation test was used to examine the correlation between IHC marker stainings. The Kruskal–Wallis test followed by Dunn's multiple comparison test were used to make a statistical comparison regarding mammosphere size distribution. The unpaired Student's *t*-test was used to compare two groups. Analysis of variance followed by the least significant difference test was used for multiple group comparisons. Statistical significance of tumour volumes was calculated via analysis of variance with Dunnett's multiple comparison tests. The level of significance was set at *P*<0.05.

## Additional information

**Accession codes:** GSE57931.

**How to cite this article:** Zheng, F. *et al.* Nuclear AURKA acquires kinase-independent transactivating function to enhance breast cancer stem cell phenotype. *Nat. Commun.* 7:10180 doi: 10.1038/ncomms10180 (2016).

## Supplementary Material

Supplementary Information Supplementary Figures 1-8, Supplementary Tables 1-4 and Supplementary Methods
