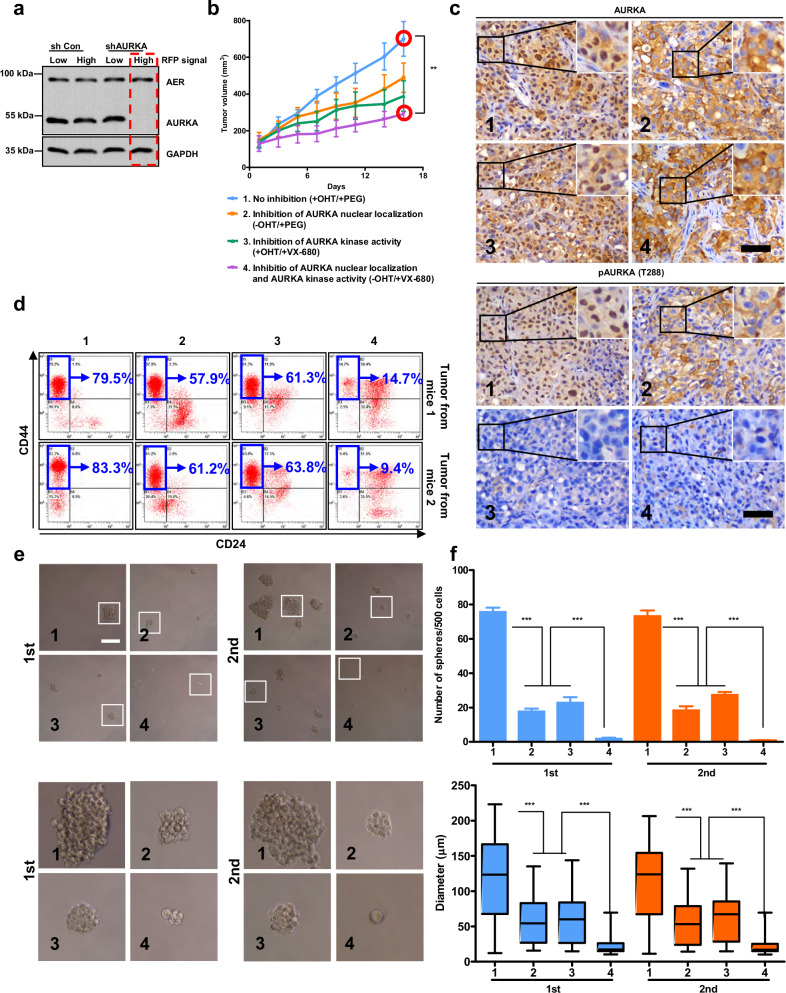# Author Correction: Nuclear AURKA acquires kinase-independent transactivating function to enhance breast cancer stem cell phenotype

**DOI:** 10.1038/s41467-026-69300-8

**Published:** 2026-02-20

**Authors:** Feimeng Zheng, Caifeng Yue, Guohui Li, Bin He, Wei Cheng, Xi Wang, Min Yan, Zijie Long, Wanshou Qiu, Zhongyu Yuan, Jie Xu, Bing Liu, Qian Shi, Eric W.-F. Lam, Mien-Chie Hung, Quentin Liu

**Affiliations:** 1 Sun Yat-Sen University Cancer Center, State Key Laboratory of Oncology in South China, Collaborative Innovation Center for Cancer Medicine, Guangzhou; Institute of Cancer Stem Cell, Dalian Medical University, 9 West Section, Lvshun South Road, Dalian, 116044 China; 2 Department of Laboratory Medicine, The First Affiliated Hospital, Sun Yat-Sen University, 58 Zhongshan Road II, Guangzhou, 510080 China; 3 State Key Laboratory of Molecular Reaction Dynamics, Dalian Institute of Chemical Physics, Chinese Academy of Sciences, 457 Zhongshan Road, Dalian, 116023 China; 4 The Third Affiliated Hospital, Sun Yat-Sen University, 600 Tianhe Road, Guangzhou, 510630 China; 5 Cancer Hospital/Cancer Institute, College of Life Sciences and Institutes of Biomedical Sciences, Fudan University, 220 Handan Road, Shanghai, 200433 China; 6 Department of Surgery and Cancer, Imperial College London, Du Cane Road, London, W12 0NN UK; 7 Department of Molecular and Cellular Oncology, The University of Texas MD Anderson Cancer Center, 1515 Holcombe Boulevard, Houston, Texas 77030 USA; 8 Center for Molecular Medicine and Graduate Institute of Cancer Biology, China Medical University, 6 HSUEH-SHIH Road, Taichung, 40402 Taiwan

Correction to: *Nature Communications* 10.1038/ncomms10180, published online 19 January 2016

In the version of the article initially published, in both “1st” panels in Fig. 6e, the images labelled “1” were incorrect due to an error in figure preparation (they were sourced from a different but methodologically similar mammosphere experiment done on the same day). The corrected Fig. 6 is shown below. This notice serves to amend the error, and the conclusions of the article remain unchanged.

Corrected Fig. 6