# Supplementary material for: Nuclear AURKA acquires kinase-independent transactivating function to enhance breast cancer stem cell phenotype
Source: Nat Commun. 2016 Jan 19;7:10180. doi: 10.1038/ncomms10180 (PMC4735655; doi:10.1038/ncomms10180)
Supplement: Supplementary Information — Supplementary Figures 1-8, Supplementary Tables 1-4 and Supplementary Methods [file ncomms10180-s1.pdf]

Supplementary Figure and Figure Legend

Supplementary Fig. 1

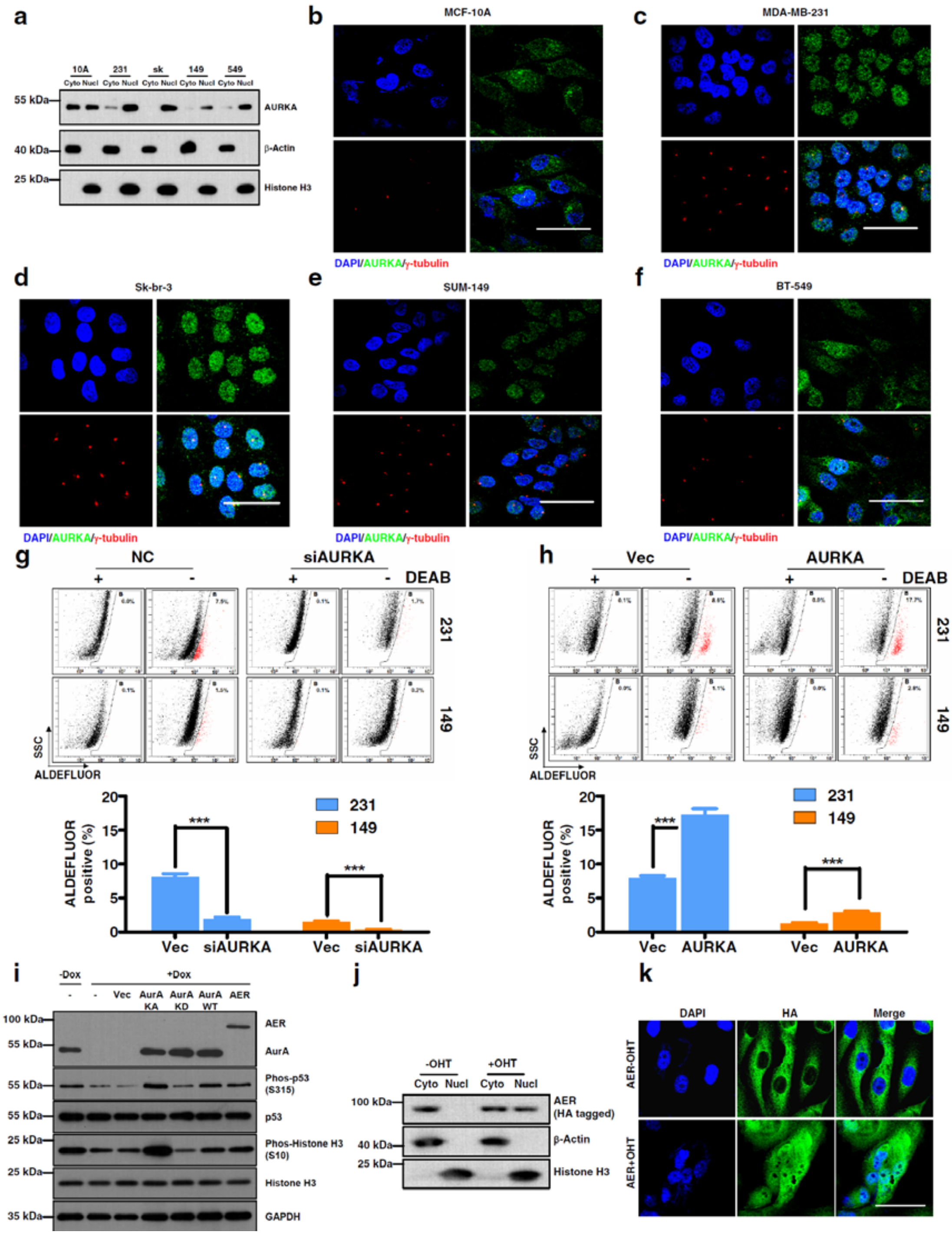

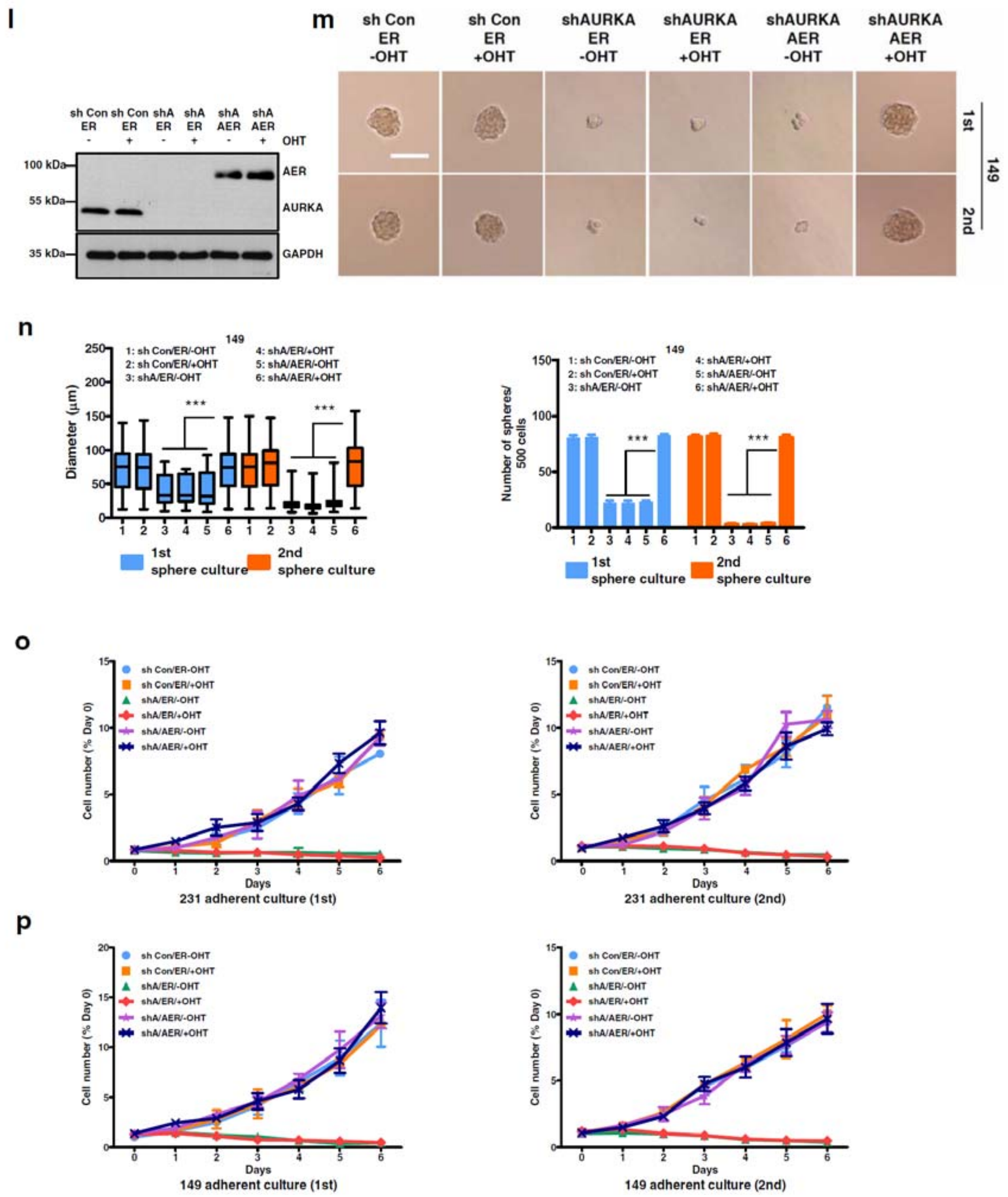

Supplementary Fig. 1 Related to Fig. 1

(a) MCF-10A (10A), MDA-MB-231 (231), Sk-br-3 (sk), SUM149 (149) and BT549 (549) cells in the exponential growth phase were harvested, and nuclear and cytoplasmic protein samples were isolated. The expression of Aurora A (AURKA), β-actin and histone H3 was determined via immunoblotting (IB).

(b)-(f) AURKA (green) distribution was analyzed via immunofluorescence (IF)

staining in MCF-10A (10A), MDA-MB-231 (231), Sk-br-3 (sk), SUM149 (149) and BT549 (549) cells in the exponential growth phase. γ-Tubulin (red) was used to indicate centrosome location. The nuclei were stained with DAPI (blue). Scale bar, 50 μm.

(g) The treatment was similar to Fig. 1d. Cells were subjected to ALDEFLUOR staining and analysis by flow cytometry.

(h) The treatment was similar to Fig. 1e. Cells were subjected to ALDEFLUOR staining and analysis flow cytometry.

(i) MDA-MB-231 cells (2×10^5), which expressed shRNA against AURKA under the control of a Tet-inducible system, were treated with doxycycline for 24 h. Then, the cells were transfected with lentivirus expressing AURKA (WT, KA or KD) or the AURKA-ER (AER) fusion protein in the presence of doxycycline for 72 h. The cells were harvested for IB analysis.

(j) MDA-MB-231 cells (3×10^6) overexpressed AER via lentivirus-mediated gene transfer were treated with or without 200nM OHT treatment. Two days after transfection, the cells were selected using 1μg/ml puromycin for 2 days. The cytoplasmic and nuclear fractions were prepared and subjected to IB analysis.

(k) The cells subjected to treatment similar to that in (j) were fixed, and immunofluorescence (IF) staining was performed for HA (green). The nuclei were stained with DAPI (blue). Scale bar, 50 μm.

(l) MDA-MB-231 cells (2×10^5) were transduced with shRNA vector (sh Control or shAURKA) and over-expression vector (ER or AER) via lentivirus-mediated gene

transfer. Two days after transfection, the cells were double-selected with 1 μg/ml puromycin and 2 μg/ml blasticidin for 2 days. The adherent cells were collected for IB analysis.

(m) SUM149 cells (2×10^5) were co-transfected with shRNA vector (sh Control or shAURKA) and the overexpression vector (ER or AER) via lentivirus-mediated gene transfer. Two days after transfection, the cells were double-selected using 1 μg/ml puromycin and 2 μg/ml blasticidin for 2 days. The resultant cells were used to perform first round mammosphere culture for 9 days (upper panel). At the end of the first round mammosphere culture, the cells were photographed. Then, the single suspension was prepared from sphere from the first round culture and subjected to second round mammosphere culture for 9 days (lower panel). At the end of the second round mammosphere culture, the cells were photographed. Scale bar, 100 μm.

(n) Data from three independent experiments derived from (m) were used for quantitative analysis of the mammosphere size (diameter, Φ) and the mammosphere number. The left panel showed the distribution pattern of the mammosphere size from SUM149 cells (Kruskal-Wallis test followed by Dunn's Multiple Comparison test, ***$p < 0.001$). The right panel showed the number of mammosphere (Φ>60 μm), the bars represented the means ± SEM (ANOVA followed by the LSD test, ***$p < 0.001$).

(o) The cells in Fig. 1g used to perform two round mammosphere culture were subjected to a proliferation assay in conventional adherent culture by cell counting.

(p) The cells used in (m) to perform two round mammosphere culture were subjected to a proliferation in conventional adherent culture by cell counting.

Supplementary Fig. 2

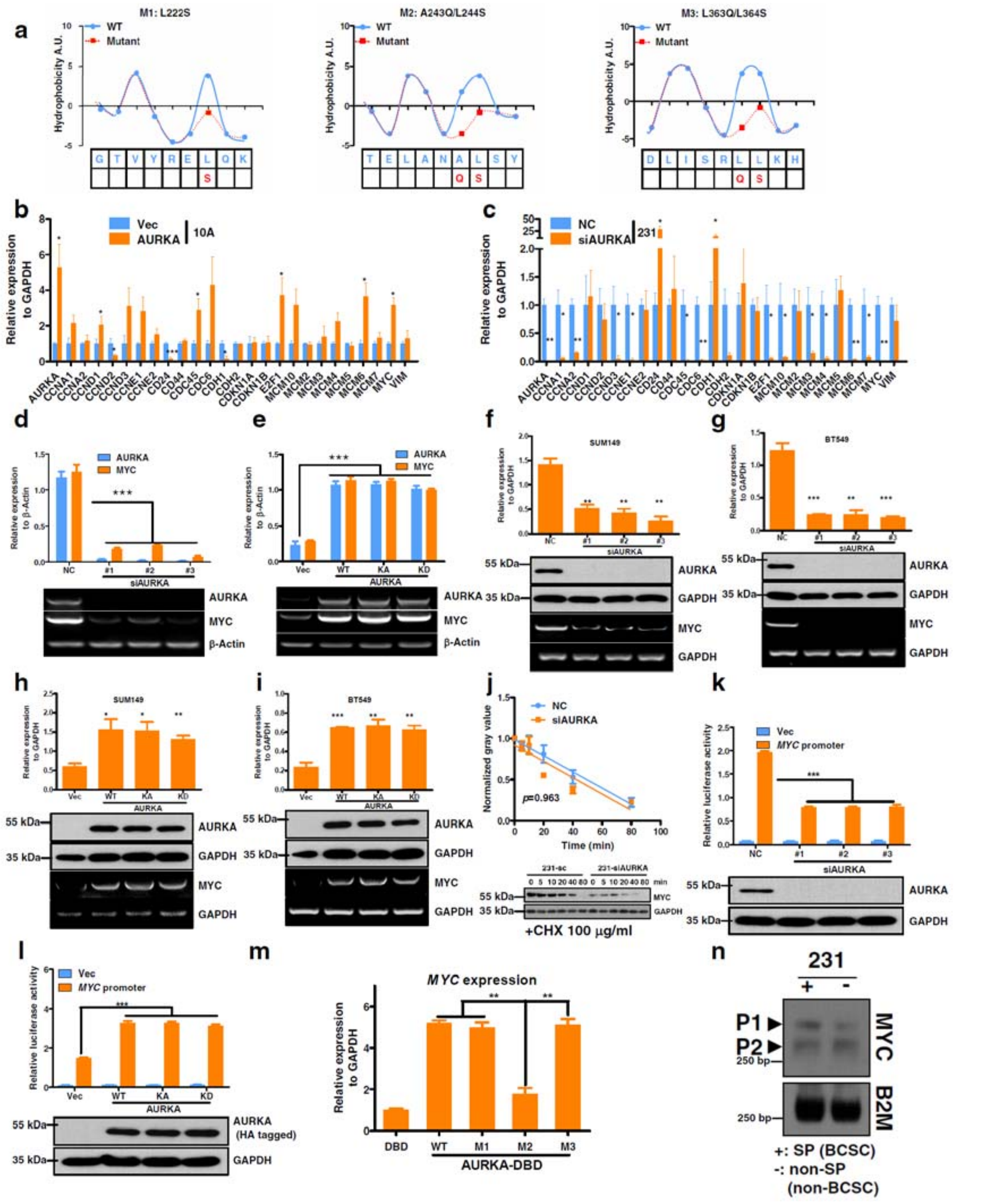

Supplementary Fig. 2 Related to Fig. 2

(a) Schematic diagram showing the hydrophobicity of three potential WT TADs (blue line) or mutant TADs (red line) in AURKA.

(b) and (c) MCF-10A (3×10^6) overexpressed 20 μg of AURKA and 3×10^6

MDA-MB-231 treated with 400 pmol and the corresponding control cell lines were subjected to RNA extraction. The expression of indicated genes were determined by realtime PCR. The bar represents means ± SEM of three independent experiments (t test, *: p≤ 0.05, **: p≤ 0.01).

(d) AURKA was down-regulated in MDA-MB-231 cells (2×10^5) by treatment with three different siRNA (60 pmol) for 48 h. The expression of AURKA, c-Myc and β-Actin mRNA was determined via semi-quantitative PCR. The bars represented the means ± SEM of three independent experiments (ANOVA followed by the LSD test, ***: p≤ 0.001).

(e) One μg of empty vector (Vec) or a vector expressing AURKA (WT, KA or KD) was transfected into 5×10^5 MDA-MB-231 cells for 48 h. The expression of AURKA, c-Myc and β-Actin mRNA was determined via semi-quantitative PCR. The bars represented the means ± SEM of three independent experiments (ANOVA followed by the LSD test, ***: p≤ 0.001).

(f) and (g) SUM149 and BT549 cells (2×10^5) were treated with 60 pmol AURKA or scramble siRNA for 48 h. The expression of c-Myc and β-Actin mRNA was determined via semi-quantitative PCR, and AURKA and GAPDH were determined via IB. The bar represented the means ± SEM of three independent experiments (ANOVA followed by the LSD test, *: p≤ 0.05, **: p≤ 0.01, ***: p≤ 0.001).

(h) and (i) One μg of empty empty vector (Vec) or vector expressing AURKA (WT, KA or KD) was transfected into 5×10^5 SUM149 and BT549 cells for 48 h. The expression of c-Myc and β-Actin mRNA was determined by semiquantitative PCR,

AURKA and GAPDH expression determined by IB. The bar represented the means ± SEM of three independent experiments (ANOVA followed by the LSD test, **: p≤ 0.01, ***: p≤ 0.001).

(j) MDA-MB-231 cells (5×10^5) were treated with 60 pmol siRNA against AURKA or scramble for 48 h. Then, the cells were then treated with 100 μg/ml cycloheximide (CHX) for the indicated period and were harvested for IB analysis. The expression of c-Myc and GAPDH was determined via IB (left panel). The IB results of c-Myc were quantified using Image J and were normalized to those of GAPDH. The normalized c-Myc expression level was used to construct a scatter plot (right panel). Linear regression was performed for statistical analysis.

(k) Scramble or AURKA siRNA (60 pmol) were transfected into 5×10^5 MDA-MB-231 cells for 24 h. Then 0.5 μg of *MYC* promoter or an empty basic reporter and 0.05 μg of pRL-TK plasmid were co-transfected into MDA-MB-231 cells for another 24 h. Dual luciferase reporter assays were performed to evaluate *MYC* promoter activation. The bars represented the means ± SEM of three independent experiments (ANOVA followed by the LSD test, ***: p≤ 0.001).

(l) One μg of empty vector (Vec) or a vector expressing AURKA (WT, KA or KD), 0.5 μg of *MYC* promoter reporter or a basic reporter and 0.05 μg of pRL-TK plasmid were co-transfected into 5×10^5 MDA-MB-231 cells for 24 h. Dual luciferase reporter assays were performed to evaluate *MYC* promoter activity. The bars represented the means ± SEM of three independent experiments (ANOVA followed by the LSD test, **: p≤ 0.01, ***: p≤ 0.001).

(m) One μg of DBD/AURKA-DBD (WT, M1, M2 or M3) was transfected into 5×10^5 293T cells. After 24 h, the cells were harvested for real time PCR analysis.

(n) BCSC and non-BCSC were isolated according to their side population level by flow cytometry. RNA of these cells was isolated and subjected to S1 nuclease protection assay to monitor the *MYC* P1 and P2 transcripts. The level of B2M mRNA in parallel identical samples were also determined.

Supplementary Fig. 3

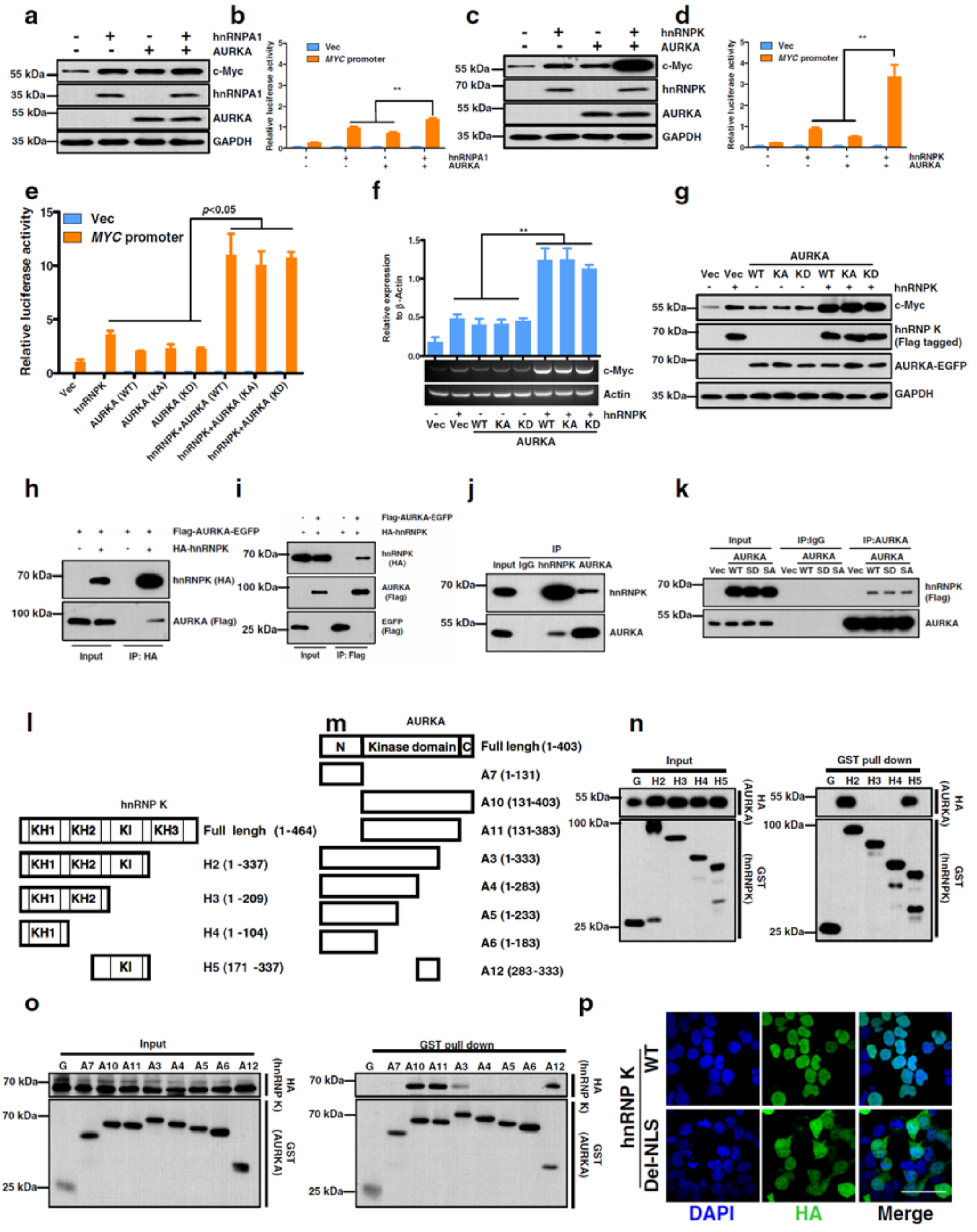

Supplementary Fig. 3 Related to Fig. 3

(a) and (b) MDA-MB-231 cells (5×10^5) were co-transfected with 1 μg of AURKA and/or hnRNP A1 and 0.5 μg of *MYC* promoter reporter (MYC) or basic reporter (Vec) along with 0.05 μg of pRL-TK plasmid for 24 h. The expression of c-Myc, hnRNP A1,

AURKA and GAPDH were confirmed via IB (a). The *MYC* promoter activity was determined via a dual luciferase reporter assay (b). The bars represented the means ± SEM of three independent experiments (ANOVA followed by the LSD test, **: $p \leq 0.01$).

(c) and (d) MDA-MB-231 cells (5×10^5) were co-transfected with 1 μg of AURKA and/or hnRNP K and 0.5 μg of *MYC* promoter reporter (MYC) or basic reporter (Vec) along with 0.05 μg of pRL-TK plasmid for 24 h. The expression of c-Myc, hnRNP K, AURKA and GAPDH were determined via IB (c). The *MYC* promoter activity was determined via a dual luciferase reporter assay (d). The bars represented the means ± SEM of three independent experiments (ANOVA followed by the LSD test, **: $p \leq 0.01$).

(e), (f) and (g) MDA-MB-231 cells (5×10^5) were co-transfected with 1 μg of expression vectors for the AURKA mutants and hnRNP K and 0.5 μg of *MYC* promoter reporter (MYC) or basic reporter (Vec) along with 0.05 μg of pRL-TK plasmid for 24 h. *MYC* promoter activity was determined via a dual luciferase reporter assay (e). The expression of c-Myc, hnRNP K, AURKA and GAPDH were analyzed via RT-PCR (f) and IB (g). The bars represented the means ± SEM of three independent experiments (ANOVA followed by the LSD test, **: $p \leq 0.01$, ***: $p \leq 0.001$).

(h) 293T cells (3×10^6) were co-transfected with 20 μg of Flag-AURKA-EGFP and hnRNP K-HA for 24 h. The cells were lyzsed and IP was performed using a anti-HA antibody. The expression of Flag-tagged AURKA and HA-tagged hnRNP K was

determined via IB.

(i) 293T cells (3×10^6) were co-transfected with 20 μg of HA-hnRNP K and Flag-AURKA-EGFP for 24 h. The cells were lysed and IP was performed using an anti-Flag antibody. The expression of Flag-tagged AURKA and HA-tagged hnRNP K was determined via IB.

(j) MDA-MB-231 cells (6×10^6) in the exponential growth phase were lysed, and IP was performed using antibodies against hnRNP K, AURKA or control IgG. The expression of AURKA and hnRNP K was determined via IB.

(k) Twenty μg of Flag-tagged hnRNP K (WT, SD or SA) expression plasmids were transfected into 3×10^6 293T cells. After 24 h, the cells were lysed and IP was performed using an anti-AURKA antibody. The expression of Flag-tagged hnRNP K and AURKA were determined by IB.

(l) Schematic diagram of the GST-fused hnRNP K truncated mutants.

(m) Schematic diagram of the GST-fused AURKA truncated mutants.

(n) Twenty μg of HA-tagged AURKA was overexpressed in 3×10^6 293T cells for 24 h. The cells were lysed and divided equally into 5 aliquots. 5 μg of GST-fused hnRNP K was incubated with lysates, followed by pull down of GST-fused hnRNP K to examine the expression of HA-tagged AURKA in GST-fused hnRNP K protein complexes via IB.

(o) Twenty μg of HA-tagged hnRNP K was overexpressed in 3×10^6 293T cells for 24 h. The cells were lysed and divided equally into 9 aliquots. 5 μg of GST-fused AURKA was incubated with lysates, followed by pull down of GST-fused AURKA to

examine the expression of HA-tagged hnRNP K in GST-fused AURKA protein complexes via IB.

(p) 293T cells (5×10^5) were transfected with 1 μg of wild type or NLS deleted hnRNP K for 24 h. Cells were fixed and subjected to IF staining.

Supplementary Fig. 4

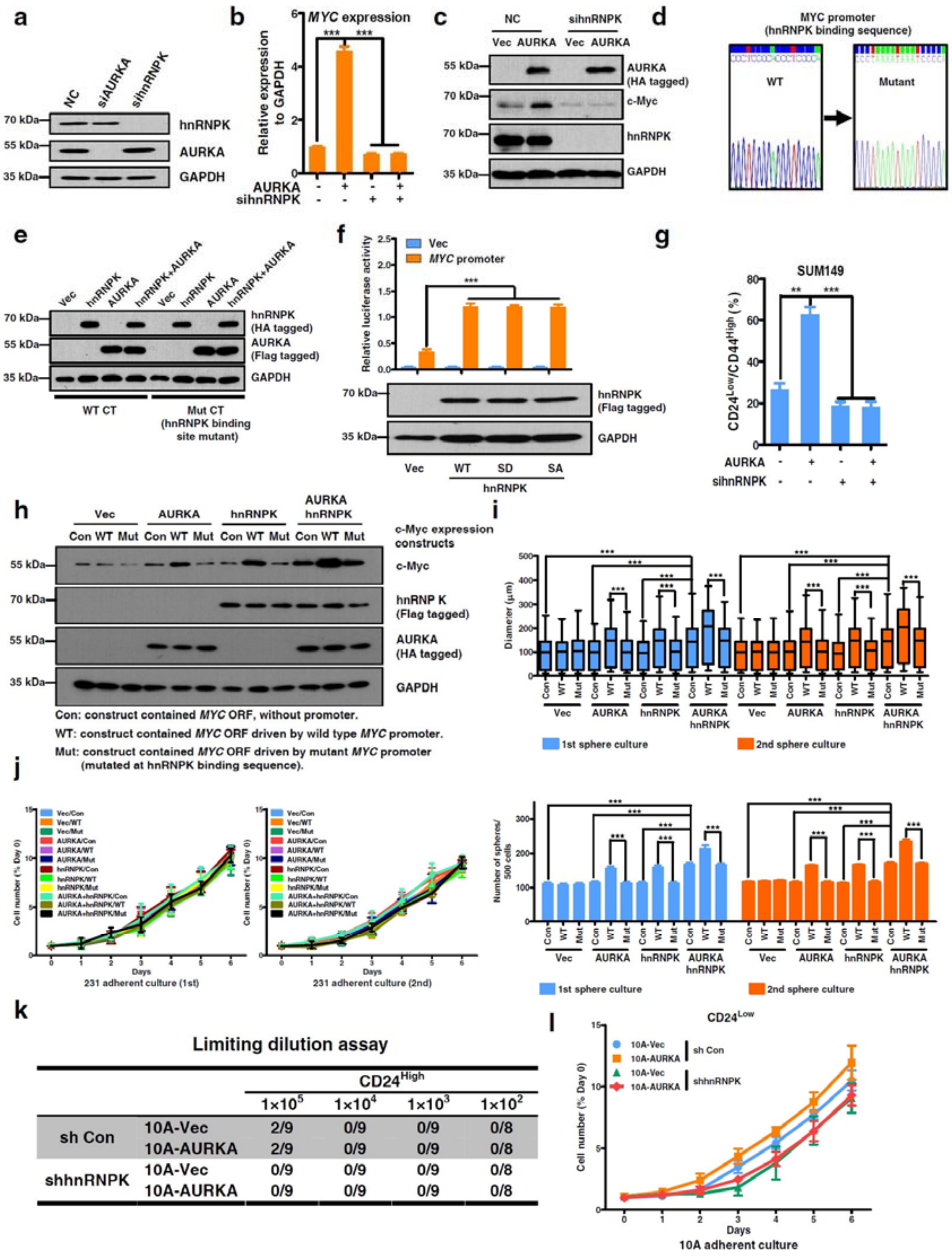

Supplementary Fig. 4 Related to Fig. 4

(a) MDA-MB-231 cells (3×10^6) were transfected with 400 pmol siRNA against negative control (NC), AURKA and hnRNP K for 48 h. The cells were subjected to IB analysis.

(b) and (c) Cells were treated as Fig. 4c and Then subjected to real time PCR (b) and IB (c) analysis.

(d) The sequencing results of the CT-element mutation of the *MYC* promoter are shown.

(e) The treatment was identical to that in Fig. 4d. The cells were collected for performing IB analysis.

(f) One μg of Flag-tagged hnRNP K (WT, SD or SA) expression plasmids, 0.5 μg of *MYC* promoter reporter or a basic reporter and 0.05 μg of pRL-TK plasmid were transfected into 5×10^5 293T cells. After 24 h, dual luciferase reporter assays were performed to evaluate *MYC* promoter activation. The bars represented the means ± SEM of three independent experiments (ANOVA followed by the LSD test, ***: $p\leq 0.001$).

(g) SUM149 cells (5×10^5) overexpressing HA-tagged AURKA were transfected with 60 pmol hnRNP K or NC siRNA. After 48 h, the $CD24^{low}/CD44^{high}$ population was analyzed via flow cytometry. The bars represented the means ± SEM of three independent experiments (ANOVA followed by the LSD test, **: $p\leq 0.01$, ***: $p\leq 0.001$).

(h) Expression of exogenous c-Myc was driven by wild-type ("WT") or mutant (mutated hnRNP K binding site, "Mut") *MYC* promoter in MDA-MB-231. Control ("Con") represented wild-type *MYC* promoter without the downstream *MYC* open reading frame. AURKA or/and hnRNP K were expressed via lentivirus-mediated gene transfer. The cells were also subjected to IB analysis.

(i) The cells established in (h) were subjected to mammosphere culture (6 days) and the secondary passaging (additional 6 days). The left panel showed the distribution pattern of mammosphere size from MDA-MB-231 (Kruskal-Wallis test followed by Dunn's Multiple Comparison test, ***$p < 0.001$). The right panel showed the number of mammosphere (Φ>60 μm).

(j) The cells used in (i) to perform two round mammosphere culture were subjected to a proliferation assay by cell counting.

(k) The treatment was the same as Fig. 4l. The population of $CD24^{High}$ were used to perform limiting dilution assays in NOD/SCID mice.

(l) The $CD24^{Low}$ cells sorted in Fig. 4l were subjected to evaluate proliferation by cell counting.

Supplementary Fig. 5

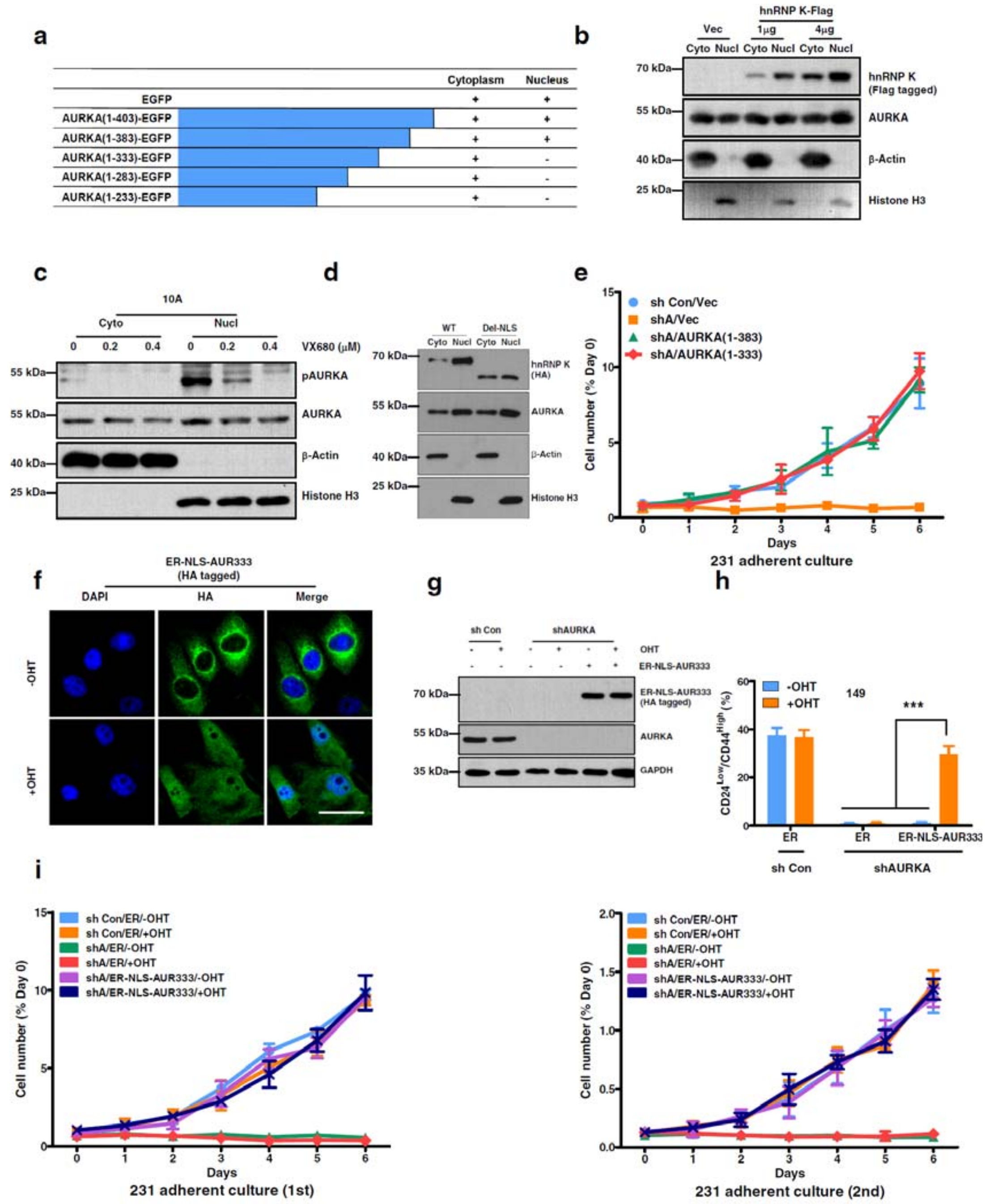

Supplementary Fig. 5 Related to Fig. 5

(a) The results of Fig. 5a were summarized.

(b) Different amounts of hnRNP K (0 μg, 1 μg or 4 μg) were expressed in 5×10^5 MDA-MB-231 cells for 24 h. The cytoplasmic and nuclear proteins were extracted and subjected to IB analysis.

(c) MCF-10A cells (6×10^6) were treated with the indicated concentrations of VX-680 for 24 h. The cytoplasmic and nuclear proteins were extracted and subjected to IB analysis.

(d) Twenty μg of wild type or NLS deletion mutant of hnRNP K were expressed in 3×10^6 MDA-MB-231 cells for 24 h. The cytoplasmic and nuclear proteins were extracted and subjected to IB analysis.

(e) The cells in Fig. 5e were subjected to evaluate proliferation in adherent culture by cell counting.

(f) MDA-MB-231 cells were infected with lentivirus expressing a fusion protein (ER-NLS-AUR333) and were treated with or without 200nM OHT for 48 h. The cells were fixed and IF staining was performed against the HA-tag. Scale bar, 50 μm.

(g) MDA-MB-231 cells were co-infected with lentivirus expressing AURKA shRNA (shAURKA or sh Control) and ER-NLS-AUR333. Then the cells were replaced with fresh medium and cultured for 48 h in the presence or absence of 200nM OHT treatment. The cells were selected in medium containing or lacking OHT in the presence of 1 μg/ml puromycin and 5 μg/ml blasticidin for 48 h. The expression levels of AURKA (endogenous), ER-NLS-AUR333 and GAPDH were determined via IB.

(h) SUM149 cells were co-infected with lentivirus expressing AURKA shRNA (shAURKA or sh Control) and ER-NLS-AUR333. Then, the cells were cultured in fresh medium for 48 h in the presence or absence of 200nM OHT. The cells were selected for in medium containing or lacking OHT and in the presence of 1 μg/ml

puromycin and 5 μg/ml blasticidin for 48 h, before harvested for $CD24^{low}/CD44^{high}$ population analysis by flow cytometry. The bars represented the means ± SEM of three independent experiments (ANOVA followed by the LSD test, ***: $p \leq 0.001$).

(i) The cells used in Fig.5g to perform two rounds of mammosphere cultures were subjected to evaluate proliferation in adherent culture by cell counting.

Supplementary Fig. 6

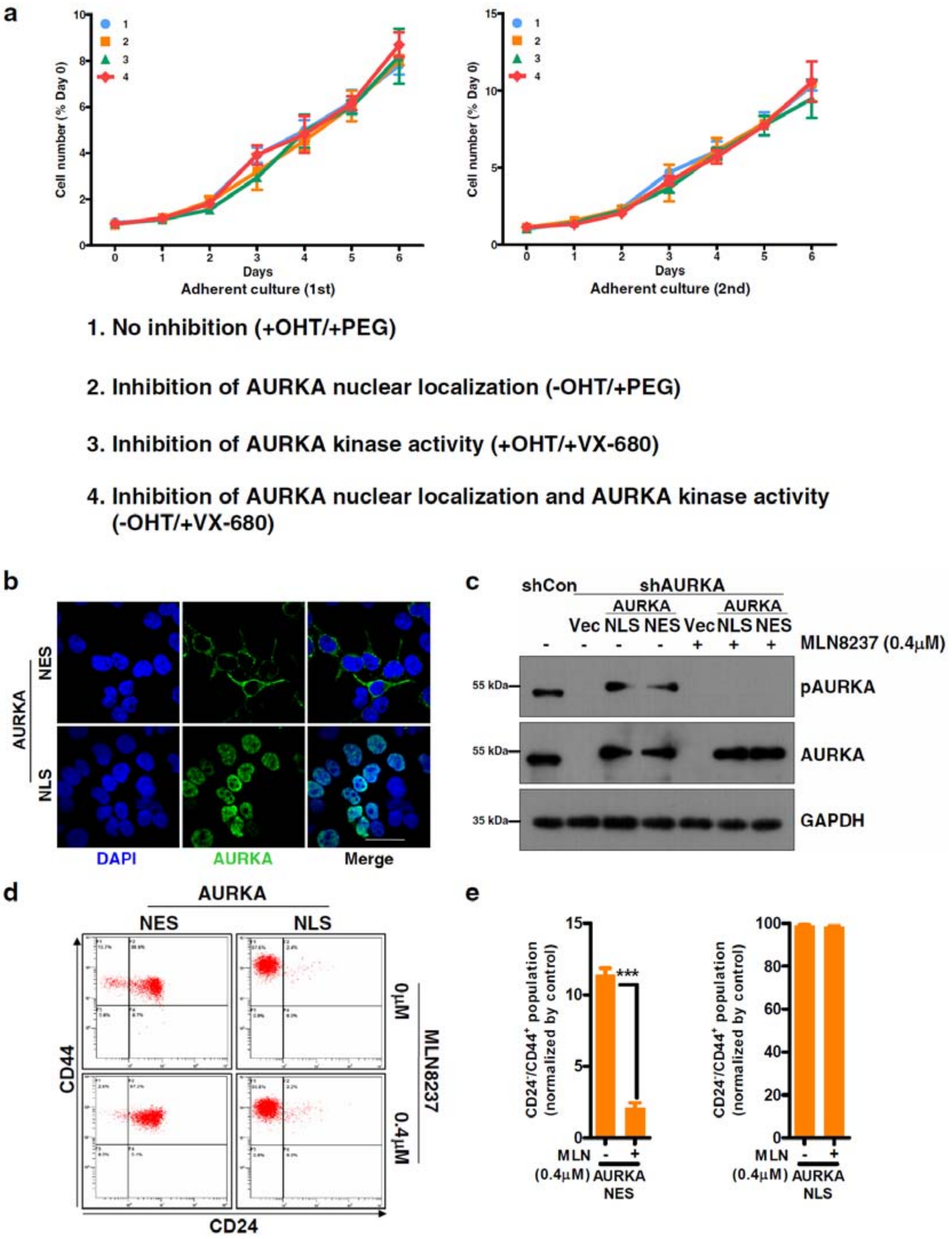

Supplementary Fig. 6 Related to Fig. 6

(a) The cells used in Fig. 6f to perform two rounds mammosphere cultures were assayed for proliferation in adherent culture by cell counting.

(b)-(e) MDA-MB-231 cells were infected with lentivirus expressed AURKA shRNA

for 24 h. Then, the lentivirus infected MDA-MB-231 cells (5×10^5) were transfected with 1 μg of the NES or NLS fused AURKA for 24 h. Then, cells were propagated, and were treated with or without 0.4 μM MLN8237 or vehicle for 48 h. The cells were collected for IF staining (b), IB (c) and CD24/CD44 staining (d) analysis. The results in (d) are summarized in (e).

Supplementary Fig. 7

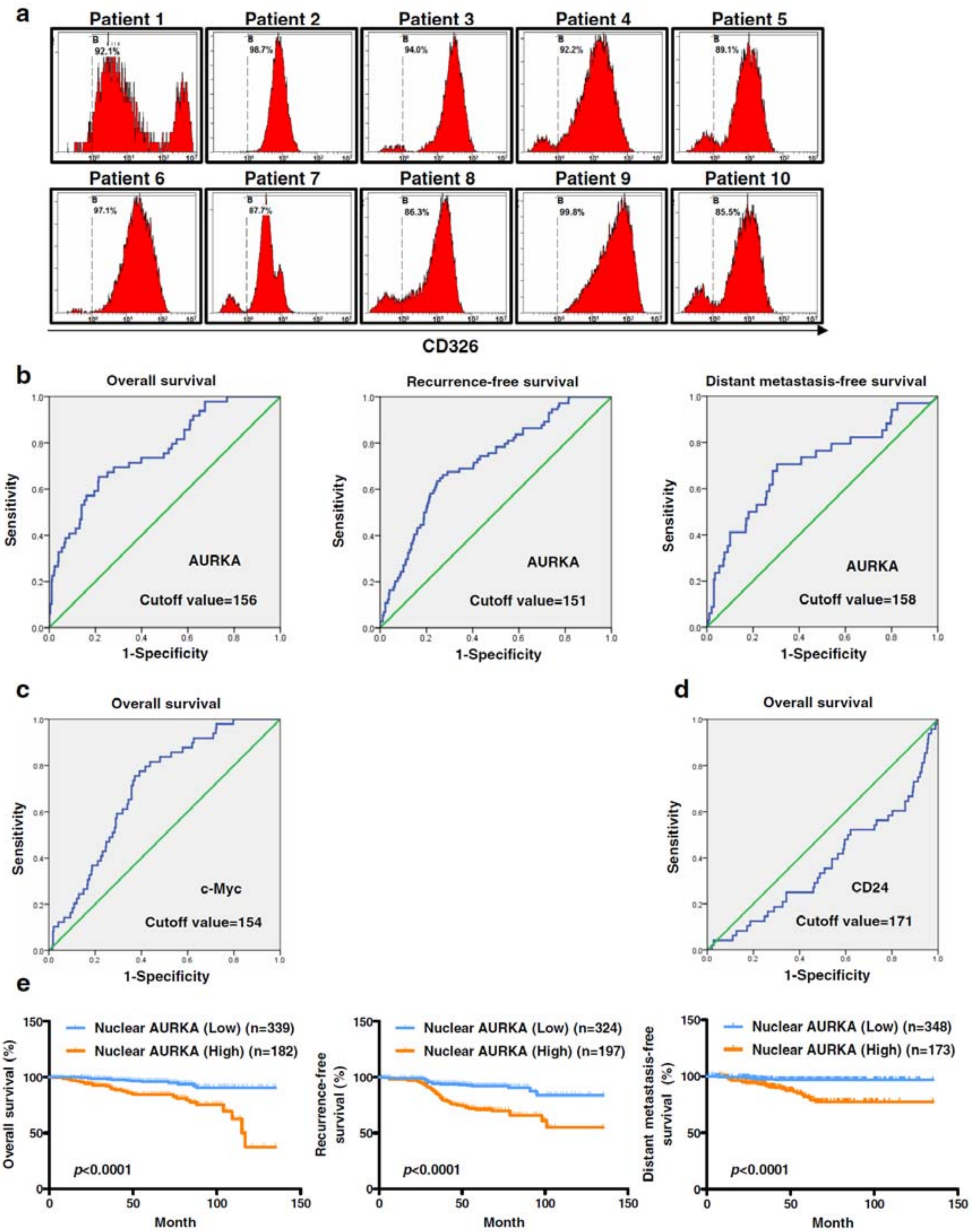

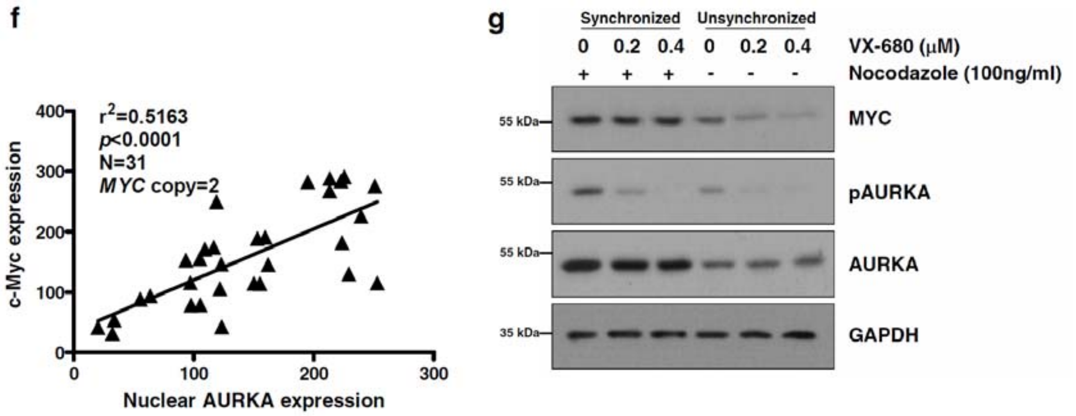

Supplementary Fig. 7 Related to Fig. 7

(a) The primary breast cancer cells shown in Fig. 7a were analyzed for the expression of CD326 via flow cytometry.

(b)-(d) IHC staining of AURKA, c-Myc and CD24, respectively, were performed on the breast cancer tissues. The cutoff values were determined based on the receiver operating characteristic (ROC) curve.

(e) IHC staining of AURKA was performed on the breast cancer tissues.. Kaplan-Meier analysis was performed and the log-rank test was used for statistical analysis.

(f) Breast cancer samples (N=59) were subjected to FISH assay to identify their *MYC* copy number. Samples were also subjected to IHC staining of AURKA and c-Myc. The samples (N=31) with normal *MYC* copy number (2 copies) and with paired AURKA and c-Myc staining were scored and subjected to linear regression analysis.

(g) MDA-MB-231 cells were synchronized at M phase by treating with 100 ng/ml nocodazole for 12 h. M phase cells were collected. The M phase cells were treated with 100 ng/ml nocodazole and indicated concentration of VX-680 for 36 h. The control cells (unsynchronized cells) were treated with indicated concentration of

VX-680 for 36 h. Cells were subjected to IB analysis.

Supplementary Fig. 8

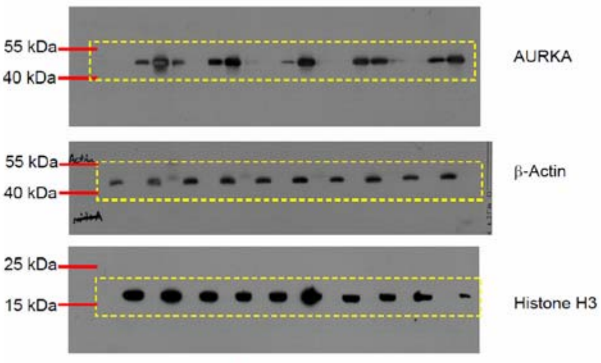

Full unedited gel for Figure 1a

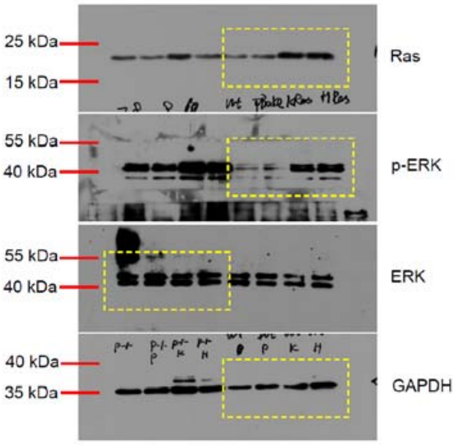

Full unedited gel for Figure 1c left panel

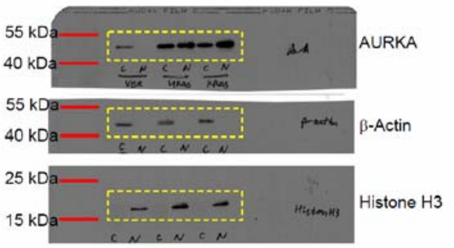

Full unedited gel for Figure 1c right panel

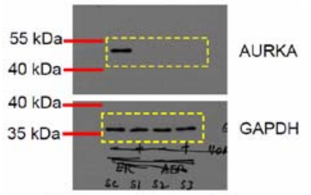

Full unedited gel for Figure 1d left panel

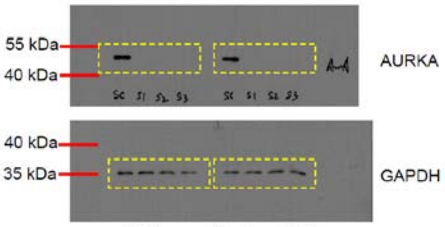

Full unedited gel for Figure 1d left panel

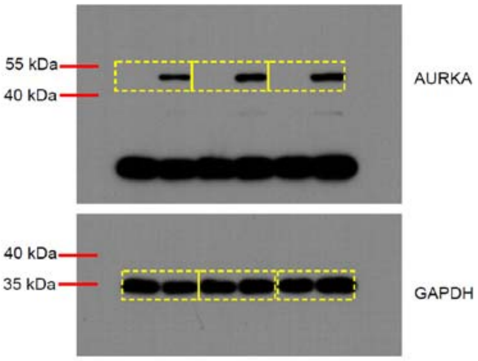

Full unedited gel for Figure 1e left panel

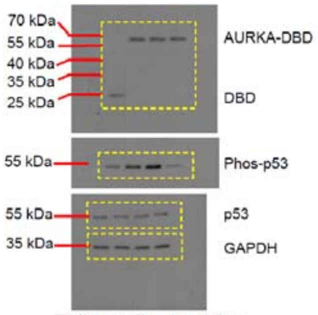

Full unedited gel for
Figure 2a left panel

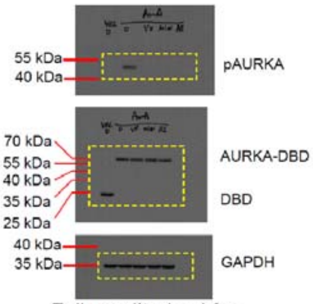

Full unedited gel for
Figure 2b left panel

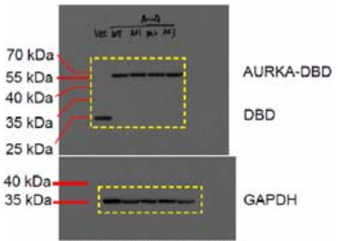

Full unedited gel for
Figure 2d left panel

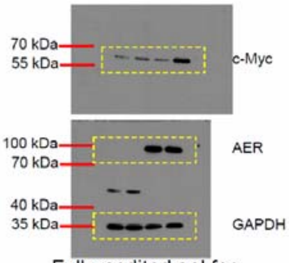

Full unedited gel for
Figure 2g lower panel

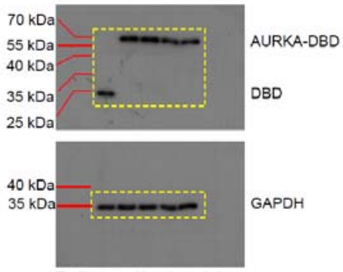

Full unedited gel for
Figure 2h left panel

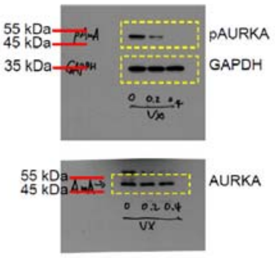

Full unedited gel for
Figure 2k lower panel

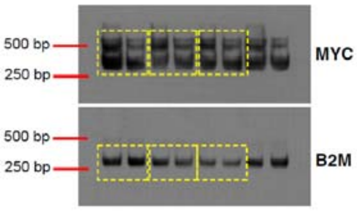

Full unedited gel for
Figure 2l lower panel

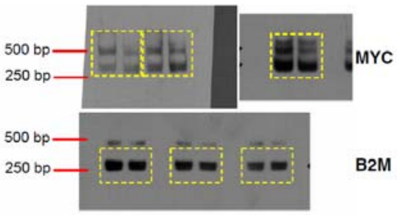

Full unedited gel for
Figure 2m lower panel

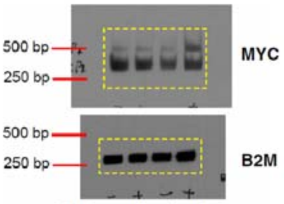

Full unedited gel for
Figure 2n lower panel

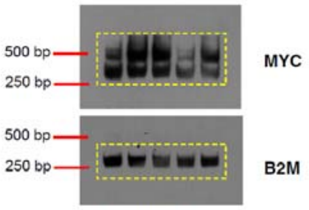

Full unedited gel for
Figure 2o lower panel

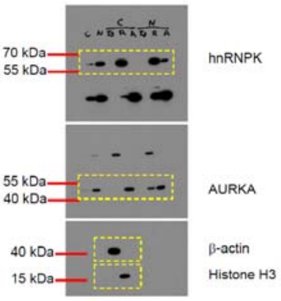

Full unedited gel for Figure 3d

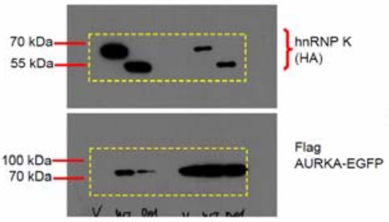

Full unedited gel for Figure 3f

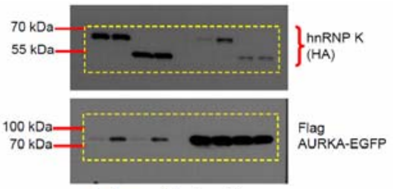

Full unedited gel for Figure 3g

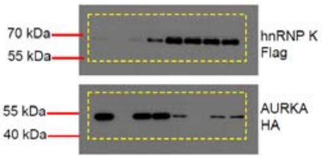

Full unedited gel for Figure 3h

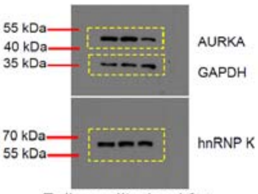

Full unedited gel for Figure 3j

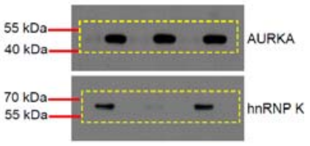

Full unedited gel for Figure 3k

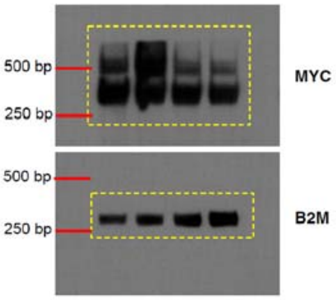

Full unedited gel for Figure 4e

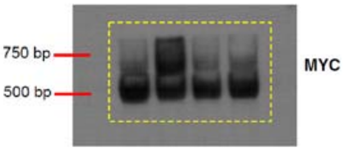

Full unedited gel for Figure 4g

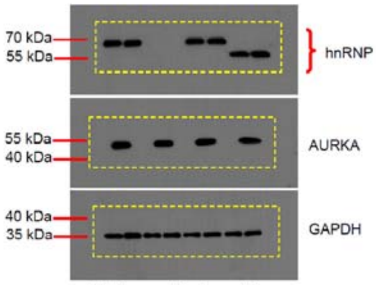

Full unedited gel for Figure 4h

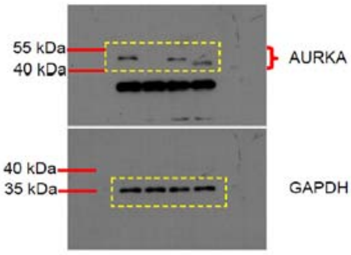

Full unedited gel for Figure 5c

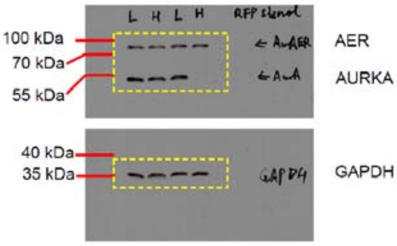

Full unedited gel for Figure 6a

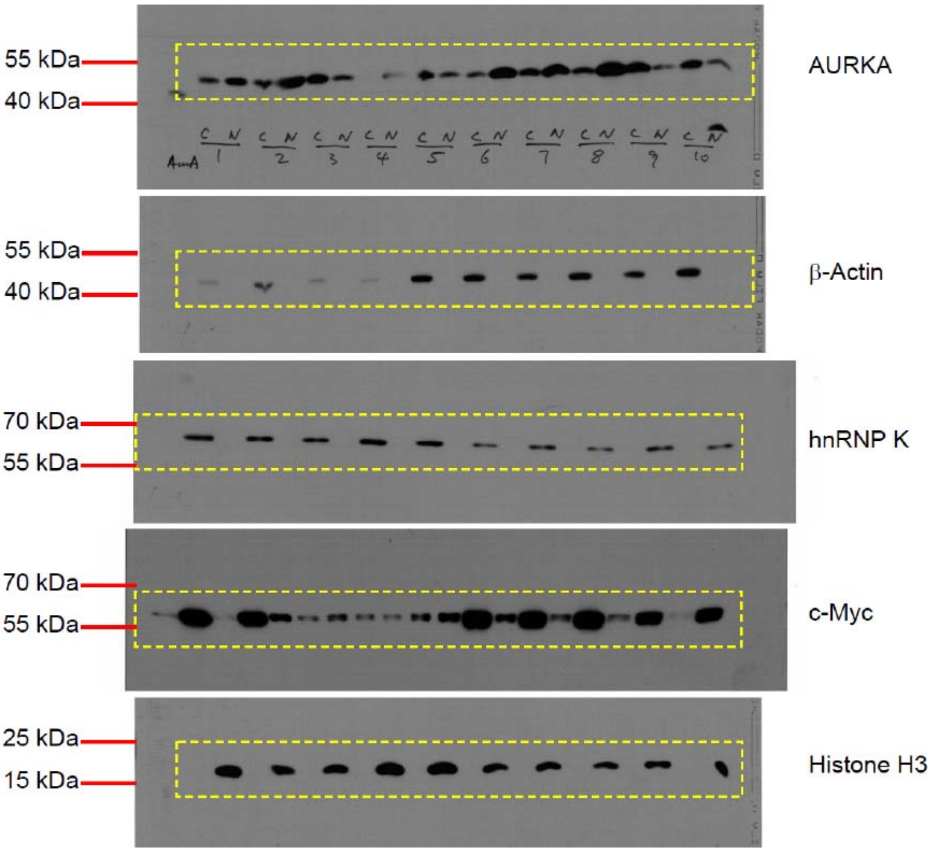

Full unedited gel for Figure 7A

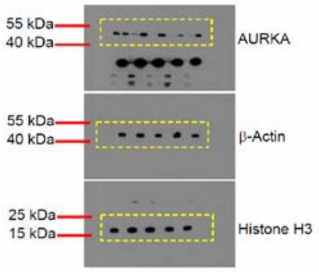

Full unedited gel for
Supplemental Figure 1a

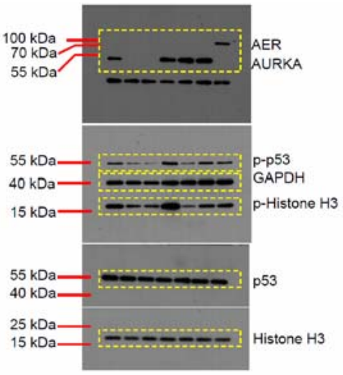

Full unedited gel for
Supplemental Figure 1i

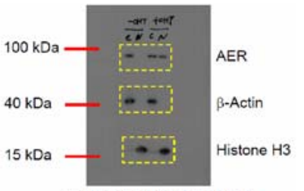

Full unedited gel for
Supplemental Figure 1j

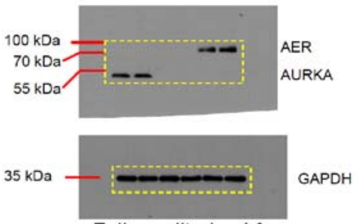

Full unedited gel for
Supplemental Figure 1l

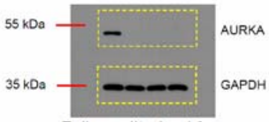

Full unedited gel for
Supplemental Figure 2f

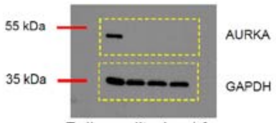

Full unedited gel for
Supplemental Figure 2g

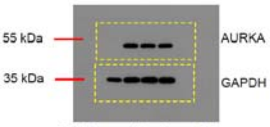

Full unedited gel for
Supplemental Figure 2h

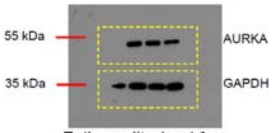

Full unedited gel for
Supplemental Figure 2i

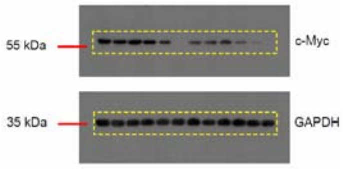

Full unedited gel for
Supplemental Figure 2j

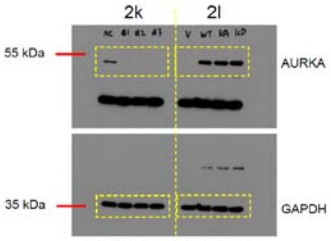

Full unedited gel for
Supplemental Figure 2k and 2l

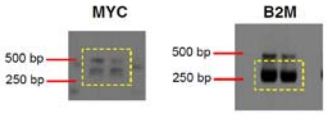

Full unedited gel for Supplemental Figure 2n

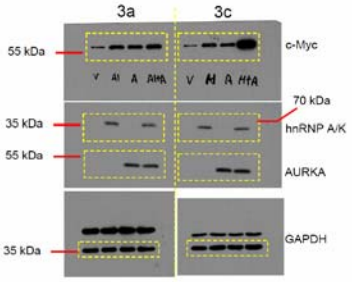

Full unedited gel for
Supplemental Figure 3a and 3c

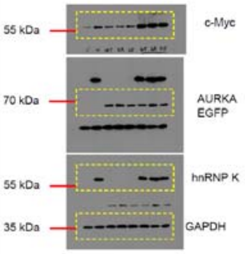

Full unedited gel for
Supplemental Figure 3g

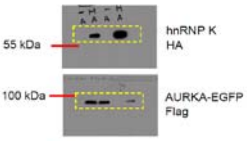

Full unedited gel for
Supplemental Figure 3h

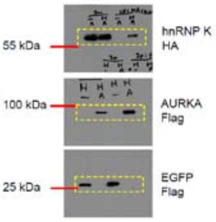

Full unedited gel for
Supplemental Figure 3i

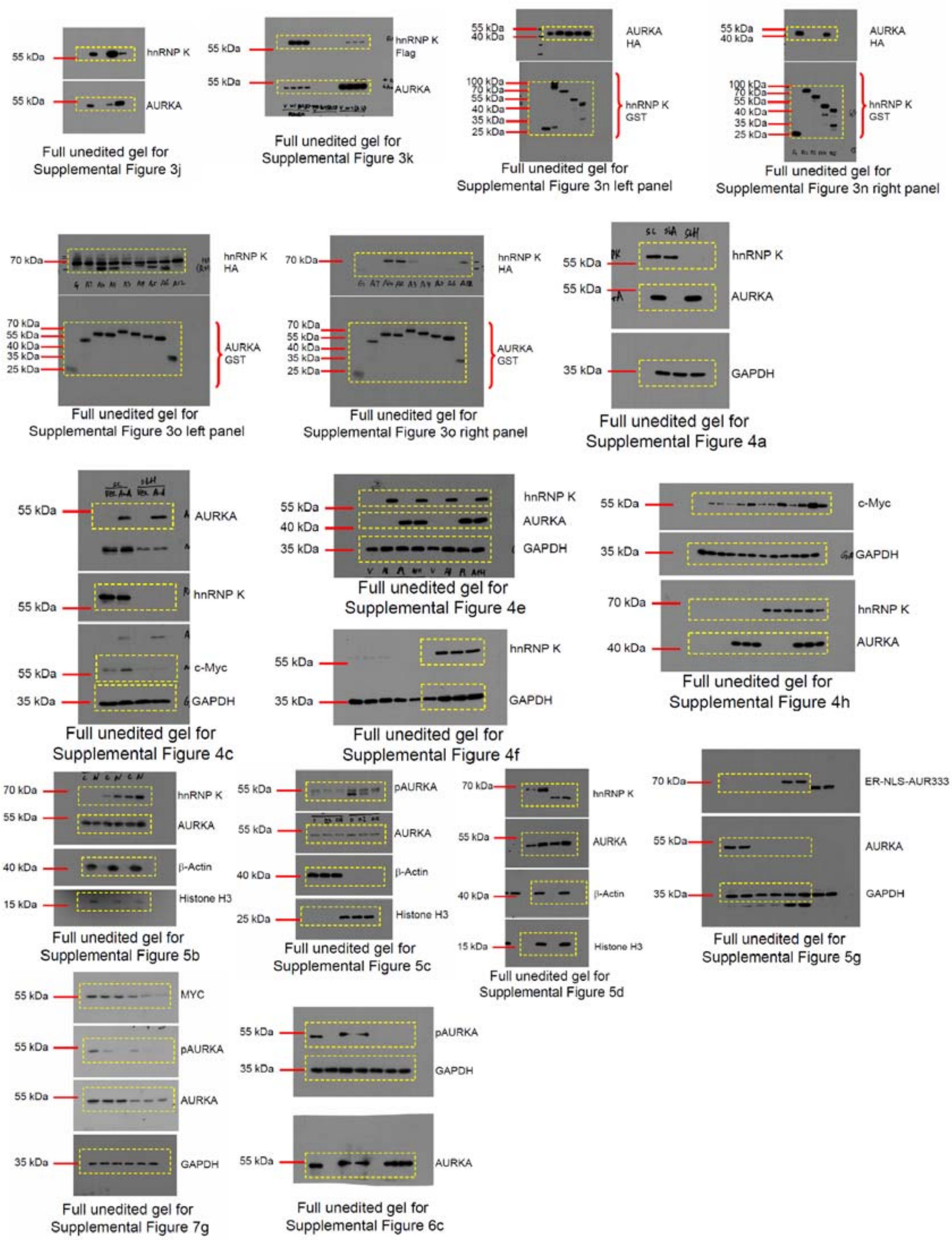

Supplementary Fig. 7 Uncropped images of blots. Uncropped scans of blots shown in the Figs 1a, 1c, 1d, 1e, 2a, 2b, 2d, 2g, 2h, 2k, 2l, 2m, 2n, 2o, 3d, 3f, 3g, 3h, 3j, 3k, 4e, 4g, 4h, 5c, 6a, 7a, and supplementary Figs.1a, 1i, 1j, 1l, 2f, 2g, 2h, 2i, 2j, 2k, 2l, 2n, 3a, 3c, 3g, 3h, 3i, 3j, 3k, 3n, 3o, 4a, 4c, 4e, 4f, 4h, 5b, 5c, 5d, 5g, 6c, 7g.

Supplementary Table 1

Differentially expressed genes regulated by AURKA are identified by RNA sequencing.

Symbol	log2 Ratio (10A-A2/10A-P2)	Symbol	log2 Ratio (10A-A2/10A-P2)	Symbol	log2 Ratio (10A-A2/10A-P2)
SIRT2	10.509775	KLF10	1.15444818	SLC1A4	-1.919557007
SCNN1G	10.39874369	ZNF598	1.153753546	FAM84A	-1.913179241
FAM65B	9.884170519	GADD45GIP1	1.152879931	PDZK1IP1	-1.903284144
SLCO4A1	9.214319121	SUV39H1	1.152547591	CLMN	-1.878543708
IKZF2	8.924812504	GTPBP4	1.152469772	GAS1	-1.870432736
LENG9	8.45532722	FDX1	1.152105919	PVRL4	-1.866431799
SAMSN1	5.442943496	THOP1	1.148478372	CA2	-1.866431799
DUSP1	4.423694361	NOC4L	1.147539503	CCDC80	-1.866078972
NUDT11	3.807354922	RAD18	1.147050535	SPARC	-1.857404329
GDA	3.693228803	PITPNC1	1.145796731	DOPEY1	-1.85705944
MAOA	3.475433213	DEPDC1	1.136863606	KLRC2	-1.842398277
KLF9	3.169925001	BTF3L4	1.135458848	PGBD5	-1.840059087
PRSS3	3.058893689	RPUSD2	1.135340765	TP53INP1	-1.831952286
FOS	3.030742649	YDJC	1.135079534	GPR110	-1.824599868
TSPAN18	2.925999419	UBE2E2	1.1318415	IFFO2	-1.82196018
DSEL	2.807354922	DNAJC11	1.12988766	MTUS1	-1.806336789
ASNS	2.800919097	MRPL4	1.128153797	KRT80	-1.806313641
FKBP5	2.607932354	RABEPK	1.127348565	SLC16A2	-1.800710721
SLC19A1	2.584962501	DUSP4	1.124057417	ANKRD37	-1.790225486
OSBPL6	2.560714954	DDX56	1.123254568	LY6D	-1.783409226
E2F1	2.544320516	PRPS2	1.121007204	DSG3	-1.780000039
SPRY4	2.475733431	ZNF593	1.117482766	CDH1	-1.770295763
CCL20	2.432464571	ABLIM3	1.114205362	TUFT1	-1.760966908
POLR3G	2.280107919	ATAD3A	1.113956189	HEPHL1	-1.754725279
HSD17B2	2.243389219	MCM5	1.113585596	C1orf133	-1.745489666
CDCA5	2.230297619	C3orf75	1.112814872	ALDH3A1	-1.731874311
TFCP2L1	2.212449618	KIF2C	1.111076327	BNIP3L	-1.729900402
FOSL1	2.20511443	BOP1	1.110602199	SELENBP1	-1.728504015
MYO19	2.169925001	IFRD2	1.110107067	FGD6	-1.719644684
MRPS30	2.137503524	MT1F	1.105673107	F2R	-1.718937602
MLPH	2.134598267	CDK4	1.104894068	C1orf116	-1.716480383
CSNK2A1	2.111852974	EHD4	1.10426243	NOTCH1	-1.712149274
C13orf15	2.10433666	CCDC90A	1.102781686	TMEM184A	-1.706728364
LYAR	2.101879614	TRIP13	1.0990326	SLC29A3	-1.699068986
LPIN2	2.099535674	RPIA	1.096316777	CELSR2	-1.695101763
GCNT1	2.078002512	NUF2	1.095588045	TNFRSF21	-1.694673872
MCM10	2.064130337	RNF26	1.09511867	PLSCR4	-1.692925033
COPS8	2.062520738	AOX1	1.094683119	BDNF	-1.67723677
EIF4EBP1	2.057191107	UBE2T	1.091832283	SSBP3	-1.673311593
HES7	2.044394119	EFNB2	1.091764607	TP63	-1.671983443
PASK	2.044394119	SMAGP	1.0912826	SMPD1	-1.671377253
AKAP12	2.019899557	PPAT	1.089870231	USP16	-1.671377253
ITGA10	2.017073513	PLXNA2	1.086591678	AHNAK2	-1.671294889
SLC7A2	2	SIAH2	1.082773052	C7orf60	-1.670134086
VSTM2L	1.9727516	SLC6A15	1.081708677	FHL2	-1.662846179
LOC728192	1.963474124	KPNA2	1.081252874	SLC16A4	-1.661839666
MARS	1.956298691	ACOT7	1.081079606	IPW	-1.66175925
VARS2	1.938599455	H2AFX	1.080956922	PDK1	-1.650861795
ANXA6	1.925999419	POLR1B	1.07948753	FAT2	-1.642225501
CDA	1.906890596	SNAI2	1.076049422	VWA5A	-1.617666666
MRPL52	1.835047752	GNG4	1.073520517	USP8	-1.616551191
MIER2	1.823122238	PDCD2L	1.073239855	PCMTD1	-1.607933023
AURKB	1.813948227	GFM1	1.072362706	NR1D2	-1.599488349
DUSP23	1.803921047	ARL6IP4	1.070192056	LIMCH1	-1.598810707

CAD	1.792575704	GPS1	1.069177488	GLIPR1	-1.593005421
MED8	1.791998161	TMX2	1.067022477	RELB	-1.584024772
DNER	1.79130774	EIF3B	1.066848619	C5orf13	-1.584024772
CENPK	1.789647202	ANGPTL4	1.066327358	PDE7A	-1.583774608
SLC25A10	1.788495895	NOP2	1.06284899	NTF4	-1.583689722
SLC25A15	1.788495895	POLR2E	1.062494761	MMP2	-1.569449501
MFSD2A	1.786596362	HCFC1	1.0620059	PTPRK	-1.562679196
PIK3R1	1.785677009	OAF	1.061954469	MAPK8IP3	-1.55532429
RCC1	1.772653133	NDUFAF2	1.061035995	BTG1	-1.551141897
PER1	1.747904162	NUDC	1.060304902	ADAM8	-1.548893246
PHLDA2	1.746530805	PPM1G	1.059734955	VSNL1	-1.547539873
MYBL2	1.745098733	NLE1	1.059472879	CHD2	-1.547522948
SMPD2	1.72935241	C8orf55	1.057600546	NCOA7	-1.543881273
LY6K	1.721144605	ALG3	1.055943489	FZD6	-1.536468894
CADM3	1.707819249	FAM166A	1.055921236	SLC27A1	-1.536468894
PSAT1	1.706494969	ECE2	1.055818516	SOX9	-1.529143985
FKBP4	1.678889627	SNRPD1	1.05548347	NR2F2	-1.526156118
SFXN4	1.678071905	TMEM194A	1.050711843	EPHB3	-1.522787355
HIPK2	1.670288099	C21orf70	1.048822479	ARID4B	-1.51699991
QRFPR	1.662965013	CCND3	1.048781214	SOX4	-1.51578111
ZFP36	1.652076697	IRX3	1.048739343	JUP	-1.50420415
CDC45	1.648699591	NSMCE4A	1.04655347	SULF2	-1.501125791
PSCA	1.64385619	DDIT4	1.045910352	IFI6	-1.498267173
LOC100128881	1.64385619	TMTC1	1.045525201	ALDH3B1	-1.497304838
FOXO1	1.64368494	EXOSC4	1.045465931	YPEL3	-1.466860715
KATNB1	1.640457613	TOMM40	1.044999133	POFUT2	-1.465988687
RPUSD3	1.637429921	MGLL	1.044824401	KCTD11	-1.464742017
MAFB	1.637016837	IARS	1.044322094	RAB7B	-1.458106221
NCAPH	1.634960984	HNRNPAB	1.043805864	IRF6	-1.454471422
MYC	1.6316708	TBL3	1.041571692	S100A8	-1.453172829
SRM	1.623418035	GINS2	1.0407821	P4HTM	-1.448554625
MLKL	1.619596136	EXO1	1.040036746	C16orf7	-1.44656815
PID1	1.609926685	SIGMAR1	1.038624897	EEA1	-1.436848333
PRC1	1.585791398	CSK	1.03754538	PLCD3	-1.391044644
CHORDC1	1.577984181	EREG	1.036432296	SIRPA	-1.387061927
LONP1	1.572527541	MRPL12	1.032482311	DLG1	-1.383526527
RAC3	1.559685526	DTYMK	1.029879368	TBC1D23	-1.377278023
MAK16	1.558976297	CDC6	1.02937626	JUND	-1.374886144
BYSL	1.558976297	ATRIP	1.029114262	RBMS3	-1.368371727
STK39	1.552541023	ID1	1.02831904	GPCPD1	-1.367584983
MT2A	1.549454062	RNF126	1.022285545	ALCAM	-1.354349573
AREG	1.545991999	HPCAL1	1.021032372	FAM46A	-1.341639422
NUP93	1.544216945	NOP56	1.020228404	BAG1	-1.340401127
TRMT6	1.544145845	ILF2	1.019695801	ZNF638	-1.336694806
ARHGAP22	1.540568381	ATP5G1	1.01682861	MARCKS	-1.336402365
SEPW1	1.53357038	THAP4	1.016615464	C1RL	-1.330378899
NUP155	1.5334322	EEFSEC	1.015841516	PALLD	-1.329804086
CCDC9	1.528207722	PUF60	1.014158542	PLAU	-1.325859912
CD3EAP	1.528207722	ARIH2	1.00859944	C17orf91	-1.324401737
SLC39A3	1.527247003	CCDC86	1.00859944	RAP2B	-1.322592523
EIF2B4	1.509902919	HMGB2	1.007741183	CSGALNACT1	-1.321105812
KRT6A	1.504736197	SNRPA1	1.005337263	CYFIP2	-1.320740203
CECR5	1.501429099	SLC43A3	1.005236035	KRT14	-1.31752395
RNASEH2A	1.498515377	YARS	1.001002452	DDR1	-1.312431607
CSF3	1.495889933	PGAM5	1.000786138	CYBASC3	-1.311967573
MTHFD2	1.495129383	SORD	1.000747704	UACA	-1.308956947
PHGDH	1.479710346	CHPT1	1.000727348	AKR1C3	-1.308187117
NIP7	1.467188062	BRIX1	1.000556918	CYBRD1	-1.305524015
RPL11	1.464585842	MRPS2	1.000556918	CRIM1	-1.304584625
LRFN3	1.453717967	RNASEH1	1.000453037	KIAA0319L	-1.304507465

GPT2	1.450116364	EMG1	1.000445482	P4HA1	-1.302427376
SH2D2A	1.446130965	RFT1	1.000376437	ANXA1	-1.302276281
PFKM	1.439760553	OLR1	-11.16302064	HES2	-1.297198401
OSBPL5	1.439460973	ELF3	-10.50878516	CD68	-1.291690864
TCOF1	1.439164727	LCN2	-10.50878516	C9orf3	-1.29008174
SDCCAG3	1.436151958	OCLN	-9.840777924	TAPBPL	-1.288696797
RUVBL1	1.425642879	SORT1	-9.396604781	TMEM45A	-1.283328042
ASPHD2	1.424497829	PSG4	-9.214319121	DSC3	-1.282113581
FAM167A	1.424371911	NGFR	-8.842350343	SVIL	-1.280398025
MYBBP1A	1.423392728	AKR1B10	-8.842350343	RIPK4	-1.278855256
ETV5	1.411691236	ANXA10	-8.842350343	TIMP3	-1.276442195
NCAPD3	1.405080375	PCLO	-8.842350343	CERCAM	-1.276197528
DNMT1	1.401398364	RRAD	-8.754887502	AHR	-1.275567426
SLC27A5	1.398549376	SCGB1A1	-8.754887502	ADM	-1.268785327
ODC1	1.397068307	C3orf47	-8.661778098	ARRDC3	-1.266658028
ABCF2	1.393836529	CHI3L2	-8.661778098	ZNF185	-1.264644081
EXOSC9	1.389079709	CYP24A1	-8.562242424	GPR125	-1.261920786
CUTC	1.383490731	P2RY6	-8.562242424	ARHGAP23	-1.258572997
ERRFI1	1.379527623	LOC100132891	-8.562242424	NEDD4L	-1.255058029
CCDC61	1.376375879	PALMD	-8.45532722	EXT1	-1.252722031
LRRC59	1.375153423	AGR2	-8.45532722	MFSD6	-1.251811591
UPP1	1.375153423	CLDN23	-8.45532722	DSC2	-1.250469708
CENPM	1.373458396	HHAT	-8.45532722	PHLDB2	-1.250373022
TARDBP	1.369456465	SDCCAG8	-8.45532722	ST5	-1.246989785
ERGIC1	1.367143828	LTB	-4.767203567	FAM108C1	-1.24610302
PHF15	1.364970045	CLDN1	-4.697239316	SQSTM1	-1.244208747
KIAA0664	1.363036731	C15orf48	-4.414202365	IKBKE	-1.243377446
TUBA1B	1.360055228	DLL1	-4.127755547	TGFB2	-1.239896912
XPO5	1.358448401	CFB	-4.114449291	PPIG	-1.238208136
PFAS	1.357488373	SERPINB13	-4.042774024	LRIG1	-1.238074044
NAT10	1.354541061	TNFSF10	-4.042774024	CHMP4C	-1.236229377
PLK1	1.353775946	CLU	-3.987899862	SLC38A2	-1.234289304
ZNF259	1.352993723	HIST1H2BD	-3.905702703	IL32	-1.233128806
GEMIN5	1.344924293	PDP1	-3.780387874	ETS2	-1.229657175
PAK4	1.344785303	TACSTD2	-3.753787635	KLHL24	-1.226296876
TIMM44	1.337885106	CPA4	-3.722557243	YPEL5	-1.225350849
BIRC3	1.336371637	STARD5	-3.58384888	IL13RA1	-1.223938884
EGR1	1.329680203	EPPK1	-3.564031843	SLU7	-1.212401419
LETM1	1.3237103	PIK3IP1	-3.468371663	FERMT2	-1.211178257
KCNG1	1.323598848	HIST2H2BE	-3.459431619	PTAFR	-1.209729186
ZNF85	1.321928095	SLC2A12	-3.458216717	TMEM212	-1.204523089
PYGB	1.321285065	KRT15	-3.384831229	SCD5	-1.20070429
LSM10	1.317974601	SPRR2D	-3.367509129	CALCOCO1	-1.196568646
SHMT2	1.315638615	FLJ35776	-3.32014589	KIAA1522	-1.194892301
F12	1.314800111	SEMA3C	-3.31251585	SCARA3	-1.192603333
SMOX	1.314777981	LOC100506895	-3.247927513	WDR66	-1.190342544
KCTD5	1.314314911	SLC22A23	-3.221437942	MBNL2	-1.184634098
MCM6	1.311608344	GXYLT2	-3.168935159	HSPB1	-1.183702522
CLN6	1.309328058	CDK18	-3.167944637	CES2	-1.18224938
ZHX3	1.308881203	PTHLH	-3.072998362	TSPYL4	-1.182241066
C16orf53	1.30749961	C1R	-3.019899557	TANK	-1.179249981
FAM65A	1.301616868	FYB	-2.973032952	C1GALT1	-1.174323174
MIS18A	1.297680549	GRHL3	-2.962103392	USP35	-1.173144359
USP5	1.292063454	ISG15	-2.95247163	F3	-1.168985996
TRIB3	1.290566254	CXADR	-2.919557007	TMEM63B	-1.168935159
POLE3	1.287233367	KIAA1199	-2.905702703	CARD10	-1.162650338
WDR76	1.284716169	ST6GALNAC2	-2.901281949	PRKD2	-1.157584126
ORAI1	1.280107919	KAL1	-2.886080408	IFT57	-1.146807591
UBE2S	1.277613666	RRAGC	-2.874469118	MYLK	-1.141949429
PFDN2	1.275675425	GBP2	-2.850511493	STC1	-1.136288623

SNRNP70	1.271626731	CCND2	-2.806400443	CLIP4	-1.133113199
ZFP36L2	1.269930313	TM7SF2	-2.806264037	GOLGA2	-1.128264883
CUL4B	1.269880474	CEL	-2.752907138	CLDN7	-1.122730919
NFS1	1.269460675	SRGN	-2.720845929	BCKDHA	-1.118366715
MCL1	1.267591712	UGCG	-2.654503434	AQP3	-1.116104067
KRT81	1.266511978	EFNA1	-2.653005049	KLF3	-1.11599814
CYP27B1	1.266254497	TMPRSS3	-2.642430602	C10orf58	-1.113981351
DIMT1L	1.265388824	DAAM1	-2.633583033	TNFAIP8	-1.111744499
SLC35B4	1.261273948	C5orf4	-2.630543535	AFTPH	-1.106957611
PNO1	1.258473978	S100A7	-2.605859842	RAPGEF1	-1.10581412
PUS1	1.256223171	DENND1B	-2.584962501	YTHDC1	-1.102722191
TUBB	1.252792402	MYO5B	-2.584962501	C10orf32	-1.102564161
C12orf11	1.247092862	PLD1	-2.584220183	NGFRAP1	-1.098454755
SH3KBP1	1.242890375	RND3	-2.58384888	TSPAN1	-1.095472997
DDX39A	1.240048027	GBP1	-2.583477483	FRMD6	-1.091409191
PNP	1.239910534	AMIGO2	-2.536577258	ZNF561	-1.08809133
WDR74	1.238885969	C5orf56	-2.513236725	DSE	-1.081445217
C12orf24	1.237440224	FBXO32	-2.488846534	C5orf32	-1.077718097
WDR34	1.235857145	HIST1H1C	-2.413366746	FAP	-1.076633964
CCDC58	1.233428655	ZNF385A	-2.400653581	CORO2A	-1.076595691
ARMC6	1.229212739	RNASE7	-2.349449158	UBL3	-1.074493726
FANCA	1.224372785	ARRDC4	-2.321105812	DUSP16	-1.073687907
DTL	1.223260312	SAA2	-2.320740203	MRC2	-1.069116549
SLC25A28	1.217890327	IFI27	-2.320591647	EPB49	-1.068514735
FARSA	1.213027003	CD24	-2.282764648	IL1RAP	-1.065095028
RPF2	1.212910926	SNCAIP	-2.278535499	CDH3	-1.061804439
POLR3K	1.212340772	DQX1	-2.278535499	CD9	-1.059300844
WDR18	1.211631529	CLIC3	-2.271174794	HEG1	-1.05782463
TUBA1C	1.209951895	C1orf74	-2.271174794	CALD1	-1.054827443
ZNF622	1.209531503	MIR210HG	-2.249381404	TMTC3	-1.054139335
DEAF1	1.207821609	PRSS23	-2.243859615	FCHO2	-1.050581133
RGL2	1.206916895	PPL	-2.218297343	HSP90B1	-1.046090065
RAD51	1.20515349	C14orf45	-2.211945634	COX6C	-1.043682153
MPRIP	1.202679882	ARID5B	-2.200471803	RUNX1	-1.043314258
HELLS	1.201382433	DST	-2.197221077	PGRMC2	-1.042718464
FSTL3	1.20123143	FBN1	-2.193787111	FLJ36031	-1.042590187
TUBB2C	1.197568403	ENTPD7	-2.150666646	NUPR1	-1.036940788
C1QBP	1.195308615	OTUD1	-2.130082475	SERPINE2	-1.0359128
NCRNA00116	1.194816177	KRT17	-2.104330961	RAB13	-1.032400966
SSU72	1.192583758	ULBP2	-2.085890421	GDF15	-1.030546802
TACC3	1.186255229	SLFN12	-2.085890421	SLC9A1	-1.030324537
PROCR	1.181760359	GPR56	-2.075203368	SCPEP1	-1.029985009
GOT1	1.181236188	DRAM1	-2.072190512	ZMIZ1	-1.02772883
MTERFD1	1.180896119	LRP1	-2.062968279	SPATS2L	-1.026678532
UBIAD1	1.174340292	SYNPO	-2.060286924	PI4K2A	-1.022770008
PYCR1	1.171261449	FAM134B	-2.053457942	C6orf120	-1.022532213
IMPDH1	1.170849002	MYL9	-2.045501559	PKD1	-1.019899557
RAPH1	1.170828905	FAAH2	-2.042774024	DHRS3	-1.016985889
VPS4A	1.169094195	TPM1	-2.041537949	GJB2	-1.015815715
MED24	1.168926384	PLOD2	-2.040618064	LRPAP1	-1.01573674
FTSJ1	1.167456746	FXYD3	-2.020547964	MLL5	-1.01544987
FERMT1	1.165543584	CLCA2	-1.992346427	HINT3	-1.013037275
RRM2	1.165488059	ITGAV	-1.983613527	UBE2H	-1.004546874
EIF3I	1.162402694	NTN4	-1.973032952	BLOC1S2	-1.003495133
CEBPB	1.162135501	PLK2	-1.966211687		
NOLC1	1.16080051	TNC	-1.965589969		
GNL3	1.160454692	PPP1R3B	-1.958594483		
CDCA4	1.157921154	VAV3	-1.957830546		
TWF2	1.157646672	NDRG1	-1.954734343		
NUP88	1.155866342	BDKRB2	-1.94858947		

Supplementary Table 2

The interactions between AURKA regulated differentially expressed genes are shown.

Node1	Node2	Neighborhood	Fusion	Cooccurence	Homology	Coexpression	Experimental	Knowledge	Textmining	Combined_score
ABLIM3	RAC3	0	0	0	0	0	0	0.8	0	0.8
ACOT7	UBIAD1	0	0	0	0	0	0	0.9	0	0.899
ADAM8	SVIL	0	0	0	0	0	0	0.72	0	0.72
AGR2	AREG	0	0	0	0	0	0	0	0.833	0.833
AHR	CYP24A1	0	0	0	0	0	0	0	0.8	0.8
ALG3	RFT1	0	0	0	0	0	0	0.9	0.088	0.902
ANGPTL4	MED8	0	0	0	0	0	0	0.9	0	0.899
AOX1	MAOA	0	0	0	0	0	0	0.9	0.275	0.922
ATRIP	CDC45	0	0	0	0	0	0	0.9	0.509	0.947
ATRIP	MCM6	0	0	0	0	0	0	0.9	0.236	0.918
ATRIP	MCM5	0	0	0	0	0.119	0	0.9	0.421	0.941
ATRIP	CDC6	0	0	0	0	0	0.621	0.9	0.262	0.968
AURKB	CDC6	0	0	0	0	0.678	0	0	0.341	0.773
AURKB	CENPK	0	0	0	0	0.354	0	0.9	0.103	0.933
AURKB	RRM2	0	0	0	0	0.772	0	0	0.215	0.808
AURKB	GINS2	0	0	0	0	0.7	0	0	0.113	0.716
AURKB	PLK1	0	0	0	0.824	0.747	0	0.9	0.784	0.976
AURKB	NCAPH	0	0	0	0	0.69	0.62	0.9	0.129	0.987
AURKB	CENPM	0	0	0	0	0.772	0	0.9	0	0.975
AURKB	CHMP4C	0	0	0	0	0	0	0	0.806	0.806
AURKB	CDC45	0	0	0	0	0.796	0	0	0.262	0.839
AURKB	CDCA5	0	0	0	0	0.772	0	0	0.286	0.826
AURKB	NUF2	0	0	0	0	0.611	0.62	0.9	0.532	0.991
AURKB	TRIP13	0	0	0	0	0.711	0	0	0.136	0.734
AURKB	MYBL2	0	0	0	0	0.723	0	0	0.144	0.746
AURKB	EXO1	0	0	0	0	0.722	0	0	0	0.722
BDKRB2	PTAFR	0	0	0	0.8	0	0	0.9	0.196	0.902
BDKRB2	PIK3R1	0	0	0	0	0	0	0.9	0.082	0.902
BDKRB2	ANXA1	0	0	0	0	0	0	0.9	0.065	0.9
BDNF	EIF4EBP1	0	0	0	0	0	0	0	0.831	0.831
BDNF	DDR1	0	0	0	0	0	0	0	0.81	0.81
BDNF	EHD4	0	0	0	0	0	0	0.9	0	0.899
BDNF	FOS	0	0	0	0	0	0	0	0.929	0.929
BDNF	NTF4	0	0	0	0.905	0	0.9	0	0.87	0.908
BDNF	TPM1	0	0	0	0	0	0	0	0.917	0.917
BDNF	SORT1	0	0	0	0	0	0.846	0.9	0.43	0.99
BDNF	NGFR	0	0	0	0	0	0	0.9	0.968	0.996
BIRC3	CSF3	0	0	0	0	0	0	0	0.835	0.835
BLOC1S2	IFT57	0	0	0	0	0	0.621	0	0.292	0.714
BNIP3L	IKBKE	0	0	0	0	0	0	0	0.808	0.808
BOP1	NIP7	0	0	0	0	0.683	0.346	0	0.148	0.799
BOP1	BYSL	0	0	0	0	0.779	0	0	0.095	0.787
BOP1	NAT10	0	0	0	0	0.738	0	0	0	0.738
BRIX1	POLR2E	0	0	0	0	0.759	0	0	0	0.759
BRIX1	BOP1	0	0	0	0	0.617	0.323	0	0.226	0.771
BRIX1	PNO1	0	0	0	0	0.882	0	0	0	0.882
BRIX1	SNRPD1	0	0	0	0	0.785	0	0	0	0.786
BRIX1	BYSL	0	0	0	0	0.85	0	0	0	0.85
BRIX1	DIMT1L	0	0	0	0	0.86	0	0	0	0.86
BRIX1	SNRPA1	0	0	0	0	0.821	0	0	0	0.821
BRIX1	NOC4L	0	0	0	0	0.718	0	0	0	0.718
BRIX1	POLR1B	0	0	0	0	0.734	0	0	0	0.734
BRIX1	NIP7	0	0	0	0	0.902	0.64	0	0.071	0.963
BRIX1	EMG1	0	0	0	0	0.837	0	0	0	0.837
BRIX1	NAT10	0	0	0	0	0.788	0	0	0	0.788
BRIX1	NUP155	0	0	0	0	0.776	0	0	0	0.776
BRIX1	DDX56	0	0	0	0	0.563	0.3	0	0.22	0.728
BRIX1	TBL3	0	0	0	0	0.708	0	0	0	0.708
BYSL	DIMT1L	0	0	0	0	0.804	0.7	0	0.25	0.95
C13orf15	RUNX1	0	0	0	0	0	0	0	0.817	0.817
C21orf45	CENPK	0	0	0	0	0.388	0	0.9	0	0.934
C5orf4	SCARA3	0	0	0	0	0	0	0	0.715	0.714
C5orf4	TM7SF2	0	0	0	0	0	0	0.9	0	0.899
CA2	GDF15	0	0	0	0	0	0	0	0.829	0.829
CA2	SLC9A1	0	0	0	0	0	0.846	0	0.218	0.872
CAD	PPAT	0.247	0	0	0	0.207	0	0.9	0.543	0.966
CAD	ASNS	0	0	0	0	0.161	0	0.9	0	0.91
CALD1	MYLK	0	0	0	0	0.405	0	0.9	0.947	0.996
CALD1	TPM1	0	0	0	0	0.3	0	0.9	0.954	0.996
CCL20	BDKRB2	0	0	0	0	0	0	0.9	0	0.899
CCL20	ANXA1	0	0	0	0	0	0	0.9	0.068	0.9
CCND2	NGFR	0	0	0	0	0	0	0	0.813	0.813
CCND2	CDK4	0	0	0	0	0	0.999	0.9	0.954	0.999
CCND3	MED8	0	0	0	0	0	0	0.9	0	0.899
CCND3	MED24	0	0	0	0	0	0	0.9	0	0.899
CCND3	RUNX1	0	0	0	0	0	0.621	0	0.341	0.733
CCND3	NOTCH1	0	0	0	0	0	0	0	0.854	0.854
CCND3	PIK3R1	0	0	0	0	0	0	0.9	0.105	0.904
CCND3	CDK4	0	0	0	0	0	0.999	0.9	0.955	0.999
CCND3	E2F1	0	0	0	0	0	0	0.9	0.341	0.929
CCND3	MCM10	0	0	0	0	0	0.925	0	0	0.925
CCND3	CCND2	0	0	0	0.95	0	0	0.9	0.84	0.904
CDA	UPP1	0.426	0	0	0	0	0	0.9	0.499	0.967
CDA	PNP	0.797	0	0	0	0	0	0.9	0.592	0.99
CDC45	MYBL2	0	0	0	0	0.782	0	0	0.108	0.793
CDC45	HELLS	0	0	0	0	0.82	0	0	0.071	0.822

Node1	Node2	Neighborhood	Fusion	Cooccurence	Homology	Coexpression	Experimental	Knowledge	Textmining	Combined_score
CDC45	CENPK	0	0	0	0	0.807	0	0	0.244	0.845
CDC45	TRIP13	0	0	0	0	0.912	0	0	0.18	0.923
CDC45	CDC6	0	0	0	0	0.946	0	0.9	0.752	0.998
CDC45	GINS2	0	0	0	0	0.922	0.389	0.9	0.228	0.995
CDC45	RNASEH2A	0	0	0	0	0.841	0	0	0.164	0.859
CDC45	NUP155	0	0	0	0	0.711	0	0	0.103	0.723
CDC45	CENPM	0	0	0	0	0.868	0	0	0.126	0.877
CDC45	CDK4	0	0	0	0	0.224	0.926	0	0.318	0.955
CDC45	MCM5	0	0	0	0	0.838	0.492	0.9	0.927	0.999
CDC45	NCAPH	0	0	0	0	0.892	0	0	0	0.892
CDC6	TRIP13	0	0	0	0	0.884	0	0	0.125	0.892
CDCA5	HELLS	0	0	0	0	0.706	0	0	0	0.706
CDCA5	CDC6	0	0	0	0	0.91	0	0	0.317	0.934
CDCA5	GINS2	0	0	0	0	0.88	0	0	0.128	0.889
CDCA5	MCM6	0	0	0	0	0.781	0	0	0.229	0.82
CDCA5	CENPM	0	0	0	0	0.876	0	0	0.086	0.879
CDCA5	CENPK	0	0	0	0	0.788	0	0	0.271	0.835
CDCA5	NCAPH	0	0	0	0	0.85	0	0	0.173	0.868
CDCA5	NUF2	0	0	0	0	0.919	0	0	0.145	0.926
CDCA5	TRIP13	0	0	0	0	0.846	0	0	0.261	0.879
CDCA5	RAD51	0	0	0	0	0.814	0	0	0.077	0.817
CDCA5	CDC45	0	0	0	0	0.923	0	0	0.196	0.934
CDH1	SNAI2	0	0	0	0	0	0	0	0.907	0.907
CDH1	CDC6	0	0	0	0	0	0	0	0.824	0.824
CDH3	CDH1	0	0	0	0.945	0.275	0.619	0	0.584	0.714
CDK4	CDC6	0	0	0	0	0.199	0.389	0	0.514	0.729
CDK4	JUND	0	0	0	0	0	0	0.9	0.842	0.983
CDK4	AHR	0	0	0	0	0	0	0	0.847	0.847
CEBPB	HSP90B1	0	0	0	0	0	0	0.9	0	0.899
CEBPB	RUNX1	0	0	0	0	0	0.621	0	0.261	0.701
CEBPB	EGR1	0	0	0	0	0	0.621	0	0.261	0.701
CEBPB	MED8	0	0	0	0	0	0	0.9	0	0.899
CEL	MGLL	0	0	0	0	0	0	0.9	0.148	0.909
CELSR2	SORT1	0	0	0	0	0	0	0	0.734	0.734
CENPK	CENPM	0	0	0	0	0.538	0	0.9	0.379	0.967
CENPK	CDC6	0	0	0	0	0.782	0	0	0.122	0.796
CENPK	TRIP13	0	0	0	0	0.741	0	0	0.32	0.812
CENPK	HELLS	0	0	0	0	0.788	0	0	0.075	0.791
CENPM	CDC6	0	0	0	0	0.798	0	0	0	0.798
CENPM	TRIP13	0	0	0	0	0.765	0	0	0.164	0.791
CHMP4C	VPS4A	0	0	0	0	0	0.586	0.9	0.462	0.974
CLDN1	CD9	0	0	0	0	0	0	0	0.817	0.817
CLDN7	CD9	0	0	0	0	0	0	0	0.846	0.846
CLDN7	CLDN1	0	0	0	0.953	0	0	0.8	0.786	0.807
CLDN7	CDH1	0	0	0	0	0.43	0	0	0.896	0.936
CLDN7	OCLN	0	0	0	0	0	0	0.8	0.66	0.927
CLU	SPARC	0	0	0	0	0.069	0	0.9	0.24	0.919
CLU	SRGN	0	0	0	0	0	0	0.9	0.122	0.906
CSNK2A1	FKBP4	0	0	0	0	0	0.621	0	0.823	0.928
CUL4B	DTL	0	0	0	0	0	0.663	0.9	0.426	0.978
CUL4B	AHR	0	0	0	0	0	0.62	0	0.627	0.848
CYFIP2	RAC3	0	0	0	0	0	0	0.8	0.551	0.904
DDR1	NOTCH1	0	0	0	0	0	0	0	0.811	0.811
DDR1	BTF3L4	0	0	0	0	0	0	0.9	0	0.899
DDR1	ACOT7	0	0	0	0	0	0	0.9	0	0.899
DDR1	EPHB3	0	0	0	0	0	0	0	0.822	0.822
DENND1B	NUF2	0	0	0	0	0	0	0.9	0	0.899
DEPDC1	DTL	0	0	0	0	0.778	0	0	0	0.778
DEPDC1	RRM2	0	0	0	0	0.734	0	0	0.104	0.746
DEPDC1	NUF2	0	0	0	0	0.873	0	0	0.121	0.881
DEPDC1	UBE2T	0	0	0	0	0.73	0	0	0	0.73
DEPDC1	CDCA5	0	0	0	0	0.787	0	0	0	0.787
DEPDC1	CDC45	0	0	0	0	0.761	0	0	0	0.761
DEPDC1	PRC1	0	0	0	0	0.7	0	0	0	0.7
DEPDC1	CENPK	0	0	0	0	0.766	0	0	0	0.767
DEPDC1	KIF2C	0	0	0	0	0.758	0	0	0.102	0.768
DEPDC1	CDC6	0	0	0	0	0.748	0	0	0.076	0.752
DEPDC1	TRIP13	0	0	0	0	0.72	0	0	0	0.72
DEPDC1	EXO1	0	0	0	0	0.79	0	0	0.081	0.794
DEPDC1	MCM10	0	0	0	0	0.713	0	0	0	0.712
DLL1	HES7	0	0	0	0	0	0	0	0.902	0.902
DLL1	NOTCH1	0	0	0	0.811	0	0.62	0.9	0.968	0.966
DLL1	DLG1	0	0	0	0	0	0	0	0.8	0.8
DNER	NOTCH1	0	0	0	0.828	0	0	0.9	0.38	0.905
DNMT1	RUNX1	0	0	0	0	0	0.621	0	0.275	0.706
DNMT1	HELLS	0	0	0	0	0.467	0.62	0	0.851	0.965
DNMT1	MCM6	0	0	0	0	0.694	0	0	0.108	0.709
DNMT1	FOS	0	0	0	0	0	0	0	0.859	0.859
DNMT1	E2F1	0	0	0	0	0.105	0.619	0.9	0.146	0.964
DSC3	JUP	0	0	0	0	0	0.621	0	0.563	0.823
DSE	CSGALNACT1	0	0	0	0	0	0	0.9	0.086	0.902
DST	KRT14	0	0	0	0	0	0.621	0	0.402	0.758
DTL	NCAPH	0	0	0	0	0.853	0	0	0	0.853
DTL	EXO1	0	0	0	0	0.938	0	0	0.071	0.939
DTL	MCM10	0	0	0	0	0.871	0	0	0.163	0.885
DTL	NUF2	0	0	0	0	0.888	0	0	0.07	0.889

Node1	Node2	Neighborhood	Fusion	Cooccurence	Homology	Coexpression	Experimental	Knowledge	Textmining	Combined_score
DTL	CDC45	0	0	0	0	0.945	0	0	0.162	0.951
DTL	MCM6	0	0	0	0	0.82	0	0	0.11	0.829
DTL	CDC6	0	0	0	0	0.948	0	0	0.29	0.96
DTL	HELLS	0	0	0	0	0.857	0	0	0	0.857
DTL	CDCA5	0	0	0	0	0.874	0	0	0.086	0.877
DTL	RRM2	0	0	0	0	0.906	0	0	0.092	0.909
DTL	CENPK	0	0	0	0	0.86	0	0	0	0.86
DTL	TRIP13	0	0	0	0	0.849	0	0	0.133	0.861
DTL	RAD51	0	0	0	0	0.849	0	0	0.072	0.851
DTL	CENPM	0	0	0	0	0.797	0	0	0	0.797
DTL	GINS2	0	0	0	0	0.872	0	0	0.126	0.881
DTYMK	CDC6	0	0	0	0	0.711	0	0	0.252	0.769
DTYMK	RNASEH2A	0	0	0	0	0.713	0	0	0.097	0.724
DTYMK	MCM6	0	0	0	0	0.726	0	0	0.065	0.727
DTYMK	GINS2	0	0	0	0	0.694	0	0	0.137	0.718
DTYMK	MCM5	0	0	0	0	0.708	0	0	0.102	0.72
DTYMK	CDC45	0	0	0	0	0.784	0	0	0.086	0.79
DTYMK	RRM2	0	0	0	0	0.759	0	0.9	0.297	0.98
DUSP4	EGR1	0	0	0	0	0	0	0	0.85	0.85
E2F1	CCND2	0	0	0	0	0	0	0.9	0.341	0.929
E2F1	CDK4	0	0	0	0	0.133	0	0.9	0.564	0.956
E2F1	NOTCH1	0	0	0	0	0	0	0.9	0.221	0.916
E2F1	MYBL2	0	0	0	0	0.5	0.588	0.9	0.305	0.982
E2F1	NGFR	0	0	0	0	0	0	0.9	0.087	0.902
E2F1	SORT1	0	0	0	0	0	0	0.9	0	0.899
E2F1	CDC45	0	0	0	0	0.654	0	0.9	0.103	0.964
E2F1	CDC6	0	0	0	0	0.464	0	0.9	0.425	0.964
E2F1	RRM2	0	0	0	0	0.38	0	0.9	0.829	0.987
EFNA1	PIK3R1	0	0	0	0	0	0	0.9	0	0.899
EGR1	CSF3	0	0	0	0	0	0	0	0.832	0.832
EGR1	CSNK2A1	0	0	0	0	0	0.621	0	0.803	0.92
EGR1	DUSP1	0	0	0	0	0.545	0	0	0.851	0.928
EIF3B	GTPBP4	0	0	0	0	0.743	0	0	0	0.744
EIF3I	GTPBP4	0	0	0	0	0.708	0	0	0	0.708
EIF3I	NIP7	0	0	0	0	0.721	0	0	0	0.721
EIF3I	EIF3B	0	0	0	0	0.651	0.984	0.9	0.412	0.999
EIF4EBP1	LRPAP1	0	0	0	0	0	0.62	0	0.395	0.754
EMG1	NIP7	0	0	0	0	0.855	0	0	0.079	0.858
EMG1	BYSL	0	0	0	0	0.819	0.586	0	0.424	0.951
EMG1	C1QBP	0	0	0	0	0.755	0	0	0	0.755
EMG1	NAT10	0	0	0	0	0.76	0.644	0	0.479	0.949
EMG1	POLR2E	0	0	0.237	0	0.762	0	0	0	0.806
EMG1	DIMT1L	0	0	0	0	0.727	0	0	0.089	0.734
EPHB3	EFNB2	0	0	0	0	0	0	0.9	0.784	0.976
EPHB3	SOX9	0	0	0	0	0	0	0	0.842	0.842
EREG	AHR	0	0	0	0	0	0	0	0.818	0.818
ETS2	FOS	0	0	0	0	0	0.619	0	0.341	0.732
EXO1	CENPM	0	0	0	0	0.784	0	0	0	0.784
EXO1	HELLS	0	0	0	0	0.838	0	0	0	0.839
EXO1	MCM6	0	0.002	0	0	0.837	0	0	0.086	0.841
EXO1	GINS2	0	0	0	0	0.803	0	0	0.087	0.808
EXO1	CENPK	0	0	0	0	0.785	0	0	0	0.785
EXO1	NUP155	0	0	0	0	0.709	0	0	0	0.709
EXO1	NUF2	0	0	0	0	0.884	0	0	0	0.885
EXO1	RAD51	0	0	0	0	0.843	0	0	0.613	0.935
EXO1	CDCA5	0	0	0	0	0.888	0	0	0.163	0.9
EXO1	RRM2	0	0	0	0	0.858	0	0	0.111	0.866
EXO1	CDC6	0	0	0	0	0.913	0	0	0.162	0.922
EXO1	TRIP13	0	0	0	0	0.89	0	0	0.104	0.895
EXO1	NCAPH	0	0	0	0	0.851	0	0	0.146	0.864
EXO1	CDC45	0	0	0	0	0.942	0	0	0.126	0.946
EXOSC4	BYSL	0	0	0	0	0.63	0	0	0.348	0.743
EXOSC4	NIP7	0	0	0	0	0.632	0	0	0.34	0.741
EXOSC4	ZFP36	0	0	0	0	0	0	0.9	0	0.899
EXOSC9	ZFP36	0	0	0	0	0	0	0.9	0	0.899
EXOSC9	EXOSC4	0.322	0	0.484	0	0.104	0.998	0.9	0.212	0.999
F12	C1QBP	0	0	0	0	0	0	0.9	0.514	0.948
F2R	PROCR	0	0	0	0	0	0.621	0	0.79	0.915
F2R	P2RY6	0	0	0	0.793	0	0	0.9	0.129	0.901
F2R	ANXA1	0	0	0	0	0	0	0.9	0.101	0.904
F2R	PTAFR	0	0	0	0.792	0	0	0.9	0.241	0.903
F2R	EGR1	0	0	0	0	0	0	0	0.828	0.828
F2R	BDKRB2	0	0	0	0.741	0	0	0.9	0.464	0.911
F2R	PIK3R1	0	0	0	0	0	0	0.9	0	0.899
F2R	CDH1	0	0	0	0	0	0	0	0.838	0.838
F3	F12	0	0	0	0	0	0	0	0.752	0.752
F3	EGR1	0	0	0	0	0	0	0	0.902	0.902
FANCA	EXO1	0	0	0	0	0.684	0	0	0.264	0.751
FANCA	ATRIP	0	0	0	0	0	0	0.9	0.167	0.911
FANCA	RAD51	0	0	0	0	0.613	0	0	0.61	0.839
FANCA	CDC45	0	0	0	0	0.738	0	0	0.264	0.794
FANCA	UBE2T	0	0	0	0	0.457	0	0.9	0.319	0.957
FARSA	POLR2E	0.253	0	0	0	0.641	0	0	0	0.714
FARSA	MRPL4	0	0	0	0	0.689	0	0	0.275	0.76
FARSA	MARS	0	0	0.222	0	0.515	0	0	0.81	0.918
FARSA	MRPS2	0	0	0	0	0.603	0	0	0.364	0.731

Node1	Node2	Neighborhood	Fusion	Cooccurence	Homology	Coexpression	Experimental	Knowledge	Textmining	Combined_score
GTPBP4	DIMT1L	0	0	0	0	0.751	0	0	0.102	0.761
GTPBP4	NIP7	0	0	0	0	0.829	0.66	0	0.144	0.943
GTPBP4	BYSL	0	0	0	0	0.795	0	0	0	0.795
GTPBP4	POLR1B	0	0	0	0	0.704	0	0	0	0.704
GTPBP4	PNO1	0	0	0	0	0.838	0	0	0	0.839
H2AFX	RAD51	0	0	0	0	0.285	0.846	0	0.752	0.969
H2AFX	AURKB	0	0	0	0	0.597	0	0	0.341	0.716
H2AFX	HIST2H2BE	0	0	0	0	0	0.834	0	0	0.834
H2AFX	EXO1	0	0	0	0	0.366	0.619	0	0.376	0.828
HCFC1	MLL5	0	0	0	0	0	0.067	0.9	0	0.9
HELLS	CDC6	0	0	0	0	0.833	0	0	0.138	0.846
HIPK2	MT2A	0	0	0	0	0	0	0	0.818	0.818
HNRNPAB	TP63	0	0	0	0	0	0.789	0	0.146	0.807
HSP90B1	LRP1	0	0	0	0	0	0.621	0	0.907	0.962
IARS	YARS	0	0	0	0	0.729	0	0	0.858	0.959
IARS	MARS	0	0	0.419	0	0.763	0.418	0.9	0.925	0.999
IARS	FARSA	0	0	0	0	0.488	0.538	0	0.784	0.942
IARS	SHMT2	0	0	0	0	0.313	0	0	0.646	0.741
IARS	RPL11	0.224	0	0	0	0.584	0	0	0.192	0.703
IARS	BRIX1	0	0	0	0	0.741	0	0	0	0.741
IARS	ASNS	0	0	0	0	0.718	0	0	0.12	0.735
ID1	CEBPB	0	0	0	0	0	0	0.9	0.122	0.906
ID1	NOTCH1	0	0	0	0	0	0	0	0.841	0.841
IFI27	EGR1	0	0	0	0	0	0	0.9	0.068	0.9
IFI6	IFI27	0	0	0	0.884	0.824	0	0.9	0.8	0.982
IFI6	EGR1	0	0	0	0	0	0	0.9	0	0.899
IKBKE	TANK	0	0	0	0	0	0.975	0.9	0.902	0.999
ILF2	MCM5	0	0	0	0	0.413	0.8	0	0.125	0.883
ILF2	RUVBL1	0	0	0	0	0.588	0.8	0	0	0.912
ILF2	SNRPD1	0	0	0	0	0.757	0	0	0	0.757
ILF2	C1QBP	0	0	0	0	0.74	0	0	0	0.74
ILF2	MCM6	0	0	0	0	0.404	0.8	0	0	0.872
ILF2	SHMT2	0	0	0	0	0.269	0.8	0	0.082	0.847
IMPDH1	PPAT	0.205	0	0	0	0.236	0	0	0.631	0.745
IRF6	EGR1	0	0	0	0	0	0	0.9	0	0.899
IRF6	IFI6	0	0	0	0	0	0	0.9	0	0.899
IRF6	FOS	0	0	0	0	0	0	0	0.833	0.833
IRF6	IFI27	0	0	0	0	0	0	0.9	0	0.899
IRF6	PTAFR	0	0	0	0	0	0	0.9	0.085	0.902
IscS	FDX1	0.612	0	0	0	0.18	0.282	0	0.805	0.946
ISG15	IFI27	0	0	0	0	0.871	0	0.9	0.43	0.991
ISG15	IRF6	0	0	0	0	0	0	0.9	0.133	0.907
ISG15	GBP2	0	0	0	0	0.107	0	0.9	0.374	0.936
ISG15	EGR1	0	0	0	0	0	0	0.9	0.086	0.902
ISG15	GBP1	0	0	0	0	0.448	0	0	0.507	0.71
ISG15	IFI6	0	0	0	0	0.915	0	0.9	0.43	0.994
ITGA10	ITGAV	0	0	0	0.677	0	0	0.72	0.175	0.73
ITGA10	CSK	0	0	0	0	0	0	0.8	0	0.8
ITGA10	TNC	0	0	0	0	0	0	0.8	0	0.8
ITGAV	MMP2	0	0	0	0	0	0.846	0	0.427	0.906
ITGAV	CSK	0	0	0	0	0	0	0.8	0	0.8
JUP	DSC2	0	0	0	0	0	0.621	0	0.67	0.866
JUP	SOX4	0	0	0	0	0	0	0	0.802	0.802
JUP	PIK3R1	0	0	0	0	0	0	0.9	0	0.899
JUP	CDH1	0	0	0	0	0.109	0.995	0.72	0.954	0.999
JUP	DSG3	0	0	0	0	0	0.621	0	0.751	0.899
KIF2C	CDCA5	0	0	0	0	0.892	0	0	0.241	0.912
KIF2C	RAD51	0	0	0	0	0.793	0	0	0	0.793
KIF2C	AURKB	0	0	0	0	0.859	0	0.9	0.93	0.998
KIF2C	RNASEH2A	0	0	0	0	0.72	0	0	0	0.72
KIF2C	UBE2T	0	0	0	0	0.798	0	0	0	0.798
KIF2C	TRIP13	0	0	0	0	0.842	0	0	0.268	0.876
KIF2C	NUF2	0	0	0	0	0.741	0	0.9	0.38	0.981
KIF2C	NCAPH	0	0	0	0	0.82	0	0	0.288	0.863
KIF2C	CENPM	0	0	0	0	0.748	0	0.9	0	0.973
KIF2C	RRM2	0	0	0	0	0.863	0	0	0.216	0.885
KIF2C	GINS2	0	0	0	0	0.791	0	0	0.169	0.815
KIF2C	CDC45	0	0	0	0	0.903	0	0	0.118	0.909
KIF2C	PLK1	0	0	0	0	0.833	0	0.9	0.877	0.997
KIF2C	EXO1	0	0	0	0	0.836	0	0	0	0.836
KIF2C	NUDC	0	0	0	0	0.114	0	0.9	0	0.905
KIF2C	DTL	0	0	0	0	0.818	0	0	0.108	0.827
KIF2C	CENPK	0	0	0	0	0.512	0	0.9	0.309	0.961
KIF2C	MCM10	0	0	0	0	0.837	0	0	0.22	0.864
KIF2C	CDC6	0	0	0	0	0.79	0	0	0.244	0.831
KPNA2	RRM2	0	0	0	0	0.721	0	0	0.216	0.766
KRT6A	KRT17	0	0	0	0.813	0.295	0.538	0	0.79	0.702
LCN2	MMP2	0	0	0	0	0	0.621	0	0.341	0.733
LRPAP1	SORT1	0	0	0	0	0	0.846	0	0	0.846
LRPAP1	LRP1	0	0	0	0	0	0.983	0	0.427	0.989
LYAR	NIP7	0	0	0	0	0.71	0	0	0.127	0.73
LYAR	BRIX1	0	0	0	0	0.83	0.175	0	0.201	0.872
MAFB	FOS	0	0	0	0	0	0.096	0	0.867	0.872
MAK16	BRIX1	0	0	0	0	0.869	0	0	0	0.869
MAK16	NIP7	0	0	0	0	0.85	0	0	0	0.85
MAK16	BYSL	0	0	0	0	0.814	0	0	0.105	0.822

Node1	Node2	Neighborhood	Fusion	Cooccurence	Homology	Coexpression	Experimental	Knowledge	Textmining	Combined_score
MAK16	TBL3	0	0	0	0	0.71	0	0	0	0.71
MAK16	NAT10	0	0	0	0	0.801	0	0	0.31	0.854
MAK16	EMG1	0	0	0	0	0.776	0	0	0.113	0.788
MAK16	DIMT1L	0	0	0	0	0.818	0	0	0.181	0.841
MAK16	PNO1	0	0	0	0	0.889	0	0	0.228	0.909
MAOA	ALDH3A1	0	0	0	0	0	0	0.9	0.072	0.9
MAOA	ALDH3B1	0	0	0	0	0	0	0.9	0.068	0.9
MARCKS	RIPK4	0	0	0	0	0	0	0	0.859	0.859
MARS	DIMT1L	0.172	0	0	0	0.308	0	0	0.627	0.756
MCL1	E2F1	0	0	0	0	0	0	0.9	0	0.899
MCL1	ELF3	0	0	0	0	0	0	0	0.751	0.75
MCL1	LRPAP1	0	0	0	0	0	0	0	0.864	0.864
MCM10	CENPK	0	0	0	0	0.707	0	0	0.202	0.75
MCM10	GINS2	0	0	0	0	0.728	0	0	0.064	0.729
MCM10	HELLS	0	0	0	0	0.755	0	0	0.133	0.773
MCM10	MCM5	0	0	0	0	0.548	0	0.9	0.54	0.976
MCM10	CDC45	0	0	0	0	0.907	0.923	0.9	0.807	0.999
MCM10	SIRT2	0	0	0	0	0	0	0	0.817	0.817
MCM10	RAD51	0	0	0	0	0.807	0	0	0.112	0.817
MCM10	CDCA5	0	0	0	0	0.794	0	0	0.2	0.825
MCM10	MCM6	0	0	0	0	0.655	0.974	0.9	0.675	0.999
MCM10	CDC6	0	0	0	0	0.86	0.917	0.9	0.562	0.999
MCM10	TRIP13	0	0	0	0	0.721	0	0	0.147	0.746
MCM10	EXO1	0	0	0	0	0.871	0	0	0	0.871
MCM10	NCAPH	0	0	0	0	0.756	0	0	0.126	0.773
MCM10	NUF2	0	0	0	0	0.783	0	0	0.183	0.81
MCM10	CCND2	0	0	0	0	0	0.925	0	0	0.925
MCM10	ATRIP	0	0	0	0	0	0	0.9	0.331	0.928
MCM10	RRM2	0	0	0	0	0.793	0	0	0.157	0.814
MCM5	CDC6	0	0	0	0	0.743	0.098	0.9	0.7	0.991
MCM5	CENPM	0	0	0	0	0.745	0	0	0	0.745
MCM5	TRIP13	0	0	0	0	0.752	0	0	0.161	0.778
MCM6	GINS2	0	0	0	0	0.826	0.379	0.9	0.107	0.988
MCM6	CENPK	0	0	0	0	0.702	0	0	0	0.702
MCM6	CDC45	0	0	0	0	0.893	0.987	0.9	0.66	0.999
MCM6	TRIP13	0	0	0	0	0.802	0	0	0.167	0.824
MCM6	HELLS	0	0	0	0	0.748	0	0	0.104	0.759
MCM6	RNASEH2A	0	0	0	0	0.686	0	0	0.183	0.726
MCM6	CDC6	0	0	0	0	0.863	0.127	0.9	0.585	0.994
MCM6	MCM5	0	0	0.52	0.822	0.817	0.928	0.9	0.752	0.998
MED24	CEBPB	0	0	0	0	0	0	0.9	0	0.899
MED24	MED8	0	0	0	0	0	0	0.9	0.22	0.916
MED24	CDK4	0	0	0	0	0	0	0.9	0	0.899
MED24	ANGPTL4	0	0	0	0	0	0	0.9	0	0.899
MED8	CDK4	0	0	0	0	0	0	0.9	0	0.899
MMP2	CD9	0	0	0	0	0	0	0	0.835	0.835
MRPL12	MRPL4	0.58	0	0	0	0.793	0.604	0	0.799	0.991
MRPL12	SHMT2	0	0	0	0	0.368	0	0	0.555	0.7
MRPL12	MRPS2	0	0	0	0	0.658	0.227	0	0.829	0.948
MRPL4	MRPS2	0.147	0	0	0	0.666	0.079	0	0.8	0.936
MTHFD2	SHMT2	0.386	0	0	0	0.372	0	0.9	0.832	0.992
MTHFD2	PFAS	0	0	0	0	0.156	0	0	0.861	0.874
MTHFD2	IARS	0	0	0	0	0.594	0	0	0.431	0.753
MTHFD2	ASNS	0	0	0	0	0.641	0	0	0.434	0.783
MYBBP1A	BOP1	0	0	0	0	0.406	0.62	0	0	0.759
MYBBP1A	LYAR	0	0	0	0	0.416	0.663	0	0	0.79
MYBL2	CDC6	0	0	0	0	0.687	0	0	0.34	0.779
MYC	ELF3	0	0	0	0	0	0.621	0	0.283	0.709
MYC	CLU	0	0	0	0	0	0	0.9	0.149	0.909
MYC	CCND3	0	0	0	0	0	0	0	0.886	0.886
MYC	RPL11	0	0	0	0	0	0.621	0.9	0.821	0.992
MYC	FOSL1	0	0	0	0	0	0.17	0.9	0.341	0.937
MYC	NDUFAF2	0	0	0	0	0	0	0.9	0.828	0.981
MYC	LRIG1	0	0	0	0	0	0	0	0.809	0.809
MYC	PFKM	0	0	0	0	0	0	0.9	0.164	0.91
MYC	RCC1	0	0	0	0	0	0	0.9	0.854	0.984
MYC	CDC6	0	0	0	0	0	0.621	0	0.332	0.729
MYC	CCND2	0	0	0	0	0	0	0.9	0.902	0.989
MYC	E2F1	0	0	0	0	0	0	0.9	0.877	0.986
MYC	CDK4	0	0	0	0	0	0.627	0.9	0.924	0.996
MYC	NOTCH1	0	0	0	0	0	0	0.9	0.541	0.951
MYC	FOS	0	0	0	0	0	0	0	0.946	0.946
MYC	NDRG1	0	0	0	0	0	0	0.9	0.833	0.982
MYC	CSF3	0	0	0	0	0	0	0	0.847	0.847
MYC	CEBPB	0	0	0	0	0	0.666	0	0.204	0.717
MYC	RUVBL1	0	0	0	0	0	0.861	0.9	0.162	0.986
MYC	GAS1	0	0	0	0	0	0	0	0.835	0.835
MYC	JUND	0	0	0	0	0	0	0.9	0.244	0.919
MYLK	TPM1	0	0	0	0	0.159	0	0.9	0.612	0.962
MYLK	MYL9	0	0	0	0	0.397	0	0.8	0.874	0.982
MYLK	AURKB	0	0	0	0.81	0	0	0.9	0.117	0.901
MYLK	PAK4	0	0	0	0.622	0	0	0.8	0.211	0.811
NAT10	NIP7	0	0	0	0	0.79	0	0	0	0.79
NAT10	BYSL	0	0	0	0	0.823	0.597	0	0.433	0.954
NAT10	DIMT1L	0	0	0	0	0.717	0	0	0.119	0.734
NCAPD3	NCAPH	0	0	0	0	0.479	0	0.9	0.221	0.953

Node1	Node2	Neighborhood	Fusion	Cooccurence	Homology	Coexpression	Experimental	Knowledge	Textmining	Combined_score
NCAPH	CDC6	0	0	0	0	0.895	0	0	0.145	0.904
NCAPH	TRIP13	0	0	0	0	0.821	0	0	0.114	0.831
NCAPH	CENPM	0	0	0	0	0.863	0	0	0	0.863
NCAPH	HELLS	0	0	0	0	0.645	0	0	0.27	0.724
NDRG1	EGR1	0	0	0	0	0	0	0	0.833	0.833
NDRG1	ODC1	0	0	0	0	0	0	0	0.8	0.8
NEDD4L	UBE2S	0	0	0	0	0	0	0	0.751	0.75
NEDD4L	SCNN1G	0	0	0	0	0	0.995	0.8	0.676	0.999
NGFRAP1	NGFR	0	0	0	0	0	0.834	0.9	0.534	0.991
NGFRAP1	SORT1	0	0	0	0	0	0	0.9	0	0.899
NIP7	BYSL	0	0	0	0	0.846	0	0	0.106	0.853
NIP7	POLR2E	0	0	0	0	0.788	0	0	0.133	0.804
NIP7	DIMT1L	0	0	0	0	0.863	0	0	0.217	0.885
NLE1	GTPBP4	0	0	0	0	0.724	0.48	0	0	0.847
NLE1	NAT10	0	0	0	0	0.708	0	0	0.2	0.751
NLE1	NIP7	0	0	0	0	0.721	0	0	0	0.721
NLE1	BYSL	0	0	0	0	0.774	0	0	0	0.774
NLE1	PNO1	0	0	0	0	0.721	0	0	0.329	0.8
NLE1	ZNF593	0	0	0	0	0.535	0.348	0	0.139	0.703
NLE1	TBL3	0	0	0.476	0.674	0.689	0.288	0	0.148	0.804
NLE1	RPF2	0	0	0	0	0.658	0	0	0.184	0.702
NLE1	NOP2	0	0	0	0	0.698	0	0	0.168	0.732
NOC4L	BYSL	0	0	0	0	0.743	0.405	0	0.293	0.877
NOC4L	NIP7	0	0	0	0	0.742	0	0	0	0.742
NOC4L	EMG1	0	0	0	0	0.6	0.45	0	0.198	0.799
NOC4L	PNO1	0	0	0	0	0.644	0.257	0	0.248	0.774
NOC4L	NAT10	0	0	0	0	0.699	0.458	0	0.332	0.876
NOC4L	DIMT1L	0	0	0	0	0.629	0.451	0	0	0.783
NOLC1	CEBPB	0	0	0	0	0	0.872	0	0	0.873
NOLC1	SNRPD1	0	0	0	0	0.4	0	0	0.553	0.713
NOP2	NAT10	0	0	0	0	0.851	0	0	0.105	0.858
NOP2	DIMT1L	0	0	0	0	0.76	0	0	0.37	0.839
NOP2	POLR1B	0	0	0	0	0.757	0	0	0	0.757
NOP2	MAK16	0	0	0	0	0.809	0	0	0.176	0.832
NOP2	NIP7	0	0	0	0	0.809	0.591	0	0.562	0.961
NOP2	NOP56	0	0	0	0	0.835	0.317	0	0.109	0.886
NOP2	BRIX1	0	0	0	0	0.805	0.544	0	0	0.905
NOP2	EMG1	0	0	0	0	0.795	0	0	0	0.795
NOP2	NOC4L	0	0	0	0	0.74	0	0	0.093	0.748
NOP2	DDX56	0	0	0	0	0.657	0.275	0	0.121	0.751
NOP2	BOP1	0	0	0	0	0.754	0.298	0	0.182	0.839
NOP2	WDR74	0	0	0	0	0.727	0	0	0	0.727
NOP2	MYBBP1A	0	0	0	0	0.679	0.62	0	0.102	0.875
NOP2	EIF3B	0	0	0	0	0.724	0	0	0	0.725
NOP2	PUS1	0	0	0	0	0.737	0	0	0.105	0.749
NOP2	BYSL	0	0	0	0	0.852	0	0	0	0.852
NOP2	PNO1	0	0	0	0	0.805	0	0	0.171	0.828
NOP2	GTPBP4	0	0	0	0	0.782	0.683	0	0.147	0.933
NOP2	TBL3	0	0	0	0	0.755	0	0	0	0.755
NOP56	BYSL	0	0	0	0	0.845	0.348	0	0.236	0.912
NOP56	BRIX1	0	0	0	0	0.834	0.456	0	0.121	0.91
NOP56	EIF3B	0	0	0	0	0.715	0	0	0.08	0.72
NOP56	LYAR	0	0	0	0	0.589	0.421	0	0.167	0.774
NOP56	EMG1	0	0	0	0	0.748	0.435	0	0.261	0.88
NOP56	NAT10	0	0	0	0	0.781	0.604	0	0.265	0.927
NOP56	PNO1	0	0	0	0	0.754	0.125	0	0.259	0.819
NOP56	NOC4L	0	0	0	0	0.558	0.301	0	0.18	0.712
NOP56	MAK16	0	0	0	0	0.787	0	0	0	0.788
NOP56	NOLC1	0	0	0	0	0.637	0.421	0	0.158	0.798
NOP56	BOP1	0	0	0	0	0.702	0.065	0	0	0.703
NOP56	GTPBP4	0	0	0	0	0.681	0.453	0	0.11	0.823
NOP56	ILF2	0	0	0	0	0.5	0.421	0	0.122	0.711
NOP56	NIP7	0	0	0	0	0.81	0	0	0.113	0.821
NOP56	POLR1B	0	0	0.453	0	0.678	0.084	0	0	0.816
NOP56	RPL11	0	0	0.209	0	0.545	0.421	0	0	0.762
NOP56	DIMT1L	0.192	0	0	0	0.695	0	0	0.17	0.768
NOP56	TBL3	0	0	0	0	0.645	0.332	0	0.113	0.761
NOP56	SOX4	0	0	0	0	0	0	0	0.866	0.866
NOTCH1	EFNB2	0	0	0	0	0	0	0	0.907	0.907
NOTCH1	CDH1	0	0	0	0	0	0	0	0.917	0.917
NOTCH1	SOX9	0	0	0	0	0	0	0	0.886	0.886
NR2F2	CEBPB	0	0	0	0	0	0	0.9	0.07	0.9
NTF4	NGFR	0	0	0	0	0	0.621	0.9	0.752	0.989
NUDC	NUF2	0	0	0	0	0	0	0.9	0	0.899
NUDC	CENPK	0	0	0	0	0	0	0.9	0	0.899
NUDC	AURKB	0	0	0	0	0.146	0	0.9	0.071	0.909
NUDC	PLK1	0	0	0	0	0	0.621	0.9	0.885	0.995
NUDC	CENPM	0	0	0	0	0	0	0.9	0	0.899
NUF2	CDC6	0	0	0	0	0.851	0	0	0.185	0.87
NUF2	RAD51	0	0	0	0	0.814	0	0	0.117	0.824
NUF2	CDC45	0	0	0	0	0.89	0	0	0.18	0.904
NUF2	CENPM	0	0	0	0	0.736	0	0.9	0	0.971
NUF2	MCM6	0	0	0	0	0.781	0	0	0.136	0.798
NUF2	TRIP13	0	0	0	0	0.831	0	0	0.236	0.862
NUF2	HELLS	0	0	0	0	0.8	0	0	0.145	0.818
NUF2	CENPK	0	0	0	0	0.888	0	0.9	0.211	0.989

Node1	Node2	Neighborhood	Fusion	Cooccurence	Homology	Coexpression	Experimental	Knowledge	Textmining	Combined_score
NCAPH	CDC6	0	0	0	0	0.895	0	0	0.145	0.904
NCAPH	TRIP13	0	0	0	0	0.821	0	0	0.114	0.831
NCAPH	CENPM	0	0	0	0	0.863	0	0	0	0.863
NCAPH	HELLS	0	0	0	0	0.645	0	0	0.27	0.724
NDRG1	EGR1	0	0	0	0	0	0	0	0.833	0.833
NDRG1	ODC1	0	0	0	0	0	0	0	0.8	0.8
NEDD4L	UBE2S	0	0	0	0	0	0	0	0.751	0.75
NEDD4L	SCNN1G	0	0	0	0	0	0.995	0.8	0.676	0.999
NGFRAP1	NGFR	0	0	0	0	0	0.834	0.9	0.534	0.991
NGFRAP1	SORT1	0	0	0	0	0	0	0.9	0	0.899
NIP7	BYSL	0	0	0	0	0.846	0	0	0.106	0.853
NIP7	POLR2E	0	0	0	0	0.788	0	0	0.133	0.804
NIP7	DIMT1L	0	0	0	0	0.863	0	0	0.217	0.885
NLE1	GTPBP4	0	0	0	0	0.724	0.48	0	0	0.847
NLE1	NAT10	0	0	0	0	0.708	0	0	0.2	0.751
NLE1	NIP7	0	0	0	0	0.721	0	0	0	0.721
NLE1	BYSL	0	0	0	0	0.774	0	0	0	0.774
NLE1	PNO1	0	0	0	0	0.721	0	0	0.329	0.8
NLE1	ZNF593	0	0	0	0	0.535	0.348	0	0.139	0.703
NLE1	TBL3	0	0	0.476	0.674	0.689	0.288	0	0.148	0.804
NLE1	RPF2	0	0	0	0	0.658	0	0	0.184	0.702
NLE1	NOP2	0	0	0	0	0.698	0	0	0.168	0.732
NOC4L	BYSL	0	0	0	0	0.743	0.405	0	0.293	0.877
NOC4L	NIP7	0	0	0	0	0.742	0	0	0	0.742
NOC4L	EMG1	0	0	0	0	0.6	0.45	0	0.198	0.799
NOC4L	PNO1	0	0	0	0	0.644	0.257	0	0.248	0.774
NOC4L	NAT10	0	0	0	0	0.699	0.458	0	0.332	0.876
NOC4L	DIMT1L	0	0	0	0	0.629	0.451	0	0	0.783
NOLC1	CEBPB	0	0	0	0	0	0.872	0	0	0.873
NOLC1	SNRPD1	0	0	0	0	0.4	0	0	0.553	0.713
NOP2	NAT10	0	0	0	0	0.851	0	0	0.105	0.858
NOP2	DIMT1L	0	0	0	0	0.76	0	0	0.37	0.839
NOP2	POLR1B	0	0	0	0	0.757	0	0	0	0.757
NOP2	MAK16	0	0	0	0	0.809	0	0	0.176	0.832
NOP2	NIP7	0	0	0	0	0.809	0.591	0	0.562	0.961
NOP2	NOP56	0	0	0	0	0.835	0.317	0	0.109	0.886
NOP2	BRIX1	0	0	0	0	0.805	0.544	0	0	0.905
NOP2	EMG1	0	0	0	0	0.795	0	0	0	0.795
NOP2	NOC4L	0	0	0	0	0.74	0	0	0.093	0.748
NOP2	DDX56	0	0	0	0	0.657	0.275	0	0.121	0.751
NOP2	BOP1	0	0	0	0	0.754	0.298	0	0.182	0.839
NOP2	WDR74	0	0	0	0	0.727	0	0	0	0.727
NOP2	MYBBP1A	0	0	0	0	0.679	0.62	0	0.102	0.875
NOP2	EIF3B	0	0	0	0	0.724	0	0	0	0.725
NOP2	PUS1	0	0	0	0	0.737	0	0	0.105	0.749
NOP2	BYSL	0	0	0	0	0.852	0	0	0	0.852
NOP2	PNO1	0	0	0	0	0.805	0	0	0.171	0.828
NOP2	GTPBP4	0	0	0	0	0.782	0.683	0	0.147	0.933
NOP2	TBL3	0	0	0	0	0.755	0	0	0	0.755
NOP56	BYSL	0	0	0	0	0.845	0.348	0	0.236	0.912
NOP56	BRIX1	0	0	0	0	0.834	0.456	0	0.121	0.91
NOP56	EIF3B	0	0	0	0	0.715	0	0	0.08	0.72
NOP56	LYAR	0	0	0	0	0.589	0.421	0	0.167	0.774
NOP56	EMG1	0	0	0	0	0.748	0.435	0	0.261	0.88
NOP56	NAT10	0	0	0	0	0.781	0.604	0	0.265	0.927
NOP56	PNO1	0	0	0	0	0.754	0.125	0	0.259	0.819
NOP56	NOC4L	0	0	0	0	0.558	0.301	0	0.18	0.712
NOP56	MAK16	0	0	0	0	0.787	0	0	0	0.788
NOP56	NOLC1	0	0	0	0	0.637	0.421	0	0.158	0.798
NOP56	BOP1	0	0	0	0	0.702	0.065	0	0	0.703
NOP56	GTPBP4	0	0	0	0	0.681	0.453	0	0.11	0.823
NOP56	ILF2	0	0	0	0	0.5	0.421	0	0.122	0.711
NOP56	NIP7	0	0	0	0	0.81	0	0	0.113	0.821
NOP56	POLR1B	0	0	0.453	0	0.678	0.084	0	0	0.816
NOP56	RPL11	0	0	0.209	0	0.545	0.421	0	0	0.762
NOP56	DIMT1L	0.192	0	0	0	0.695	0	0	0.17	0.768
NOP56	TBL3	0	0	0	0	0.645	0.332	0	0.113	0.761
NOP56	SOX4	0	0	0	0	0	0	0	0.866	0.866
NOTCH1	EFNB2	0	0	0	0	0	0	0	0.907	0.907
NOTCH1	CDH1	0	0	0	0	0	0	0	0.917	0.917
NOTCH1	SOX9	0	0	0	0	0	0	0	0.886	0.886
NR2F2	CEBPB	0	0	0	0	0	0	0.9	0.07	0.9
NTF4	NGFR	0	0	0	0	0	0.621	0.9	0.752	0.989
NUDC	NUF2	0	0	0	0	0	0	0.9	0	0.899
NUDC	CENPK	0	0	0	0	0	0	0.9	0	0.899
NUDC	AURKB	0	0	0	0	0.146	0	0.9	0.071	0.909
NUDC	PLK1	0	0	0	0	0	0.621	0.9	0.885	0.995
NUDC	CENPM	0	0	0	0	0	0	0.9	0	0.899
NUF2	CDC6	0	0	0	0	0.851	0	0	0.185	0.87
NUF2	RAD51	0	0	0	0	0.814	0	0	0.117	0.824
NUF2	CDC45	0	0	0	0	0.89	0	0	0.18	0.904
NUF2	CENPM	0	0	0	0	0.736	0	0.9	0	0.971
NUF2	MCM6	0	0	0	0	0.781	0	0	0.136	0.798
NUF2	TRIP13	0	0	0	0	0.831	0	0	0.236	0.862
NUF2	HELLS	0	0	0	0	0.8	0	0	0.145	0.818
NUF2	CENPK	0	0	0	0	0.888	0	0.9	0.211	0.989

Node1	Node2	Neighborhood	Fusion	Cooccurence	Homology	Coexpression	Experimental	Knowledge	Textmining	Combined_score
NUF2	NCAPH	0	0	0	0	0.85	0	0	0.113	0.858
NUP155	NUP88	0	0	0	0	0.22	0	0.9	0.336	0.941
NUP155	TRIP13	0	0	0	0	0.786	0	0	0	0.787
NUP93	GEMIN5	0	0	0	0	0.156	0	0.9	0	0.909
NUP93	NUP155	0	0	0	0	0.417	0	0.9	0.75	0.983
NUP93	NUP88	0	0	0	0	0.239	0	0.9	0.504	0.957
OCLN	CSNK2A1	0	0	0	0	0	0	0.8	0	0.8
OCLN	CDH1	0	0	0	0	0	0	0	0.784	0.784
OCLN	CLDN1	0	0	0	0	0	0	0.8	0.786	0.954
ODC1	MMP2	0	0	0	0	0	0	0	0.81	0.81
ODC1	CSNK2A1	0	0	0	0	0	0	0	0.817	0.817
P2RY6	ANXA1	0	0	0	0	0	0	0.9	0	0.899
P2RY6	PIK3R1	0	0	0	0	0	0	0.9	0	0.899
P2RY6	BDKRB2	0	0	0	0.775	0	0	0.9	0.104	0.9
P2RY6	PTAFR	0	0	0	0.756	0	0	0.9	0.167	0.902
PAK4	RAC3	0	0	0	0	0	0.9	0.8	0.464	0.987
PAK4	ITGAV	0	0	0	0	0	0.619	0	0.805	0.921
PDK1	PIK3R1	0	0	0	0	0	0	0.9	0	0.899
PFAS	PPAT	0.626	0	0	0	0.774	0	0	0.764	0.977
PFDN2	POLR2E	0	0	0	0	0.555	0	0	0.722	0.868
PFKM	PYGB	0	0	0	0	0.086	0	0	0.768	0.773
PHGDH	ASNS	0	0	0	0	0.52	0	0	0.449	0.718
PHGDH	SHMT2	0.077	0	0	0	0.402	0	0	0.812	0.882
PI4K2A	PLCD3	0	0	0	0	0	0	0.9	0	0.899
PI4K2A	PIK3R1	0	0	0	0	0	0	0.9	0	0.899
PIK3R1	EFNB2	0	0	0	0	0	0	0.9	0.078	0.901
PIK3R1	CSK	0	0	0	0.552	0	0	0.8	0	0.8
PIK3R1	ANXA1	0	0	0	0	0	0	0.9	0	0.899
PIK3R1	ITGAV	0	0	0	0	0	0	0.9	0	0.899
PIK3R1	NGFR	0	0	0	0	0	0	0.9	0	0.899
PKD1	CDH1	0	0	0	0	0	0.619	0	0.878	0.95
PLAU	MMP2	0	0	0	0	0	0	0	0.751	0.75
PLAU	PIK3R1	0	0	0	0	0	0	0.9	0.08	0.901
PLAU	FOSL1	0	0	0	0	0.149	0	0.9	0.346	0.936
PLAU	FOS	0	0	0	0	0	0	0.9	0.515	0.948
PLAU	JUND	0	0	0	0	0	0	0.9	0.274	0.922
PLAU	E2F1	0	0	0	0	0	0	0.9	0	0.899
PLAU	MRC2	0	0	0	0	0	0	0	0.885	0.885
PLAU	ITGAV	0	0	0	0	0	0	0.9	0.374	0.933
PLCD3	PIK3R1	0	0	0	0	0	0	0.9	0	0.899
PLD1	CHPT1	0	0	0	0	0	0	0.9	0	0.899
PLK1	CDC45	0	0	0	0	0.772	0	0	0.372	0.847
PLK1	RAD51	0	0	0	0	0.502	0	0	0.866	0.929
PLK1	CENPM	0	0	0	0	0.535	0	0.9	0.146	0.954
PLK1	CDC6	0	0	0	0	0.655	0.621	0	0.885	0.982
PLK1	NUF2	0	0	0	0	0.595	0	0.9	0.411	0.972
PLK1	CDCA5	0	0	0	0	0.62	0	0	0.854	0.941
PLK1	NCAPH	0	0	0	0	0.632	0	0	0.235	0.7
PLK1	CENPK	0	0	0	0	0.303	0	0.9	0.179	0.934
PLK1	TRIP13	0	0	0	0	0.67	0	0	0.147	0.7
PLOD2	MLL5	0	0	0	0	0	0	0.9	0	0.899
PLXNA2	RAC3	0	0	0	0	0	0	0.8	0.144	0.817
PNO1	NIP7	0	0	0	0	0.907	0	0	0	0.907
PNO1	EMG1	0.199	0	0	0	0.826	0.645	0	0.32	0.959
PNO1	NAT10	0	0	0	0	0.769	0	0	0.478	0.871
PNO1	BYSL	0	0	0	0	0.84	0.541	0	0.291	0.941
PNO1	DIMT1L	0	0	0	0	0.841	0.66	0	0.144	0.947
PNO1	POLR1B	0	0	0	0	0.732	0	0	0	0.732
PNP	GDA	0.598	0	0	0	0.239	0	0.9	0.562	0.983
PNP	PYGB	0.146	0	0	0	0	0	0	0.672	0.702
PNP	UPP1	0	0	0	0	0	0	0.9	0.659	0.963
POLR1B	BYSL	0	0	0	0	0.704	0	0	0	0.704
POLR1B	NAT10	0	0	0	0	0.779	0	0	0	0.78
POLR1B	DIMT1L	0	0	0	0	0.706	0	0	0	0.707
POLR1B	POLR2E	0.268	0	0	0	0.397	0.508	0	0.392	0.84
POLR1B	NIP7	0	0	0	0	0.796	0	0	0.197	0.825
POLR3G	POLR2E	0	0	0	0	0.089	0.12	0.9	0.341	0.935
POLR3G	POLR3K	0	0	0	0	0	0.096	0.9	0.23	0.92
POLR3K	POLR2E	0	0	0	0	0.461	0.589	0.9	0.315	0.981
PPAT	ASNS	0.253	0	0	0	0	0	0	0.732	0.786
PPAT	MARS	0	0	0	0	0.256	0	0	0.648	0.721
PRC1	MCM6	0	0	0	0	0.828	0	0	0.236	0.86
PRC1	CENPM	0	0	0	0	0.767	0	0	0.125	0.783
PRC1	UBE2T	0	0	0	0	0.774	0	0	0	0.774
PRC1	RAD51	0	0	0	0	0.737	0	0	0.195	0.774
PRC1	GINS2	0	0	0	0	0.772	0	0	0.163	0.796
PRC1	KIF2C	0	0	0	0	0.887	0	0	0.369	0.924
PRC1	CDCA5	0	0	0	0	0.909	0	0	0.197	0.922
PRC1	TRIP13	0	0	0	0	0.904	0	0	0.214	0.919
PRC1	PLK1	0	0	0	0	0.668	0.845	0.72	0.43	0.99
PRC1	NCAPH	0	0	0	0	0.848	0	0	0.181	0.868
PRC1	TACC3	0	0	0	0	0.716	0	0	0.235	0.768
PRC1	DNMT1	0	0	0	0	0.585	0	0	0.368	0.72
PRC1	MCM5	0	0	0	0	0.749	0	0	0.186	0.782
PRC1	AURKB	0	0	0	0	0.724	0	0	0.467	0.843
PRC1	DTL	0	0	0	0	0.875	0	0	0.159	0.888

Node1	Node2	Neighborhood	Fusion	Cooccurence	Homology	Coexpression	Experimental	Knowledge	Textmining	Combined_score
RRM2	CENPM	0	0	0	0	0.809	0	0	0	0.809
RRM2	RNASEH2A	0	0	0	0	0.809	0	0	0	0.809
RRM2	NUF2	0	0	0	0	0.836	0	0	0.161	0.854
RRM2	HELLS	0	0	0	0	0.756	0	0	0.07	0.758
RRM2	PLK1	0	0	0	0	0.686	0	0	0.289	0.762
RRM2	RAD51	0	0	0	0	0.839	0	0	0.269	0.874
RUNX1	AREG	0	0	0	0	0	0	0	0.814	0.814
RUNX1	CCND2	0	0	0	0	0	0.621	0	0.273	0.705
RUVBL1	NAT10	0	0	0.376	0	0.554	0	0	0	0.703
RUVBL1	POLR2E	0	0	0	0	0.48	0.586	0	0.358	0.842
RUVBL1	HCFC1	0	0	0	0	0	0	0.9	0	0.899
RUVBL1	CENPK	0	0	0	0	0.109	0	0.9	0	0.904
RUVBL1	C21orf45	0	0	0	0	0.134	0	0.9	0	0.907
S100A7	RNASE7	0	0	0	0	0	0	0	0.75	0.75
S100A8	CSF3	0	0	0	0	0	0	0	0.829	0.829
SDCCAG8	PLK1	0	0	0	0	0	0	0.9	0.071	0.9
SERPINE2	PLAU	0	0	0	0	0	0.621	0	0.429	0.769
SH3KBP1	PIK3R1	0	0	0	0	0	0.981	0.9	0	0.998
SHMT2	FARSA	0.167	0	0	0	0.302	0	0	0.636	0.76
SHMT2	PPAT	0.107	0	0	0	0.483	0	0	0.52	0.748
SIAH2	SNCAIP	0	0	0	0	0	0.635	0	0.866	0.947
SIAH2	HIPK2	0	0	0	0	0	0.619	0	0.378	0.747
SIRPA	F3	0	0	0	0	0	0.937	0	0.102	0.94
SLC16A2	SLC6A15	0	0	0	0	0	0	0	0.778	0.778
SLU7	PPIG	0	0	0	0	0.229	0.998	0	0.086	0.998
SMPD1	TNFSF10	0	0	0	0	0	0	0.9	0.23	0.917
SMPD1	SMPD2	0	0	0	0	0	0	0.8	0.752	0.947
SMPD1	BIRC3	0	0	0	0	0	0	0.9	0.113	0.905
SMPD1	PIK3R1	0	0	0	0	0	0	0.9	0	0.899
SMPD2	NGFR	0	0	0	0	0	0	0.9	0.29	0.924
SMPD2	SORT1	0	0	0	0	0	0	0.9	0	0.899
SNRNP70	POLR2E	0	0	0	0	0.247	0	0.9	0	0.919
SNRPA1	SNRNP70	0	0	0	0	0.126	0.353	0.9	0.156	0.942
SNRPA1	POLR2E	0	0	0	0	0.397	0	0.9	0	0.935
SNRPD1	SNRNP70	0	0	0	0	0.076	0.911	0	0.517	0.955
SNRPD1	GEMIN5	0	0	0	0	0.125	0.596	0	0.477	0.789
SNRPD1	NIP7	0	0	0	0	0.779	0	0	0	0.779
SORT1	NGFR	0	0	0	0	0	0.741	0.9	0.898	0.997
SQSTM1	NGFR	0	0	0	0	0	0	0.9	0	0.899
SQSTM1	TARDBP	0	0	0	0	0	0.846	0	0.31	0.886
SRGN	SPARC	0	0	0	0	0	0	0.9	0	0.899
SRM	ODC1	0.508	0	0	0	0	0.144	0.9	0.822	0.99
SUV39H1	SIRT2	0	0	0	0	0	0	0.9	0.379	0.933
SUV39H1	PLOD2	0	0	0	0	0	0	0.9	0	0.899
SUV39H1	E2F1	0	0	0	0	0	0	0.9	0.119	0.905
SUV39H1	CCND3	0	0	0	0	0	0	0.9	0.112	0.905
SUV39H1	CDK4	0	0	0	0	0	0	0.9	0.242	0.919
SUV39H1	DNMT1	0	0	0	0	0	0.846	0	0.927	0.988
SUV39H1	FTSJ1	0	0	0	0	0.736	0	0	0	0.736
SUV39H1	CCND2	0	0	0	0	0	0	0.9	0.079	0.901
SUV39H1	RUNX1	0	0	0	0	0	0.845	0	0.838	0.973
TACC3	CDC45	0	0	0	0	0.796	0	0	0.119	0.808
TACC3	AURKB	0	0	0	0	0.554	0	0	0.379	0.704
TACSTD2	C1QBP	0	0	0	0	0	0	0	0.732	0.732
TBL3	BOP1	0	0	0	0.498	0.76	0	0	0.118	0.768
TBL3	PNO1	0	0	0	0	0.696	0	0	0.357	0.791
TBL3	BYSL	0	0	0	0	0.75	0.348	0	0.324	0.875
TBL3	EMG1	0	0	0	0	0.655	0.495	0	0.36	0.873
TBL3	NOC4L	0	0	0	0	0.765	0.344	0	0.249	0.868
TBL3	NAT10	0	0	0	0	0.792	0.493	0	0.366	0.924
TBL3	NIP7	0	0	0	0	0.723	0	0	0	0.723
TCOF1	NOP56	0	0	0	0	0.199	0.421	0	0.915	0.955
TGFB2	CDK4	0	0	0	0	0	0	0	0.82	0.82
TGFB2	CLU	0	0	0	0	0	0	0.9	0.085	0.902
TGFB2	SRGN	0	0	0	0	0	0	0.9	0	0.899
TGFB2	SPARC	0	0	0	0	0	0	0.9	0.22	0.916
TGFB2	MRC2	0	0	0	0	0	0	0.8	0	0.8
TGFB2	MMP2	0	0	0	0	0	0	0	0.859	0.859
TIMM44	TOMM40	0	0	0	0	0.251	0	0.9	0.752	0.978
TIMP3	MMP2	0	0	0	0	0.125	0.846	0	0.752	0.962
TNC	ITGAV	0	0	0	0	0	0	0.9	0.318	0.927
TNC	CDH1	0	0	0	0	0	0	0	0.878	0.878
TP53INP1	HIPK2	0	0	0	0	0	0.621	0	0.864	0.945
TRIB3	ASNS	0	0	0	0	0.45	0	0	0.502	0.707
TUBB2C	TUBA1C	0	0	0	0.922	0.124	0	0.8	0.378	0.817
TUBB2C	TUBA1B	0	0	0	0.922	0.348	0	0.8	0.129	0.861
UBE2E2	ISG15	0	0	0	0	0	0.846	0	0	0.846
UBE2H	RAD51	0	0	0	0	0	0	0	0.874	0.874
UBE2T	CENPM	0	0	0	0	0.732	0	0	0	0.732
UBE2T	CDCA5	0	0	0	0	0.886	0	0	0.104	0.89
UBE2T	CENPK	0	0	0	0	0.777	0	0	0	0.776
UBE2T	CDC45	0	0	0	0	0.868	0	0	0	0.867
UBE2T	CDC6	0	0	0	0	0.801	0	0	0	0.8
UBE2T	ATRIP	0	0	0	0	0	0	0.9	0	0.899
UBE2T	EXO1	0	0	0	0	0.812	0	0	0	0.812
UBE2T	TRIP13	0	0	0	0	0.719	0	0	0	0.718

Node1	Node2	Neighborhood	Fusion	Cooccurence	Homology	Coexpression	Experimental	Knowledge	Textmining	Combined_score
RRM2	CENPM	0	0	0	0	0.809	0	0	0	0.809
RRM2	RNASEH2A	0	0	0	0	0.809	0	0	0	0.809
RRM2	NUF2	0	0	0	0	0.836	0	0	0.161	0.854
RRM2	HELLS	0	0	0	0	0.756	0	0	0.07	0.758
RRM2	PLK1	0	0	0	0	0.686	0	0	0.289	0.762
RRM2	RAD51	0	0	0	0	0.839	0	0	0.269	0.874
RUNX1	AREG	0	0	0	0	0	0	0	0.814	0.814
RUNX1	CCND2	0	0	0	0	0	0.621	0	0.273	0.705
RUVBL1	NAT10	0	0	0.376	0	0.554	0	0	0	0.703
RUVBL1	POLR2E	0	0	0	0	0.48	0.586	0	0.358	0.842
RUVBL1	HCFC1	0	0	0	0	0	0	0.9	0	0.899
RUVBL1	CENPK	0	0	0	0	0.109	0	0.9	0	0.904
RUVBL1	C21orf45	0	0	0	0	0.134	0	0.9	0	0.907
S100A7	RNASE7	0	0	0	0	0	0	0	0.75	0.75
S100A8	CSF3	0	0	0	0	0	0	0	0.829	0.829
SDCCAG8	PLK1	0	0	0	0	0	0	0.9	0.071	0.9
SERPINE2	PLAU	0	0	0	0	0	0.621	0	0.429	0.769
SH3KBP1	PIK3R1	0	0	0	0	0	0.981	0.9	0	0.998
SHMT2	FARSA	0.167	0	0	0	0.302	0	0	0.636	0.76
SHMT2	PPAT	0.107	0	0	0	0.483	0	0	0.52	0.748
SIAH2	SNCAIP	0	0	0	0	0	0.635	0	0.866	0.947
SIAH2	HIPK2	0	0	0	0	0	0.619	0	0.378	0.747
SIRPA	F3	0	0	0	0	0	0.937	0	0.102	0.94
SLC16A2	SLC6A15	0	0	0	0	0	0	0	0.778	0.778
SLU7	PPIG	0	0	0	0	0.229	0.998	0	0.086	0.998
SMPD1	TNFSF10	0	0	0	0	0	0	0.9	0.23	0.917
SMPD1	SMPD2	0	0	0	0	0	0	0.8	0.752	0.947
SMPD1	BIRC3	0	0	0	0	0	0	0.9	0.113	0.905
SMPD1	PIK3R1	0	0	0	0	0	0	0.9	0	0.899
SMPD2	NGFR	0	0	0	0	0	0	0.9	0.29	0.924
SMPD2	SORT1	0	0	0	0	0	0	0.9	0	0.899
SNRNP70	POLR2E	0	0	0	0	0.247	0	0.9	0	0.919
SNRPA1	SNRNP70	0	0	0	0	0.126	0.353	0.9	0.156	0.942
SNRPA1	POLR2E	0	0	0	0	0.397	0	0.9	0	0.935
SNRPD1	SNRNP70	0	0	0	0	0.076	0.911	0	0.517	0.955
SNRPD1	GEMIN5	0	0	0	0	0.125	0.596	0	0.477	0.789
SNRPD1	NIP7	0	0	0	0	0.779	0	0	0	0.779
SORT1	NGFR	0	0	0	0	0	0.741	0.9	0.898	0.997
SQSTM1	NGFR	0	0	0	0	0	0	0.9	0	0.899
SQSTM1	TARDBP	0	0	0	0	0	0.846	0	0.31	0.886
SRGN	SPARC	0	0	0	0	0	0	0.9	0	0.899
SRM	ODC1	0.508	0	0	0	0	0.144	0.9	0.822	0.99
SUV39H1	SIRT2	0	0	0	0	0	0	0.9	0.379	0.933
SUV39H1	PLOD2	0	0	0	0	0	0	0.9	0	0.899
SUV39H1	E2F1	0	0	0	0	0	0	0.9	0.119	0.905
SUV39H1	CCND3	0	0	0	0	0	0	0.9	0.112	0.905
SUV39H1	CDK4	0	0	0	0	0	0	0.9	0.242	0.919
SUV39H1	DNMT1	0	0	0	0	0	0.846	0	0.927	0.988
SUV39H1	FTSJ1	0	0	0	0	0.736	0	0	0	0.736
SUV39H1	CCND2	0	0	0	0	0	0	0.9	0.079	0.901
SUV39H1	RUNX1	0	0	0	0	0	0.845	0	0.838	0.973
TACC3	CDC45	0	0	0	0	0.796	0	0	0.119	0.808
TACC3	AURKB	0	0	0	0	0.554	0	0	0.379	0.704
TACSTD2	C1QBP	0	0	0	0	0	0	0	0.732	0.732
TBL3	BOP1	0	0	0	0.498	0.76	0	0	0.118	0.768
TBL3	PNO1	0	0	0	0	0.696	0	0	0.357	0.791
TBL3	BYSL	0	0	0	0	0.75	0.348	0	0.324	0.875
TBL3	EMG1	0	0	0	0	0.655	0.495	0	0.36	0.873
TBL3	NOC4L	0	0	0	0	0.765	0.344	0	0.249	0.868
TBL3	NAT10	0	0	0	0	0.792	0.493	0	0.366	0.924
TBL3	NIP7	0	0	0	0	0.723	0	0	0	0.723
TCOF1	NOP56	0	0	0	0	0.199	0.421	0	0.915	0.955
TGFB2	CDK4	0	0	0	0	0	0	0	0.82	0.82
TGFB2	CLU	0	0	0	0	0	0	0.9	0.085	0.902
TGFB2	SRGN	0	0	0	0	0	0	0.9	0	0.899
TGFB2	SPARC	0	0	0	0	0	0	0.9	0.22	0.916
TGFB2	MRC2	0	0	0	0	0	0	0.8	0	0.8
TGFB2	MMP2	0	0	0	0	0	0	0	0.859	0.859
TIMM44	TOMM40	0	0	0	0	0.251	0	0.9	0.752	0.978
TIMP3	MMP2	0	0	0	0	0.125	0.846	0	0.752	0.962
TNC	ITGAV	0	0	0	0	0	0	0.9	0.318	0.927
TNC	CDH1	0	0	0	0	0	0	0	0.878	0.878
TP53INP1	HIPK2	0	0	0	0	0	0.621	0	0.864	0.945
TRIB3	ASNS	0	0	0	0	0.45	0	0	0.502	0.707
TUBB2C	TUBA1C	0	0	0	0.922	0.124	0	0.8	0.378	0.817
TUBB2C	TUBA1B	0	0	0	0.922	0.348	0	0.8	0.129	0.861
UBE2E2	ISG15	0	0	0	0	0	0.846	0	0	0.846
UBE2H	RAD51	0	0	0	0	0	0	0	0.874	0.874
UBE2T	CENPM	0	0	0	0	0.732	0	0	0	0.732
UBE2T	CDCA5	0	0	0	0	0.886	0	0	0.104	0.89
UBE2T	CENPK	0	0	0	0	0.777	0	0	0	0.776
UBE2T	CDC45	0	0	0	0	0.868	0	0	0	0.867
UBE2T	CDC6	0	0	0	0	0.801	0	0	0	0.8
UBE2T	ATRIP	0	0	0	0	0	0	0.9	0	0.899
UBE2T	EXO1	0	0	0	0	0.812	0	0	0	0.812
UBE2T	TRIP13	0	0	0	0	0.719	0	0	0	0.718

Node1	Node2	Neighborhood	Fusion	Cooccurence	Homology	Coexpression	Experimental	Knowledge	Textmining	Combined_score
UBE2T	RRM2	0	0	0	0	0.802	0	0	0	0.802
UBE2T	NUF2	0	0	0	0	0.91	0	0	0	0.909
UBE2T	AURKB	0	0	0	0	0.707	0	0	0	0.706
UBE2T	DTL	0	0	0	0	0.848	0	0	0	0.847
UBE2T	GINS2	0	0	0	0	0.738	0	0	0	0.738
UBE2T	MCM6	0	0	0	0	0.785	0	0	0	0.784
UBE2T	RAD51	0	0	0	0	0.772	0	0	0.113	0.784
UBE2T	NCAPH	0	0	0	0	0.723	0	0	0	0.722
UBE2T	MCM10	0	0	0	0	0.702	0	0	0	0.702
UBIAD1	SLC16A4	0	0	0	0	0	0	0.9	0	0.899
UGCG	PIK3R1	0	0	0	0	0	0	0.9	0	0.899
UGCG	SMPD2	0	0	0	0	0	0	0.9	0.541	0.951
UGCG	SMPD1	0	0	0	0	0	0	0.9	0.463	0.942
USP8	CHMP4C	0	0	0	0	0	0.839	0	0	0.839
VAV3	EFNA1	0	0	0	0	0	0	0.9	0.269	0.921
VAV3	ITGAV	0	0	0	0	0	0	0.9	0.297	0.924
VAV3	PIK3R1	0	0	0	0.498	0	0.845	0	0.086	0.847
YARS	FARSA	0.266	0	0	0	0.679	0	0	0.826	0.953
YARS	MARS	0	0.022	0	0	0.759	0	0	0.892	0.972
YARS	ASNS	0	0	0	0	0.61	0	0	0.412	0.756
ZFP36	DUSP1	0	0	0	0	0.828	0	0	0.068	0.829
ZFP36	EGR1	0	0	0	0	0.602	0	0	0.349	0.724
ZNF593	GTPBP4	0	0	0	0	0.592	0.465	0	0.188	0.798
ZNF593	NIP7	0	0	0	0	0.711	0	0	0	0.711
ZNF593	PNO1	0	0	0	0	0.741	0	0	0	0.742
ZNF593	BYSL	0	0	0	0	0.761	0	0	0	0.761
ZNF622	MYBL2	0	0	0	0	0	0.621	0	0.758	0.902

Supplementary Table 3

AURKA interacting proteins and MYC promoter binding proteins are shown.

AURKA interacting protein	*MYC* promoter region binding protein			
16 KDA PROTEIN.	α-Enolase	ETS-2	MIBP1	Smad-4
22 KDA PROTEIN.	AhR	FBI-1	MSSP-1	Sp1
ACTA1	AP-1	FBP	MSSP-2	Sp3
ACTA2	AP-2	FBP2	Mxi1	STAT1
ACTB	AR	FBP3	NF1	STAT3
ACTIN-LIKE PROTEIN	ARID1A	FIR	NFATc1	STAT4
CAP1	β-Catenin	FOXM1c	NF-κB	STAT5
CAP2	Blimp-1	FOXO3a	NM23-H2	TCF-4
CDNA FLJ77784	BMAL1/NPAS2	GATA-1	Notch1	THZif-1
DDX17	B-Myb	γ-Catenin	NSEP-1	TR/RXR
DDX3X	C/EBPα	GR	Oct	USF
DDX5	C/EBPβ	hnRNP A1	Ovol1	WT1
EEF2	c-Fos	hnRNP K	p105/p115	YY1
EIF4B	c-Jun	HOXB4	p30	ZF5
FUS	c-Myb	HOXB4	p50	
HCCA2	c-Myc	ICAP-1	p53	
HNRNPA1	CNBP	ID2	p73	
HNRNPK	c-Rel	JunD	p97	
HNRNPL	CSL	KLF11	Pitx2	
JAK1	CTCF	LEF-1	PLZF	
PABPC1	CUT	LR1	PTTG	
PFKFB3	DP-1	Max	PU.1	
PRMT5	E2F-1	MAZ	Pur	
RARS	E2F-2	MAZi	RelA	
STK38	E2F-3	MAZR	RelB	
THOC4	E2F-4	MBP-1	RFX1	
TUBB3	E2F-5	ME1a1	RFX1	
TUBB4	E2F-6	ME1a2	SATB1	
TUT1	ER	Mel-18	Smad-2	
WDR77	ETS-1	METS	Smad3	

Supplementary Table 4

The primers used in quantitative PCR are shown.

Genes	Forward Primer (5' > 3')	Reverse Primer (5' > 3')
AURKA	GGAATATGCACCACTTGGAACA	TAAGACAGGGCATTTGCCAAT
CCNA1	ACATGGATGAACTAGAGCAGGG	ACATGGATGAACTAGAGCAGGG
CCNA2	TGGAAAGCAAACAGTAAACAGCC	GGGCATCTTCACGCTCTATTT
CCND1	GCTGCGAAGTGGAAACCATC	CCTCCTTCTGCACACATTTGAA
CCND2	ACCTTCCGCAGTGCTCCTA	CCCAGCCAAGAAACGGTCC
CCND3	TACCCGCCATCCATGATCG	AGGCAGTCCACTTCAGTGC
CCNE1	ACTCAACGTGCAAGCCTCG	GCTCAAGAAAGTGCTGATCCC
CCNE2	TCAAGACGAAGTAGCCGTTTAC	TGACATCCTGGGTAGTTTTCCTC
CD24	CTCCTACCCACGCAGATTTATTC	AGAGTGAGACCACGAAGAGAC
CD44	CTGCCGCTTTGCAGGTGTA	CATTGTGGGCAAGGTGCTATT
CDC45	TTCGTGTCCGATTTCCGCAAA	TGGAACCAGCGTATATTGCAC
CDC6	CCAGGCACAGGCTACAATCAG	AACAGGTTACGGTTTGGACATT
CDH1	CGAGAGCTACACGTTCACGG	GGGTGTCGAGGGAAAAATAGG
CDH2	AGCCAACCTTAACTGAGGAGT	GGCAAGTTGATTGGAGGGATG
CDKN1A	TGTCCGTCAGAACCCATGC	AAAGTCGAAGTTCCATCGCTC
CDKN1B	TAATTGGGGCTCCGGCTAACT	TGCAGGTCGCTTCCTTATTCC
E2F1	ACGTGACGTGTCAGGACCT	GATCGGGCCTTGTTTGCTCTT
MCM10	AAGCCTTCTCTGGTCTGCG	CTGTGGCGTAACCTTCTTCAA
MCM2	ATGGCGGAATCATCGGAATCC	GGTGAGGGCATCAGTACGC
MCM3	GGCCTCCATTGATGCTACCTA	GACGACTTTGGGACGAACTAG
MCM5	AGCATTCGTAGCCTGAAGTCG	CGGCACTGGATAGAGATGCG
MCM6	GAGGAACTGATTCGTCCTGAGA	CAAGGCCCGACACAGGTAAG
MCM7	GCCTGTGGGAAATATCCCTCG	GTACCACCTGTCGGAACCC
MYC	GCCACGTCTCCACACATCAG	TGGTGCATTTTCGGTTGTTG
VIM	GACGCCATCAACACCGAGTT	CTTTGTCGTTGGTTAGCTGGT
GAPDH	TGCACCACCAACTGCTTAGC	GGCATGGACTGTGGTCATGAG

SUPPLEMENTARY METHODS

Immunofluorescence (IF) Analysis

Cells (5×10^3 cells per well) were seeded into 6-well plate. The next day, cells were rinsed with ice-cold PBS and fixed with 5% paraformaldehyde for 10 min at RT. Then, cells were treated with 1% BSA (in PBS) plus 0.2% Triton X-100 for 15 min. Cells were subjected to immunofluorescence staining with indicated antibodies (1:200) for 1 h at RT. Cells were then washed with PBS three times for 10 min each. Cells were incubated with indicated secondary antibody (1:200) at RT for 1 h. Cells were washed with cold PBS three times for 10 min each, and incubated with DAPI (0.1μg/ml) to stain the nucleus. The cells were examined by confocal microscopy (Olympus).
